# Testing a Prespecified Matrix Transcriptional Programme in Myeloid Cells from Proliferative Vitreoretinopathy Membranes: A Donor-Aware Computational Single-Cell Study

**DOI:** 10.3390/bioengineering13101185

**Published:** 2026-10-12

**Authors:** Georgios D. Panos

**Affiliations:** 1 Division of Ophthalmology and Visual Sciences, School of Medicine, University of Nottingham, Nottingham NG7 2UH, UK; gdpanos@gmail.com; Tel.: +44-115-924-9924; 2 First Department of Ophthalmology, AHEPA University Hospital, School of Medicine, Aristotle University of Thessaloniki, 54636 Thessaloniki, Greece

**Keywords:** proliferative vitreoretinopathy, proliferative diabetic retinopathy, single-cell RNA sequencing, macrophages, extracellular matrix, fibrosis, ambient RNA, pseudoreplication, reproducibility

## Abstract

Whether myeloid cells in proliferative vitreoretinopathy (PVR) membranes directly transcribe a coherent extracellular matrix (ECM) programme remains uncertain. We tested a prespecified seven-gene programme in a discovery cohort (GSE294329; five PVR donors) and an independent epiretinal PVR cohort (SCP2582; five donors), with GSE165784 (five public proliferative diabetic retinopathy [PDR] sample accessions of uncertain patient independence) providing separate disease context. Frozen cell states were evaluated using within-donor or within-accession summaries of rank scores, pseudobulk burden, detection, and ambient-corrected expression. In GSE294329, cluster 1 met the locked UCell discovery rule, but total ECM7 burden and detection were lower than in other myeloid cells in all five donors. Exact seven-gene replication was unevaluable because CCN2/CTGF was absent from the processed SCP2582 features; no eligible state passed the amended six-gene gate. In PDR, no myeloid state passed the direct-ECM gate, whereas stromal cells had higher ECM7 burden and detection in all five accessions. Secondary SAM-like states occurred in PDR clusters 1 and 6 only and did not rescue the direct-ECM result. An exploratory fixed-cell SCP2582 sensitivity analysis changed cluster membership, but no alternative donor-eligible cluster met the necessary raw gene-coherence criterion. An independently defined collagen-panel sensitivity yielded different patterns in the two PVR cohorts. These data do not demonstrate a reproducible ECM7-expressing myeloid state under the specified rules; small biological cohorts, a restricted gene panel, and indirect use of outcome genes during ambient correction limit broader exclusion.

## 1. Introduction

Proliferative vitreoretinopathy (PVR) is a fibrocontractile response to retinal detachment or ocular injury. Human membranes contain a collagen-rich matrix, retinal pigment epithelial (RPE) and glial cells, myofibroblast-like cells, and macrophages [1,2]. Experimental lineage tracing also implicates RPE as a source of PVR-associated fibroblasts [3]. These observations do not establish which cells synthesise matrix in human tissue.

Macrophages could promote fibrosis through growth-factor signalling, recruitment and activation of stromal cells, or matrix remodelling without producing structural collagen themselves. Co-expression of macrophage and myofibroblast markers in PVR and proliferative diabetic retinopathy (PDR) membranes has prompted proposals of macrophage-to-myofibroblast transition [2,4], but co-expression cannot establish ancestry or distinguish matrix synthesis from ambient RNA, closely apposed cells, and doublets. Scar-associated macrophage states described in liver and lung can interact with collagen-producing mesenchymal cells [5,6]; a GPNMB-positive microglial population has also been reported in PDR [7]. Such programmes can accompany injury without being specific to fibrosis [8,9].

The distinction is relevant to the interpretation of single-cell data from fibrotic tissue. A previous reanalysis of the PDR dataset GSE165784 localised several ECM transcripts predominantly to stromal cells, followed by a response from the original investigators [10,11]. Ambient-RNA correction [12], doublet detection [13], and donor-level rather than cell-level inference [14,15] are therefore necessary safeguards, although each has limitations. In particular, a favourable within-cell rank score need not imply a high absolute burden of matrix transcripts.

We used two independent PVR datasets for discovery and replication and a PDR dataset as a separate localisation and contextual analysis [7,16,17]. Our hypothesis was that, if a reproducible direct matrix-associated transcriptional programme exists in a myeloid state within the sampled PVR membranes, a frozen discovery state would show donor-consistent support beyond a rank score and an eligible state in the independent PVR cohort would show concordant multigene expression and detection. This operational test concerns a seven-gene panel; it cannot establish protein secretion or exclude other profibrotic functions.

The seven primary targets were COL1A1, COL1A2 and COL5A1, which encode structural fibrillar collagens, together with the matrix-associated LUM, THBS2, FBLN2 and CCN2/CTGF. The latter genes broadened the test of a coherent matrix-associated state but are not interchangeable with evidence of structural-collagen synthesis. FN1 and SPARC were secondary markers. The GPNMB, SPP1, LIPA and FABP5 scar-associated macrophage-like (SAM-like) panel was evaluated only as secondary PDR context. The frozen endpoint hierarchy did not permit any secondary marker or programme to rescue a failed primary gate.

## 2. Materials and Methods

### 2.1. Study Design and Analytical Philosophy

**Figure 1:**
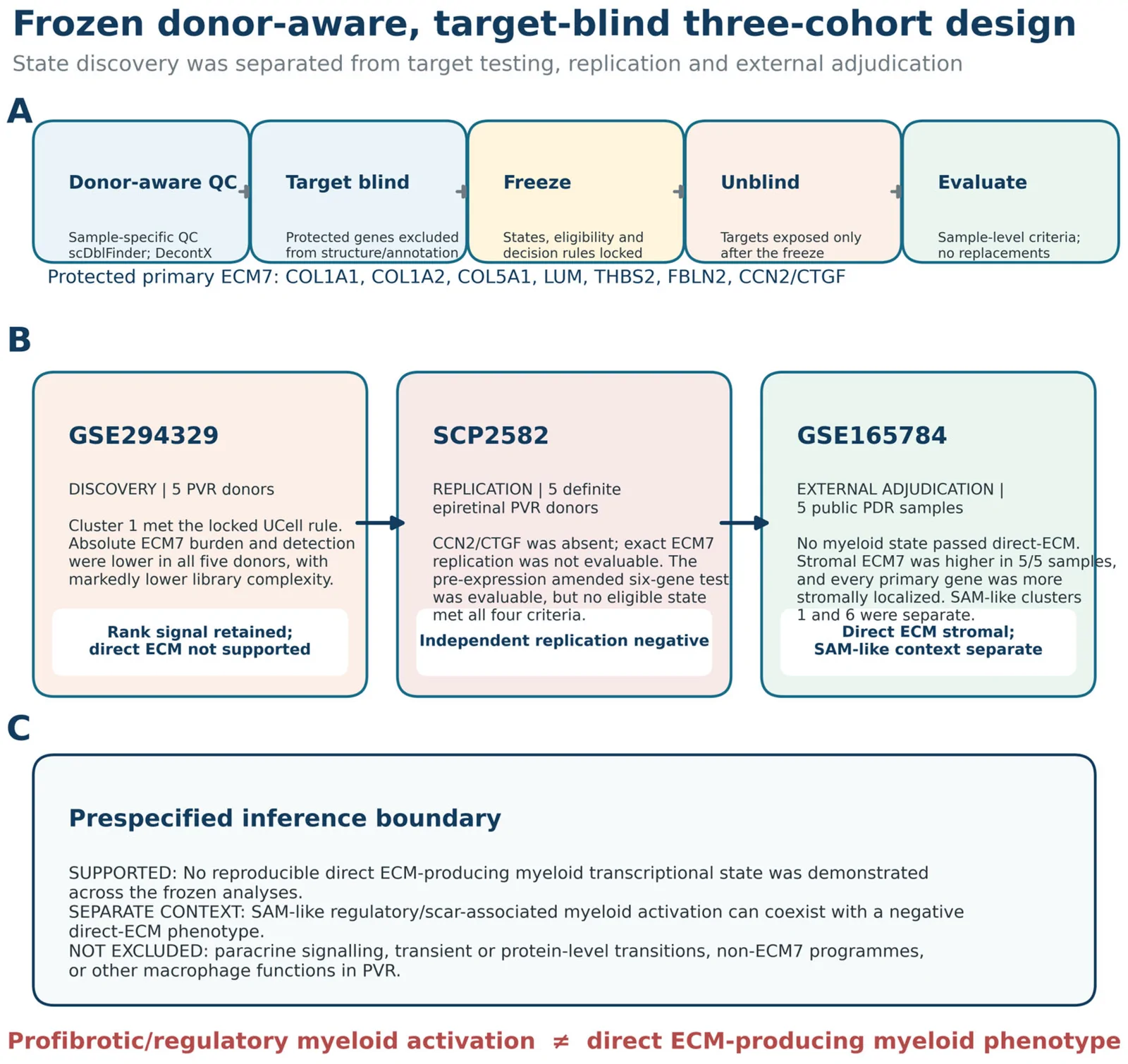
Frozen donor-aware, target-blind three-cohort design. (**A**) Donor- or sample-specific quality control, scDblFinder doublet detection and DecontX ambient-RNA correction preceded target-blind integration, clustering and annotation. The seven protected primary ECM slots (COL1A1, COL1A2, COL5A1, LUM, THBS2, FBLN2 and CCN2/CTGF) were excluded from target-blind structure and annotation. Cell states, eligibility and decision rules were frozen before target unblinding; failed states were not replaced. (**B**) GSE294329 served as discovery in five PVR donors, SCP2582 as independent replication in five definite epiretinal PVR donors, and GSE165784 as external PDR context across five public PDR samples. The locked GSE294329 UCell rule was met, but absolute-expression adjudication did not support direct-ECM production; SCP2582 replication was negative; and GSE165784 localised dominant ECM transcription to stromal rather than myeloid cells while identifying separate SAM-like myeloid states. (**C**) The inference boundary distinguishes regulatory/scar-associated myeloid activation from a direct ECM-producing phenotype and does not exclude paracrine signalling, transient or protein-level transitions, programmes outside ECM7, or other macrophage functions in PVR. The protected genes were present in the full-matrix DecontX correction upstream of the displayed structure; target blindness therefore applies to direct feature and annotation selection, not to every possible indirect effect through ambient correction.

This computational study used a staged, three-cohort design to assess the reproducibility and artefact robustness of a prespecified ECM-producing myeloid transcriptional phenotype in human vitreoretinal membranes. GSE294329 served as the discovery cohort, SCP2582 as the independent human PVR replication cohort, and GSE165784 as an external PDR localisation and contextual cohort. The biological replicate was the donor in the two PVR cohorts. Because of unresolved repository metadata in GSE165784, the consistency unit in that cohort was the public PDR sample accession rather than an asserted independent donor. The overall analytical design is shown in Figure 1, and cohort characteristics are summarised in Table 1 [7,16,17]. The number of biological units was determined by public datasets with suitable membrane anatomy and metadata; no formal power calculation was performed for this exploratory study.

Broad cell types and myeloid states were defined without directly inspecting the protected outcome genes. Cell membership, eligibility and decision rules were recorded before target-gene expression was examined. Because ambient correction used all genes, an indirect influence on state definition remains possible (Section 2.3). The recorded sequence has no independent, public time stamp. No state could be replaced after unblinding, and secondary panels could not rescue a negative primary endpoint.

The primary outcome was a seven-slot ECM programme comprising COL1A1, COL1A2, COL5A1, LUM, THBS2, FBLN2, and CCN2 (with CTGF treated only as the legacy alias for the CCN2 slot). FN1 and SPARC were secondary ECM markers. GPNMB, SPP1, LIPA, and FABP5 formed a contextual scar-associated macrophage-like (SAM-like) panel used only in GSE165784. The SAM-like analysis addressed regulatory or scar-associated activation and was kept logically separate from direct ECM production.

### 2.2. Dataset Identification and Cohort Roles

SCP2582 included eight labelled specimens. The primary replication cohort comprised the five definite epiretinal PVR donors selected before target-gene expression was examined. Of the other three, one had unresolved tissue provenance, one was subretinal, and one was from the ciliary body; they were excluded from the primary analysis and retained for sensitivity assessments. Cohort eligibility and quality control are detailed in Tables S1 and S2.

GSE165784 contains one PVR sample and five PDR samples. The single PVR sample was excluded because it could not support replicate-level inference. Although five PDR samples are deposited, the source study describes three PDR patients and does not provide a reliable sample-to-patient crosswalk. We therefore report consistency across five PDR samples without treating them as five independent patients (Table S1).

### 2.3. Target-Gene Handling and Analysis Locking

Outcome genes were excluded from doublet-detection input, variable-feature selection, integration and clustering features, marker testing and lineage annotation. Standard cell-quality measures used all genes. Primary ECM genes and secondary FN1 and SPARC were protected in both PVR cohorts; the PDR analysis also protected the secondary GPNMB, SPP1, LIPA and FABP5 panel.

DecontX ambient-RNA correction was performed on all measured genes, including protected outcomes. Corrected non-target expression and contamination estimates subsequently informed cell-state definition. The protected genes therefore could not directly select features or define markers, but they could influence clustering indirectly through ambient correction. This limitation is assessed separately in the exploratory sensitivity analysis (Section 2.15).

Cell-state definitions and eligibility were recorded before target-gene expression was examined, and the recorded state was checked before outcome evaluation. The documented sequence supports the reported order of analysis but does not establish an independently witnessed or publicly time-stamped freeze. Figure 1 and Table S3 summarise the sequence; archive limitations are described in Section 2.16.

### 2.4. Data Preprocessing and Donor/Sample-Specific Quality Control

#### 2.4.1. Input Reconstruction and Feature Naming

For GSE294329, donor-specific count data and gene annotations were checked for consistent dimensions and harmonised by gene symbol before analysis. Duplicate symbols were distinguished consistently, and donor identifiers were retained to support within-donor comparisons.

For SCP2582, the five epiretinal donor specimens were identified by checking tissue and donor annotations in the deposited data. Donor-specific counts were then used for quality control and subsequent within-donor comparisons.

For GSE165784, count data from the five PDR samples were harmonised across the deposited formats and subjected to the same sample-specific quality-control procedure. Sample characteristics are reported in Table S1.

#### 2.4.2. Robust Donor/Sample-Specific QC

Quality-control thresholds were calculated independently within each donor or public sample. For each cell, log10(nFeature_RNA + 1) and log10(nCount_RNA + 1) were computed. A robust dispersion estimate used the median absolute deviation with scaling constant 1.4826; if non-finite or zero, IQR/1.349 was used, followed by a value of 0.01 if required. Lower bounds were the larger of the prespecified floor and median minus 3 robust deviations; upper bounds were median plus 4 robust deviations. Cells were retained if they had at least 300 detected features, at least 500 RNA counts, mitochondrial transcript fraction at most 20%, and both log-scale metrics within the donor/sample-specific bounds.

The number of input cells, cells retained after basic QC, predicted doublets, singlets retained, and median estimated contamination were recorded for each donor or sample. QC decisions were made before target expression was inspected. Donor- and sample-specific QC records are reported in Table S2.

### 2.5. Doublet Detection and Ambient-RNA Correction

scDblFinder [13] was applied within each donor or PDR sample after basic quality control, with protected outcome genes excluded from the doublet-detection input. Cells classified as doublets were removed. In SCP2582, this was a residual doublet screen because the deposited data had already undergone source-study processing.

DecontX [12] was applied separately to each donor or public-sample singlet matrix with all genes and recorded default arguments. The full-matrix input is important: protected genes could influence its inferred contamination model even though they were excluded from subsequent integration features and marker testing. Corrected counts and contamination estimates were retained in a DecontX assay. Unrounded corrected counts informed target-blind normalisation, integration and the locked GSE294329 UCell analysis. Adjudication also used original raw counts and integer-rounded-DecontX counts to test sensitivity to fractional residuals. Summary QC outputs appear in Table S2.

### 2.6. Target-Blind Integration, Clustering, and Cell-Type Annotation

Within each cohort, cells were normalised after ambient correction using Seurat [18], and protected genes were excluded from variable-feature selection. Integration used 3000 variable genes per donor or sample and 3000 integration features, with principal components 1–30 for anchor finding. The integration features were scaled, and 50 principal components were computed; neighbour graphs and UMAP used principal components 1–30. Clustering was evaluated at resolutions 0.4, 0.6 and 0.8; resolution 0.6 was the prespecified primary choice. GSE165784 integration used k.weight = 50; remaining settings followed recorded software defaults.

Positive cluster markers were identified using non-protected genes, a minimum detection fraction of 0.10 and a log-fold-change threshold of 0.25. Broad lineage annotation was reviewed against target-independent markers, established lineage panels, cell-state structure and donor/sample representation. The deposited SCP2582 author annotation was used only as a descriptive cross-check. Every compartment required a reviewed lineage annotation before inclusion.

Lineage panels covered myeloid, lymphoid, stromal, retinal pigment epithelial, endothelial, epithelial and glial populations. Clusters with unresolved mixed or cycling identity were not admitted to the primary high-confidence myeloid populations. Annotations and marker summaries are documented in Figure S1 and Table S3.

### 2.7. Definition and Freezing of Myeloid States

Within-myeloid states were defined using non-protected genes and positive markers meeting a minimum detection fraction of 0.10 and a log-fold-change threshold of 0.25. Cluster assignments, donor or sample representation and eligibility were recorded before outcome evaluation. The prespecified myeloid compartments and presence rules are summarised in Figure 1 and Table S3.

### 2.8. GSE294329 Discovery Analysis

Each primary ECM gene was required to map uniquely in the discovery dataset; CCN2 was preferred, with CTGF accepted only as its former symbol when CCN2 was absent. The seven-gene programme was scored using unrounded ambient-corrected expression and UCell [19] with maxRank = 1500. Donors with at least 50 frozen myeloid cells were informative. Within each donor, the effect was the difference in mean UCell score between a state and all other myeloid cells.

**Figure 2:**
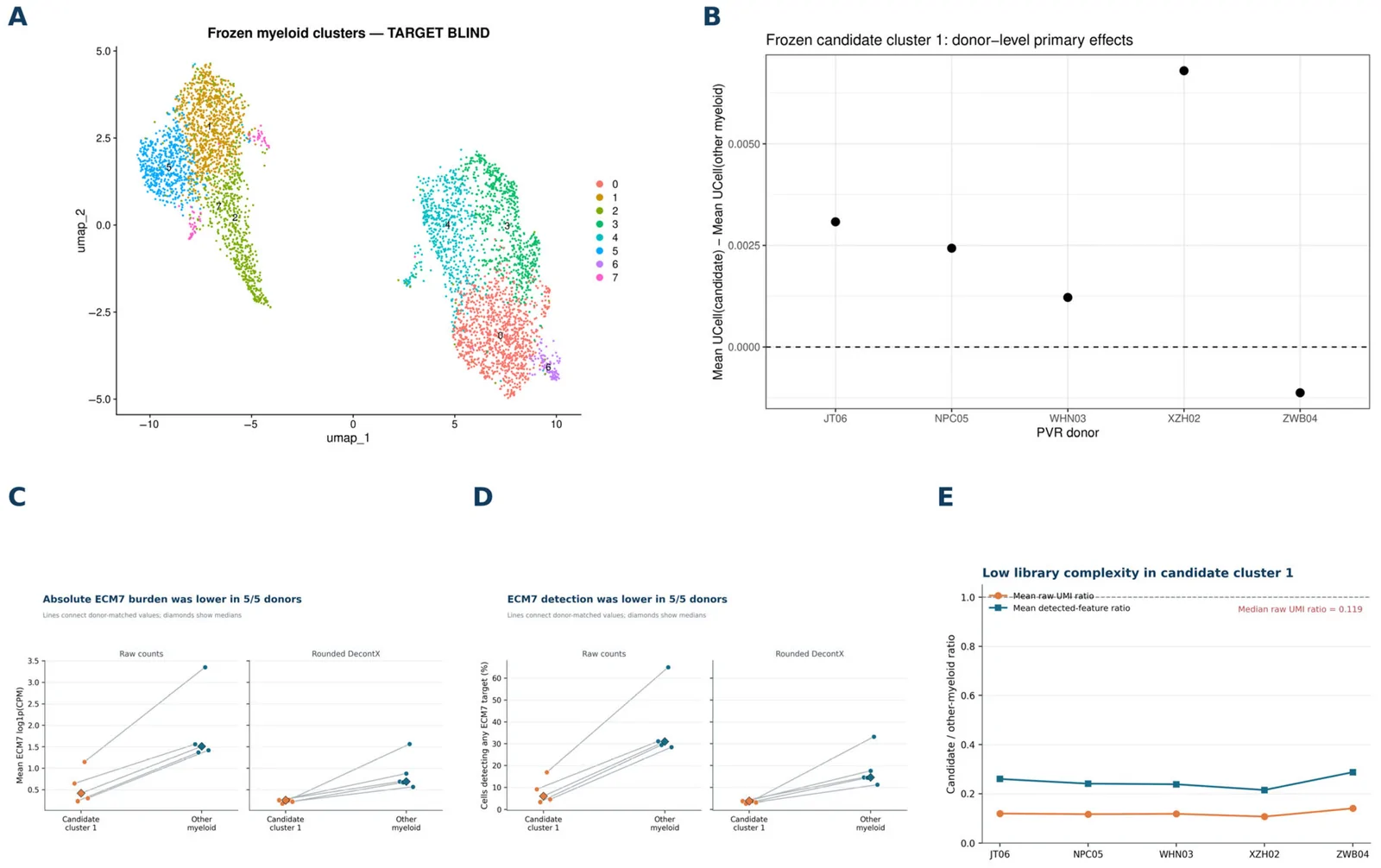
A locked rank-based discovery signal in GSE294329 was not supported by absolute ECM expression. (**A**) Target-blind UMAP of 4564 high-confidence myeloid cells from five PVR donors, coloured by eight frozen subclusters. Cluster 1 was nominated only after target unblinding. (**B**) Within-donor difference in mean ECM7 UCell score between cluster 1 and the remaining myeloid cells. Four of five donors had a positive difference (median difference, 0.002432), satisfying the prospective discovery-support rule; the secondary one-sided exact sign test yielded *p* = 0.1875. In panel (**B**), the dashed horizontal line indicates zero difference in mean UCell score. In panel (**E**), the dashed line indicates a candidate-to-other-myeloid ratio of 1. (**C**) Donor-matched mean ECM7 log1p-CPM burdens in cluster 1 and other myeloid cells were derived separately from the raw and integer-rounded-DecontX counts. Burden was lower in cluster 1 in all five donors under both representations. (**D**) Corresponding fractions of cells detecting any ECM7 target were also lower in cluster 1 in all five donors. Lines connect donor-matched values, and diamonds denote medians. (**E**) Candidate-to-other-myeloid ratios for mean raw UMI count and detected-feature count; the median UMI ratio was 0.11934. Panels (**A**,**B**) retain the prespecified discovery record, whereas panels (**C**–**E**) are post-unblinding adjudication analyses. The lower expression magnitude, lower detection and markedly lower library complexity precluded a direct ECM-producing interpretation.

Among clusters satisfying the frozen presence rule, the discovery candidate was the cluster with the largest median donor-level mean-score difference; a tie was resolved by cluster identifier. Discovery support required a positive median donor-level difference and a positive difference in at least four informative donors. No cell-level *p*-value was used. A one-sided exact binomial sign test among non-zero donor effects was reported only as a secondary directional descriptor. FN1 and SPARC were evaluated descriptively and could not select or rescue a candidate. The locked discovery structure and result are shown in Figure 2A,B and detailed in Table S4.

### 2.9. Post-Unblinding Artefact Adjudication in GSE294329

The absolute-expression and technical assessment was specified after the initial discovery analysis; it is interpreted as adjudication rather than as another confirmatory test. The nominated state could not be replaced, and the gene panel could not be changed.

- Rank-score robustness: The candidate-versus-other donor effects were recomputed using raw counts and rounded-DecontX counts at maxRank values 500, 1000, and 1500, in addition to the locked unrounded-DecontX analysis.
- Leave-one-gene-out robustness: Each primary slot was omitted in turn, and six-gene UCell scores were calculated from raw and rounded-DecontX counts at maxRank = 1500.
- Absolute expression: Per-gene raw and rounded-DecontX detection, mean counts per cell, the summed seven-gene burden, the fraction of cells with any primary target detected, and donor-level pseudobulk expression were compared between the fixed candidate and other myeloid cells.
- Pseudobulk scaling: CPM was the summed target counts divided by total library size within each donor and group, multiplied by 10^6^. For log-scale displays, log2(CPM + 0.1) was calculated after CPM; an earlier pseudocount-placement error was corrected before interpretation.
- Fractional residual audit: Raw detection, any positive DecontX value, rounded-DecontX detection, fractional-only values, values > 10^−6^, and values ≥ 0.5 were summarised by target, donor, and group.
- Technical and identity checks: Raw UMI and detected-feature complexity, DecontX contamination, scDblFinder score, and fixed target-independent myeloid, stromal, RPE, and photoreceptor panel burdens were evaluated donor by donor.
- Synthetic mixtures: In each donor, 300 myeloid–myeloid and 300 myeloid–stromal cell pairs were resampled with replacement. Normalised lineage-marker burdens in these mixtures were compared with those in the observed candidate, other myeloid cells and stromal cells.

The post-unblinding adjudication asked whether the locked rank-based discovery signal remained directionally coherent in absolute-expression and artefact-aware representations. These analyses could qualify the interpretation of the discovery result but could not modify the original decision record. The adjudication outputs are summarised in Figure 2C–E and Figures S2 and S3, and Table S4.

### 2.10. SCP2582 Replication Under an Amended Six-Gene Assay

The frozen SCP2582 eligible-cluster set comprised clusters 0–4. Unlike discovery, replication did not select a state by UCell score or effect size: every eligible frozen cluster was tested against the same multicomponent rule. State effects were calculated within donor relative to all other frozen high-confidence myeloid cells from that donor.

A feature-name diagnostic performed before SCP2582 target-expression analysis showed that CCN2 and CTGF were absent from the frozen RNA feature set, whereas COL1A1, COL1A2, COL5A1, LUM, THBS2, and FBLN2 were uniquely present. Exact seven-gene replication was therefore not evaluable. A documented protocol amendment, made before examining target-expression values, evaluated the six assayable prespecified genes without adding a substitute. The absolute individual-gene-coherence threshold remained at least three genes.

A frozen state passed the amended primary replication gate only if all four conditions were met:

1. Summed raw six-gene pseudobulk CPM was higher in the state than in other myeloid cells in at least four of five donors.
2. The raw fraction of cells with at least one assayable primary target detected was higher in at least four of five donors.
3. At least three of the six assayable genes each showed both higher raw pseudobulk CPM and higher raw detection in at least four of five donors.
4. Summed rounded-DecontX six-gene pseudobulk CPM was higher in at least four of five donors.

**Figure 3:**
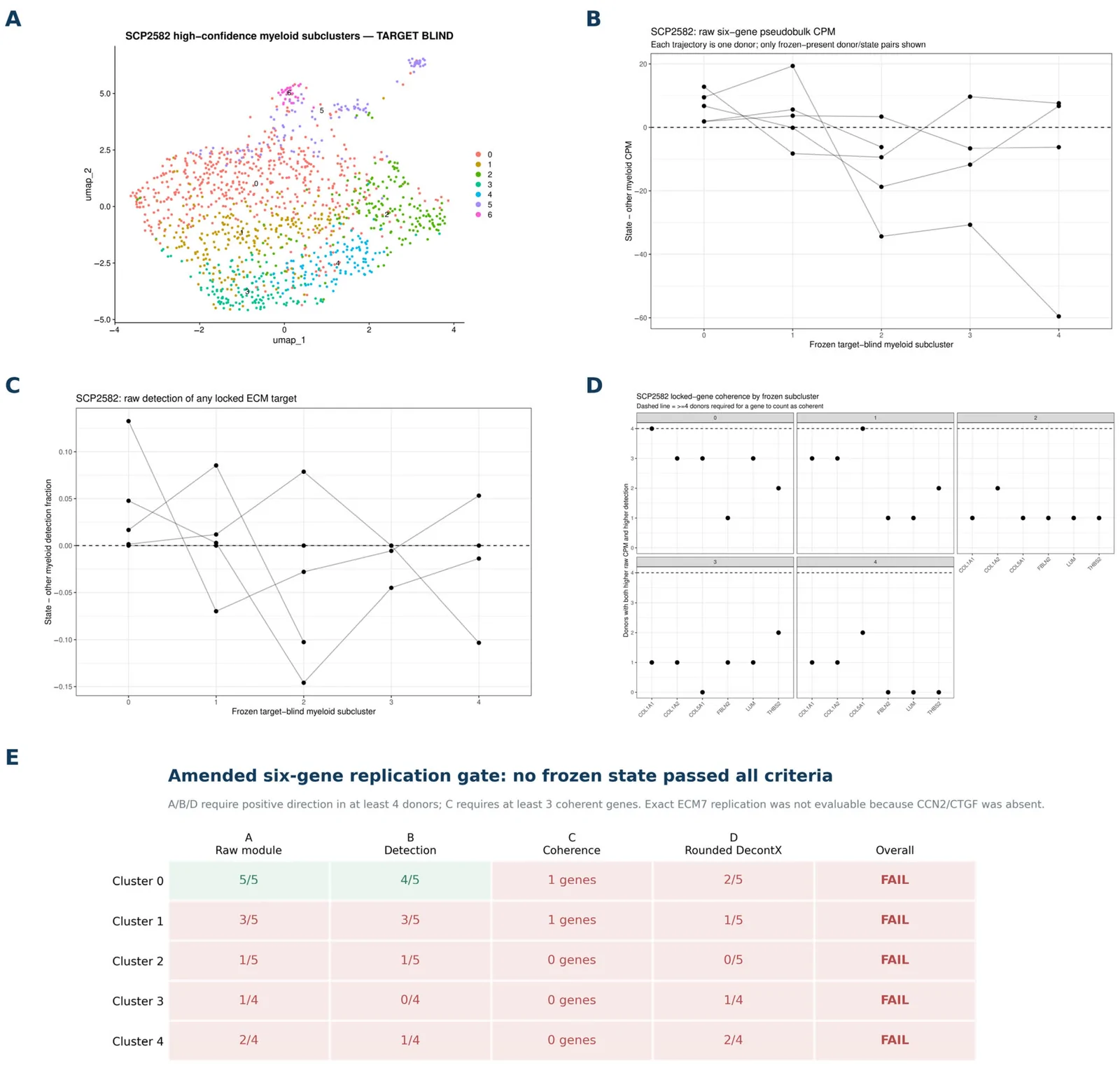
Target-blind myeloid-state definition and amended six-gene replication in the independent SCP2582 cohort. (**A**) UMAP of 1281 high-confidence myeloid cells coloured by frozen target-blind subcluster. Seven subclusters were frozen; clusters 0–4 met the locked cross-donor-presence criterion and were eligible for replication. (**B**) Donor-level difference in raw summed pseudobulk CPM for the six assayable prespecified ECM genes between each eligible state and the remaining myeloid cells from the same donor. Each trajectory represents one donor, and positive values indicate higher state expression. (**C**) Corresponding donor-level difference in the fraction of cells detecting at least one assayable ECM target. (**D**) Number of donors in which each gene showed both higher raw CPM and higher detection in the state; the dashed line marks the four-donor gene-coherence requirement. (**E**) Locked decision grid for raw module abundance, raw detection, gene coherence and rounded-DecontX module abundance. Cluster 0 met the two raw criteria but not coherence or rounded-DecontX criteria; no eligible state met all four conditions. CCN2/CTGF was absent from the processed feature set, so exact seven-gene replication was not evaluable. The six assayable prespecified genes were evaluable under the amendment locked before target-expression values were examined, and the amended replication was negative. In panel (**E**), green cells indicate individual criteria that were satisfied, whereas red cells indicate criteria that were not satisfied. The final column reports whether each frozen state passed the complete four-component replication gate.

Failure of every eligible state to satisfy all four components defined a negative result in this independent PVR cohort under the amended six-gene assay. FN1/SPARC, library complexity, DecontX contamination, scDblFinder score, target-independent lineage burdens, and raw/rounded-DecontX UCell scores (maxRank = 1500) were secondary. Synthetic myeloid–stromal mixtures were prospectively conditional on at least one state passing the primary gate; a negative primary gate did not trigger that analysis. The independent replication analysis is summarised in Figure 3 and Table S5. Target-blind broad annotation and frozen myeloid provenance for SCP2582 are shown in Figures S4 and S5.

### 2.11. GSE165784 PDR Contextual and Localisation Analysis

The exact seven-slot ECM programme and the four SAM-like genes were structurally available in the frozen GSE165784 objects. All seven eligible frozen myeloid subclusters (0–6) were evaluated; no cluster was selected by effect magnitude. For each public PDR sample, each state was compared with all other frozen primary myeloid cells from that sample. A state passed the primary direct-ECM gate only if all four criteria used in the replication framework were met: higher raw summed ECM7 CPM in at least four samples; higher raw any-target detection in at least four samples; at least three of seven genes each showing both higher raw CPM and higher raw detection in at least four samples; and higher rounded-DecontX summed ECM7 CPM in at least four samples.

**Figure 4:**
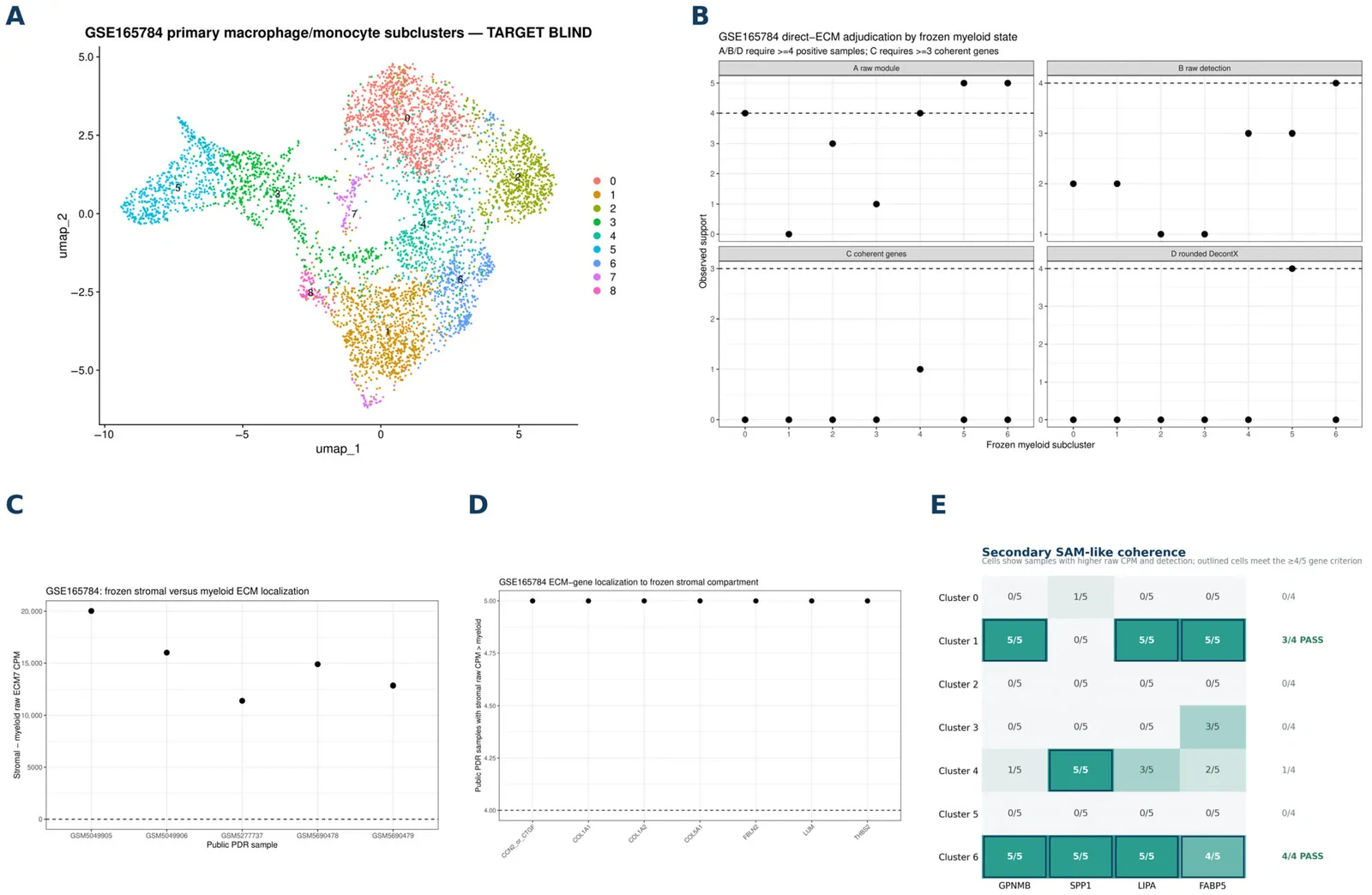
PDR contextual analysis in GSE165784 separates stromal ECM localisation from SAM-like myeloid activation. (**A**) Target-blind UMAP of 5105 frozen primary macrophage/monocyte cells from five public PDR sample accessions, partitioned into nine subclusters before target unblinding. Clusters 0–6 were eligible. (**B**) Corrected direct-ECM gate. Points show the number of samples with higher raw ECM7 CPM (**A**), higher raw any-target detection (**B**), the number of coherent primary genes (**C**), and higher rounded-DecontX ECM7 CPM (**D**). Dashed lines mark the locked thresholds: four samples for A, B and D, and three coherent genes for C. No cluster met all four criteria. (**C**) Stromal-minus-myeloid raw ECM7 CPM was positive in all five public samples; raw detection and rounded-DecontX CPM had the same 5/5 direction. (**D**) Every primary ECM gene had higher stromal than myeloid raw CPM in all five samples; raw detection and rounded-DecontX CPM showed the same direction. (**E**) Secondary SAM-like coherence. Cells give the number of samples in which both raw CPM and detection were higher than in the remaining myeloid cells; outlined cells meet the four-sample gene criterion. Cluster 1 was coherent for GPNMB, LIPA and FABP5 but not SPP1, whereas cluster 6 was coherent for all four genes. This contextual result did not alter the negative direct-ECM decision. Panel (**B**) uses the corrected gate with the criterion-C threshold at three coherent genes. Results are framed across five public PDR samples without asserting five independent donors. In panel (**E**), increasing green intensity represents a greater number of public PDR samples showing concordantly higher raw CPM and detection for the corresponding gene. Outlined cells meet the four-sample gene-coherence requirement. A cluster satisfies the contextual SAM-like criterion when at least three of the four genes are coherent.

The five GSM accessions were treated as consistency units. No cell-level or unqualified donor-level inference was made, and no myeloid state, signature, or threshold could be changed after unblinding. FN1 and SPARC were secondary and could not rescue a negative direct-ECM endpoint. The external gate is shown in Figure 4A,B, with complete results in Table S6. Target-blind broad annotation and frozen myeloid provenance are shown in Figures S6 and S7; detailed direct-ECM effects appear in Figure S8.

### 2.12. Stromal Localisation Analysis

Before target unblinding, the GSE165784 stromal comparator was frozen from reviewed broad clusters 6 and 7, whereas the primary macrophage/monocyte compartment was frozen from broad clusters 0–5 and 12. Within each public PDR sample, all frozen stromal cells were compared with all frozen primary myeloid cells. The prespecified summaries were summed ECM7 raw CPM, fraction of cells with any ECM7 target detected, summed ECM7 rounded-DecontX CPM, and the corresponding raw CPM, detection, and rounded-DecontX CPM for each individual primary gene.

The localisation analysis was descriptive and sample-matched. Its purpose was to determine whether the dominant ECM transcriptional burden localised preferentially to the frozen stromal compartment rather than to identify a new cell state. Sample-matched synthetic myeloid–myeloid and myeloid–stromal mixtures were also generated for every eligible myeloid state/sample pair using 300 resampled pairs per mixture class and the same summed-count, summed-library normalisation used in GSE294329. Stromal localisation is summarised in Figure 4C,D and Table S7.

### 2.13. Secondary SAM-like Analysis

The SAM-like panel was evaluated only in GSE165784 and only after the myeloid structure had been frozen. For each eligible myeloid cluster, each of GPNMB, SPP1, LIPA, and FABP5 was considered coherent if both raw pseudobulk CPM and raw detection were higher than in other myeloid cells in at least four public PDR samples in which the state met the locked presence rule. A cluster satisfied the contextual SAM-like criterion if at least three of the four genes were coherent.

This secondary criterion was interpreted as evidence of regulatory or scar-associated myeloid activation. It was not a direct-ECM criterion and could not rescue a negative primary ECM phenotype. The outcome was reported gene by gene so that incomplete coherence, including discordant SPP1 behaviour, remained visible. The contextual SAM-like analysis is shown in Figure 4E and detailed in Table S8.

### 2.14. Statistical Analysis

The donor was the biological unit in each PVR cohort (five donors each). For GSE165784, the unit of descriptive consistency was one public PDR sample accession; five accessions cannot be assumed to represent five independent patients. Cells contributed only to within-unit summaries for the ECM endpoints. Each frozen state was compared with other myeloid cells from the same unit, or stromal cells with myeloid cells from the same PDR accession. No cell-level *p*-value was used to establish discovery, replication or contextual support.

Continuous summaries comprised UCell scores, raw or corrected pseudobulk counts per million (CPM), detection fractions, RNA counts, detected-feature counts, doublet scores and contamination fractions. Pseudobulk CPM was the target-count sum divided by the total RNA-count sum in the donor/sample group, multiplied by 10^6^; detection was the proportion of cells with a positive count. Paired state-minus-comparator effects were summarised by their median, range and number of units with positive direction. Library-size and feature-count QC used within-unit median/robust MAD bounds on log10 scales, with IQR fallback, rather than a normal-distribution assumption. No categorical group comparison or normality-based *t*-test formed part of the endpoint analysis.

The prespecified decisions were discrete direction and multigene-coherence gates, not tests at an α threshold. No α level for the primary gate was prespecified, and none is retrospectively imposed. A one-sided exact binomial sign test for the five-donor-level discovery effects was secondary and descriptive (four positive, *p* = 0.1875). Seurat FindAllMarkers used its default cell-level Wilcoxon rank-sum test, only positive markers, minimum detection fraction 0.10 and log-fold-change threshold 0.25; its *p*-values aided target-blind annotation and cannot be treated as donor-level ECM inference. For an illustrative single fixed state with independent, exchangeable donor directions, a four-of-five directional rule has an 80% chance of being met only if the probability of a positive effect in each donor is approximately 0.831; if that probability is 0.8, the chance is 0.737, and if it is 0.6, the chance is 0.337. There is no minimum continuous effect magnitude in this directional rule. The nominal exact 95% binomial interval for four positive directions in five donors is 0.284–0.995; because the discovery cluster was selected by its score and the endpoint combines other criteria, this interval is descriptive and does not supply coverage for the selected composite result. Rare or heterogeneous states may therefore be missed. Patient-level confidence intervals are not calculated from the PDR accessions because patient identity is unresolved. The implementation and correction history is documented in Table S9.

### 2.15. Sensitivity and Adjudication Analyses

Sensitivity assessments using the frozen cell states and prespecified outcome genes did not replace a primary decision. Exploratory analyses addressed structural-collagen expression, myeloid/stromal identity, ambient-correction effects and rank-score behaviour (Tables S10–S14; Figure S9). They are reported separately from the prespecified endpoint.

In an exploratory SCP2582 sensitivity analysis, DecontX was refitted within each donor after excluding eight protected genes. The original full-gene correction was reproduced in one donor as a control. The same 1281 previously selected myeloid cells were reclustered with the original settings, and state membership was compared after matching cluster labels. We then reapplied the original within-donor raw six-gene abundance, detection and gene-coherence criteria to the alternative states. State eligibility still required at least 10 cells and 1% of a donor’s myeloid compartment. This analysis did not reassess upstream myeloid lineage selection or the corrected-gene criterion, which could not be measured from the target-excluded correction. This analysis was outside the prespecified endpoint assessment and does not alter the frozen decision (Table S12).

A separate exploratory SCP2582 sensitivity analysis repeated myeloid feature selection and clustering in the same 1281 cells while allowing protected genes into these steps. An original-workflow control recovered the archived state labels and raw replication counts. Alternative clusters were assessed only against the original within-donor raw six-gene criteria; the corrected-gene criterion, broad lineage selection and other cohorts were not reassessed. This outcome-informed sensitivity cannot replace the frozen replication test (Table S13).

A separate exploratory GSE294329 diagnostic used the archived frozen myeloid cells and labels to verify the five locked unrounded-DecontX donor UCell effects to numerical precision. For cluster 1 versus other myeloid cells within each donor, we then described raw RNA complexity, primary-gene detection, positive-expression rank proxies capped at 1501 for maxRank = 1500, and UCell scores on raw, unrounded-DecontX and integer-rounded-DecontX matrices. Positive cellwise library-size scaling and log1p CPM preserve within-cell gene ranks; DecontX changes and rounding can change them. A two-cell illustrative calculation held target counts fixed and changed only the abundance of higher-ranked background genes. These comparisons are diagnostic and cannot identify a unique causal source of the frozen rank result (Table S14).

To investigate the missing CCN2/CTGF feature, we compared gene identifiers and feature annotations in the original processed SCP2582 dataset with a donor-specific dataset retained after quality control. No target-expression values were examined. This identifies when the feature was already absent but cannot distinguish platform design, annotation, or earlier preprocessing as the cause (Table S15).

A further exploratory sensitivity analysis used the published human NABA_COLLAGENS set of 44 genes (MSigDB M3005) [20], evaluated both in full and after excluding the three structural collagens in the original ECM7 panel (COL1A1, COL1A2 and COL5A1). The latter externally sourced panel contained 41 genes; 41 mapped uniquely in the frozen GSE294329 myeloid object and 40 in SCP2582 because COL6A5 was unavailable. COL6A5 had zero raw CPM in every GSE294329 candidate and comparator donor group. Original frozen myeloid cells, donor labels and state eligibility were retained. We compared GSE294329 candidate cluster 1 and all five previously eligible SCP2582 clusters with the remaining myeloid cells from the same donor, reporting raw pseudobulk summed collagen CPM, the fraction detecting any assayable collagen, and integer-rounded-DecontX pseudobulk CPM. The original GSE294329 ECM7 raw-detection directions and SCP2582 six-gene A/B counts were independently reproduced as controls. These exploratory comparisons had no new selection threshold or replication decision and did not analyse the external PDR cohort (Table S16).

### 2.16. Reproducibility, Software, and Computational Environment

Analyses were performed using R version 4.6.1 (R Foundation for Statistical Computing, Vienna, Austria), with Seurat 5.5.1, scDblFinder 1.26.7, celda 1.28.0 (DecontX) and UCell 2.16.0, among other packages. Cohort-level random seeds were 294329, 2582 and 165784. Full software versions and implementation corrections are documented in Table S9.

Publicly accessible GEO inputs were downloaded again and matched to the original input records, confirming their identity but not the timing of analytical decisions. Implementation corrections, including the CPM display calculation and the figure label for gene coherence, were documented and did not change the frozen cell populations, thresholds or outcome decisions (Table S9).

The restricted analysis archive includes code, available outputs and records of the cell-state and outcome decisions. Some large derived analysis objects are absent from the deposited package, so it does not currently permit an exact rerun from that package alone. Its internal records document the analytical sequence but cannot independently establish the date of the freeze.

## 3. Results

The Results are organised in the order fixed by the analytical design: target-blind cohort freezes, GSE294329 discovery, post-unblinding artefact adjudication, independent SCP2582 replication, and external GSE165784 adjudication. Secondary markers are reported separately from the primary direct-ECM endpoint.

### 3.1. Target-Blind Freezes Established Donor- and Sample-Represented Myeloid Compartments

In GSE294329, donor-specific quality control and doublet removal retained 23,065 of 29,813 input barcodes. Target-blind annotation identified 4564 high-confidence myeloid cells across all five PVR donors. Eight within-myeloid clusters were frozen, of which six (clusters 0–5) met the locked donor-presence requirement for discovery (Figures S1 and S2; Table 1 and Tables S2 and S3).

In SCP2582, 3421 cells remained after donor-specific quality control and residual doublet removal from the 3738 cells in the public object. The intersection-based primary definition contained 1281 high-confidence myeloid cells from the five definite epiretinal PVR donors. Seven states were frozen, and clusters 0–4 were eligible for independent replication (Figures S4 and S5; Table 1 and Tables S2 and S3).

Across the five public GSE165784 PDR samples, 6817 of 8011 deposited cells remained after sample-specific quality control and doublet removal. The target-blind freeze contained 5105 primary macrophage/monocyte cells and a separate 548-cell stromal reference. Nine myeloid subclusters were frozen, of which seven (clusters 0–6) met the locked sample-presence rule (Figures S6 and S7; Table 1 and Tables S2 and S3).

The feature-level firewall audit found no protected genes among integration features, target-blind markers or frozen centroids. These checks establish exclusion from direct feature selection and marker-based annotation; they do not rule out an indirect effect of the full-gene DecontX correction on the resulting states (Section 2.3).

### 3.2. The Locked GSE294329 Discovery Rule Nominated Myeloid Cluster 1

After unblinding, the prospectively locked UCell rule selected cluster 1. This state comprised 892 cells and was represented in all five donors (77–262 cells per donor). The median donor-level difference in mean ECM7 UCell score between cluster 1 and the remaining myeloid cells was 0.002432; the difference was positive in four of five donors. Cluster 1 therefore met the locked discovery-support rule. The secondary one-sided exact sign test was not statistically compelling (*p* = 0.1875) and was not the basis of the prospective decision (Figure 2A,B and Figure S2; Table S4).

No alternate state was nominated after the initial discovery result. The positive rank-based result remained recorded and proceeded unchanged to the specified absolute-expression and independent-cohort assessments.

### 3.3. Absolute-Expression and Artefact Adjudication Did Not Support an ECM-Producing Phenotype in the Discovery State

The rank-based discovery result did not translate into greater absolute expression of the full ECM7 programme. Cluster 1 had a lower mean raw seven-gene log1p-CPM burden than other myeloid cells in every donor and a lower fraction of cells with any raw target detected in every donor (0/5 donors higher for both comparisons). For no primary gene was raw detection higher in cluster 1 in any donor. Raw detection was lower in all five donors for CCN2/CTGF, COL1A1, COL1A2, COL5A1, and FBLN2, in four donors for LUM, and in two donors for THBS2, with ties in the remaining comparisons.

The candidate state was markedly less complex than the other myeloid cells. Across donors, its mean raw UMI count was 10.8–14.2% of the comparator mean (median ratio, 0.11934), and its mean detected-feature count was 21.5–28.7% of the comparator mean. This imbalance provides a technical explanation for why a rank-based score could increase despite a lower absolute target burden.

Rank-score sensitivity analyses were also non-uniform. Raw-count UCell comparisons retained the same-direction rule at maxRank values of 500, 1000, and 1500, whereas the rounded-DecontX score at the locked maxRank of 1500 had a negative median donor effect (−0.000642) and was positive in only two donors. Each rounded-DecontX leave-one-gene-out analysis failed the same-direction rule. Corrected CPM-first pseudobulk gene directions were mixed rather than uniformly negative, but they did not establish a broad, detection-supported seven-gene state and did not alter the absolute-burden adjudication.

In the exploratory cellwise diagnostic, the five archived cluster 1 donor UCell contrasts were reproduced to numerical precision. Unrounded-DecontX and raw scores favoured cluster 1 in four of five donors, whereas rounded-DecontX scores did so in only two. Across all five donors, candidate cells had lower median raw UMI and feature counts than their within-donor comparators and lower mean primary-target UMIs. Capped gene-rank and detection summaries varied by donor and matrix. An illustrative calculation with identical target counts gave a UCell score of 0.933 when 100 stronger background genes were present and zero when 1800 were present; it demonstrates a possible rank mechanism, not the cause of the observed cluster differences (Table S14).

Lineage and mixture controls supported a myeloid identity for cluster 1 and did not suggest a dominant stromal-doublet population. Nonetheless, the state lacked the absolute ECM burden needed for a direct ECM-producing interpretation. The positive rank-based discovery result was retained but did not establish a coherent ECM-producing myeloid state (Figure 2C–E and Figures S2 and S3; Table S4).

### 3.4. SCP2582 Did Not Meet the Amended Six-Gene Replication Gate

Feature-resolution auditing showed that CCN2/CTGF was absent from the processed SCP2582 RNA feature set. Exact seven-gene replication was therefore not evaluable. Under the amendment recorded before inspection of SCP2582 target-expression values, the six assayable prespecified genes were tested in every eligible frozen state without UCell-based state selection.

The feature audit found the same 17,417 RNA feature names in the original processed SCP2582 dataset and in a donor-specific dataset after quality control. CCN2 and CTGF were absent from both, whereas the other six primary genes and secondary FN1 and SPARC were present. The available data do not establish whether platform design, annotation or upstream preprocessing explains the absence (Table S15).

No eligible state met all four locked replication conditions. Cluster 0 satisfied the raw module-abundance and raw-detection criteria, but only one gene met the coherence rule, and rounded-DecontX module abundance was higher in only two donors. Every other eligible state failed at least three of the four conditions. The amended six-gene replication was therefore negative.

UCell was run successfully as a secondary descriptor only and was not used to choose a replacement state. The conditional synthetic-mixture analysis was not triggered because no cluster passed the primary A + B + C + D gate. Descriptive FN1 and SPARC signals were likewise not permitted to rescue the negative primary replication result. Descriptively, cluster 2 had higher FN1 and SPARC raw CPM in 5/5 donors and higher detection in 4/5 donors for each marker, but this state failed all four primary six-gene criteria (Figure 3; Table S5).

In an exploratory fixed-cell diagnostic, the original-assay control reproduced all 1281 frozen labels and raw A/B/C gate counts. Excluding protected genes from DecontX changed 437/1281 cluster assignments after label matching (adjusted Rand index, 0.440). All five eligible alternative groups failed raw gene coherence (zero or one coherent gene; three required). One met raw module abundance; none met raw detection. State membership was therefore sensitive to protected-gene inclusion, while the necessary raw-coherence rule remained unmet in these fixed cells. The target-excluded fit cannot evaluate the corrected-target CPM or supply a full four-gate decision (Table S12).

A separate exploratory analysis allowed protected genes into feature selection during fixed-myeloid-cell reclustering. Its original-workflow control again reproduced all 1281 frozen labels and raw A/B/C counts. The alternative selected one protected integration feature (FN1, a secondary marker), rather than none; 1999/2000 integration features were shared. Without label matching, 148/1281 cells changed their numerical cluster label (adjusted Rand index, 0.721). No donor-eligible alternative group met raw gene coherence (the maximum was two of the three required genes). This checks one direct feature-selection route in these fixed myeloid cells; it does not test broad myeloid selection or reproduce the corrected-target CPM gate D (Table S13).

### 3.5. PDR Contextual Analysis Did Not Identify an ECM7-Expressing Myeloid State

All seven primary ECM slots were assayable in GSE165784, with CTGF mapping to the prespecified CCN2/CTGF slot. None of the seven eligible frozen myeloid clusters met the complete A + B + C + D direct-ECM gate. Cluster 6 met the raw module-abundance and raw-detection conditions but had no coherent primary ECM genes and no sample with higher rounded-DecontX module CPM. Cluster 5 met the raw module-abundance and rounded-DecontX conditions but failed raw detection and gene coherence. These partial patterns did not satisfy the locked multicomponent definition.

The secondary ECM markers did not alter this decision. In cluster 6, FN1 raw CPM was higher in four samples and detection was higher in all five; SPARC was higher by both measures in all five samples. These descriptive signals coexisted with zero coherent primary ECM genes and failure of the rounded-DecontX primary-module condition (Figure 4B and Figure S8; Table S6).

### 3.6. The Seven-Gene ECM Programme Localised Predominantly to the Frozen Stromal Compartment

Direct comparison with the independently frozen stromal reference provided a positive localisation control. Stromal cells had higher summed ECM7 raw CPM, higher ECM7 detection, and higher rounded-DecontX ECM7 CPM than the frozen myeloid compartment in all five public PDR samples. The median stromal-minus-myeloid raw ECM7 CPM difference was 14,897.5.

The same direction held for every individual primary ECM gene: COL1A1, COL1A2, COL5A1, LUM, THBS2, FBLN2, and CTGF were each higher in stromal cells by raw CPM, raw detection, and rounded-DecontX CPM in 5/5 samples. Thus, ambient-RNA correction did not transfer the dominant ECM localisation from stromal to myeloid cells.

Synthetic myeloid–stromal mixtures showed the expected high ECM signal and were readily separated from the frozen myeloid states, supporting the ability of the adjudication framework to detect stromal admixture when present (Figure 4C,D; Table S7).

### 3.7. SAM-like Myeloid States in PDR Were Separate from the Direct-ECM Gate

The separately locked SAM-like analysis identified two contextual myeloid states. Cluster 1 met the contextual rule for three of four genes—GPNMB, LIPA, and FABP5—but not SPP1. Cluster 6 met the rule for all four genes. For cluster 6, GPNMB, SPP1, and LIPA were higher by both raw CPM and detection in all five samples, and FABP5 was higher by both measures in four samples. No other cluster met the required three-gene contextual threshold.

These secondary results did not modify the negative direct-ECM gate in GSE165784. They indicate distinct regulatory or scar-associated transcriptional patterns in this PDR dataset only, without establishing equivalent states in either PVR cohort. FN1 and SPARC also remained descriptive secondary ECM markers and did not select or rescue a primary state (Figure 4E; Table S8).

### 3.8. Synthesis of the Two PVR Cohorts and External PDR Context

In the two independent PVR cohorts, the GSE294329 rank-based discovery signal was not supported by total ECM7 absolute burden or detection, and no eligible SCP2582 state met the amended six-gene replication gate. Exact seven-gene replication in SCP2582 remained unevaluable. Separately, the PDR dataset GSE165784 contained no state meeting the complete ECM7 gate and placed the dominant evaluated ECM transcriptional burden in the stromal compartment. Because PDR and PVR differ in disease context, the PDR observation is a localisation reference, not confirmatory replication of PVR biology.

The frozen results do not establish a reproducible ECM7-expressing myeloid transcriptional state under the stated criteria in the sampled PVR membranes. In GSE165784 only, secondary SAM-like PDR states coexist with a negative direct-ECM gate. These findings neither test nor exclude paracrine signalling, transient or spatially restricted expression, protein-level synthesis, programmes outside the panel, or other myeloid roles in fibrosis. Table 2 preserves the distinct cohort roles and decision outcomes.

An exploratory structural-collagen diagnostic reaggregated pseudobulk CPM for COL1A1, COL1A2 and COL5A1 in the same frozen states. Summed raw collagen CPM was higher in GSE294329 cluster 1 in four of five donors, in SCP2582 cluster 0 in all five donors, and in GSE165784 PDR cluster 6 in all five accessions; median within-unit differences were 35.70, 6.40 and 6.47 CPM, respectively. Summed rounded-DecontX collagen CPM was higher in four, two and zero units, respectively. This is a mixed result, not a replication of direct collagen production: raw detection of each structural collagen was not greater in GSE294329 cluster 1 in any donor; in SCP2582 cluster 0 only COL1A1 had both greater raw CPM and detection in four donors; in PDR cluster 6 no collagen gene had both greater raw CPM and detection in four accessions. The archived summaries cannot calculate an any-collagen detection fraction, and PDR accessions are not known independent patients. These descriptive contrasts were not endpoint tests and did not change the frozen ECM7 or amended six-gene decisions (Supplementary Table S10).

In the externally defined 41-gene collagen panel, all 41 features mapped in GSE294329, whereas SCP2582 had 40 assayable features; the one missing SCP2582 gene, COL6A5, had zero raw expression in the GSE294329 comparison. For the frozen GSE294329 candidate cluster 1, raw collagen-panel CPM was higher in two of five donors, any-collagen detection in none, and rounded-DecontX CPM in two. In SCP2582 cluster 1, the corresponding directions were higher in five, four and three of five donors, respectively (median raw CPM difference +4.92; range +1.58 to +18.37). COL8A2 and COL24A1 each had both higher raw CPM and detection in four donors, but COL24A1 was detected in only one to five cluster 1 cells in each of those donors. The complete 44-gene set gave higher raw CPM in four of five donors for the GSE294329 candidate, but higher any-collagen detection in none; SCP2582 cluster 1 had four of five donors higher in raw CPM and three of five in detection. Other frozen SCP2582 clusters showed differing directions (Table S16). This exploratory signal in one SCP2582 state cannot establish concordant direct collagen production across cohorts or amend the failed prespecified six-gene gate.

## 4. Discussion

The two PVR cohorts do not provide concordant support for the prespecified direct matrix-associated myeloid transcriptional programme. The positive GSE294329 rank-score discovery signal remained in the record, but its total ECM7 absolute burden and detection were lower than the comparator in all five donors; no SCP2582 state passed the amended six-gene replication gate. The PDR cohort independently showed stromal localisation of ECM7 transcripts and distinct secondary SAM-like states, but it cannot validate a PVR-specific hypothesis. These observations narrow one transcriptional claim rather than establish the absence of macrophage contributions to vitreoretinal fibrosis.

The discovery discrepancy illustrates a limitation of rank-based scoring. GSE294329 cluster 1 had a median raw UMI ratio of 0.119 relative to other myeloid cells and lost the same-direction UCell pattern after rounding DecontX counts at the locked maxRank. An exploratory cellwise diagnostic reproduced the archived donor effects and quantified matrix-specific rank, detection and rounding patterns (Table S14). Positive cellwise scaling and log normalisation alone cannot reorder genes within a cell, while ambient correction, rounding and differing background expression can do so. Lower complexity remains a plausible contributor, but neither the donor comparisons nor an illustrative simulation proves it caused this cluster’s score. Total ECM7 burden and any-target detection were lower in all five donors despite the locked positive UCell decision. Both findings must remain visible; the post-unblinding adjudication does not erase the discovery record.

SCP2582 is independent at the cohort level but could not provide an exact ECM7 replication because CCN2/CTGF was absent from the deposited processed data. A feature audit confirmed that CCN2/CTGF was already absent from the processed data before our quality-control steps but could not determine the upstream cause (Table S15). The amended six-gene test was negative under its four-part rule. Although SCP2582 cluster 2 showed descriptive FN1 and SPARC elevation, these secondary genes could not rescue the result. Exploratory structural-collagen summaries also show why the composite ECM panel should not be treated as a direct structural-collagen test: some states had higher raw collagen CPM, but they did not meet the original multicomponent gate. These analyses remain exploratory.

The independently curated collagen set adds an important qualification. One SCP2582 state, cluster 1, had positive raw CPM and any-collagen detection directions for the 41-gene exclusion panel across most donors, and two individual collagens showed concordant raw CPM and detection in four donors. The original GSE294329 discovery cluster showed no higher any-collagen detection under that panel, and SCP2582 corrected module CPM was higher in only three donors. The SCP2582 state was highlighted from exploratory comparisons of five eligible clusters; gene detection was sparse for COL24A1, and corresponding biological states across cohorts have not been established. This pattern merits targeted follow-up but does not overturn the frozen negative ECM7/six-gene endpoint or justify a broad claim that alternative collagen-associated myeloid states are absent (Table S16).

In PDR, GSE165784 provided a positive localisation reference: stromal cells had higher summed ECM7 CPM, detection and rounded-DecontX CPM in all five public accessions, and each primary gene showed the same stromal direction. The series description states three patients, whereas five PDR accessions are deposited; their independence as patient-level replicates cannot be established. PDR fibrovascular tissue differs from PVR membrane tissue in origin, cellular composition and disease drivers. The localisation finding is consistent with earlier PDR reanalysis [10,11] and informative about this dataset, but cannot be transported unqualified to PVR.

The secondary SAM-like result further clarifies the meaning of a profibrotic myeloid state. Cluster 6 was coherent for GPNMB, SPP1, LIPA, and FABP5, whereas cluster 1 was coherent for GPNMB, LIPA, and FABP5 but not SPP1. Related scar-associated macrophages have been described in fibrotic liver and lung, where their proximity and signalling to mesenchymal cells can promote collagen deposition [5,6]. Yet the absence of SPP1 coherence in one of the two states argues against treating the panel as a single invariant phenotype, and work in lung injury indicates that GPNMB/SPP1-associated programmes may form part of a broader recruited-macrophage injury response rather than a fibrosis-specific identity [8]. Accordingly, the present analysis does not establish that the GSE165784 states are equivalent to liver or lung scar-associated macrophages, nor does it infer causality from their transcriptional profile. It shows that a prespecified regulatory/scar-associated signal remained detectable when the separately defined direct-ECM phenotype was negative.

Several mechanisms could therefore connect myeloid activation to fibrosis without requiring structural-collagen transcription by the same cells. Macrophages can provide TGF-β- and PDGF-family signals, express SPP1 and other matricellular proteins, remodel matrix through proteases, and regulate fibroblast recruitment, survival, or activation. In infarcted myocardium, lineage-resolved analyses found precisely this combination—a fibrogenic macrophage phenotype with growth-factor and matricellular expression but negligible structural-collagen production [9]. In experimental pulmonary fibrosis, macrophage-derived GPNMB can be retained within fibrotic ECM and activate fibroblasts [21]. These observations provide plausible functional contexts for the GSE165784 SAM-like states, but they are not direct evidence that the same pathways operate in PVR. They reinforce the narrower conceptual point that profibrotic is a functional umbrella, whereas ECM-producing should be reserved for evidence of direct matrix synthesis.

Macrophage-to-myofibroblast transition also remains outside the inferential reach of this study. Co-expression of macrophage and myofibroblast proteins has been reported in proliferative vitreoretinal membranes [2,4], while genetic tracing in an experimental PVR model identified RPE as a major fibroblast source [3]. A single-cell transcriptomic snapshot from surgically excised human tissue cannot establish cellular ancestry, determine whether a dual-positive cell represents a stable transition, or exclude a transient state that occurred earlier in disease. Nor can absence of a coherent ECM7 transcript pattern exclude stored or secreted protein, post-transcriptional regulation, or a rare transition below the detection limits of the sampled material. The present results therefore constrain one transcriptional claim; they do not resolve all possible lineage routes to myofibroblast formation.

The primary PVR comparisons used donors rather than individual cells as the inferential units. The documented analytical sequence supports assessment of the decisions but was not independently verified by a second analyst or externally time-stamped. Because DecontX included outcome genes before clustering, an exploratory fixed-cell sensitivity analysis excluded the protected genes from DecontX in SCP2582 and found substantial reassignment of cells among fixed myeloid states. No alternative donor-eligible state met the necessary raw multigene-coherence rule, although broad lineage assignment, the other cohorts and the corrected-gene criterion were not retested. A separate sensitivity analysis allowing protected genes into myeloid feature selection included only secondary FN1 among its selected features; its alternative states also failed the necessary raw-coherence rule (Table S13). Neither analysis rules out a rare myeloid–stromal transition missed before compartment selection, and the collagen-panel analyses are exploratory.

Each PVR cohort included only five donors, limiting precision and sensitivity to rare, transient or donor-specific states; no formal power calculation was performed. Five PDR accessions were assessed, but their patient independence is uncertain. Cohorts also differed in data processing and platform, and the SCP2582 processed data lacked CCN2/CTGF. The seven-gene panel combines structural collagens with other matrix-associated genes. Protected genes indirectly affected SCP2582 cell-state membership through DecontX in an exploratory fixed-cell sensitivity analysis, and upstream lineage selection and the other cohorts were not retested. Another sensitivity included only secondary FN1 among the alternative integration features; it cannot establish that every possible analytical choice is insensitive to outcome genes. Some exploratory structural-collagen comparisons had positive raw CPM contrasts, so the negative composite gate does not establish absent collagen transcription. Cross-sectional RNA measurements cannot demonstrate cell ancestry, local secretion or collagen protein production, and spatial or protein validation was unavailable. Independent review of analytical and annotation decisions by a second analyst has not been documented.

Larger, prospectively collected PVR donor cohorts with retained raw data could test structural collagen separately from matricellular and regulatory programmes, while keeping the inference unit at the patient level. Spatial RNA and matched protein localisation, nascent-matrix assays and experimental lineage tracing would address mechanisms beyond this cross-sectional analysis. At present, the study supplies no basis for changing retinal surgery, prescribing, or selection of an anti-macrophage treatment; its clinical contribution is to specify the evidence needed before attributing direct matrix synthesis to myeloid cells.

## 5. Conclusions

Across two five-donor PVR cohorts, the prespecified analyses did not show a reproducible ECM7-expressing myeloid state under the defined gates: the positive rank-based discovery result lacked total absolute-expression support, and replication was negative only under an amended six-gene assay. In a separate PDR dataset of five public accessions with uncertain patient independence, ECM7 localised preferentially to stromal cells and secondary SAM-like myeloid states were detected. An exploratory analysis using an external collagen-gene panel found a cluster-specific raw transcriptional signal in SCP2582 without a matching discovery detection pattern. These transcriptional observations do not exclude transient or rare collagen-expressing cells, protein-level matrix synthesis, or paracrine and remodelling roles for macrophages in PVR.

## Figures and Tables

**Table 1:** Dataset roles, biological-replication units, target-programme evaluability, cell retention, and frozen analytical compartments.

Cohort/Role	Biological Donor or Public Accession Unit	Input Representation → Retained	Frozen Analytical Compartment	Primary Target Status
GSE294329/discovery	5 PVR donors	29,813 deposited matrix barcode columns → 23,065 cells	4564 myeloid cells; 8 frozen states, 6 eligible	Exact ECM7 evaluable
SCP2582/independent replication	5 definite epiretinal PVR donors	3738 public-object cells → 3421 cells	1281 myeloid cells; 7 frozen states, 5 eligible	CCN2/CTGF absent; amended six-gene test evaluable
GSE165784/external adjudication	5 public PDR sample accessions	8011 deposited cells → 6817 cells	5105 myeloid and 548 stromal cells; 9 frozen myeloid states, 7 eligible	Exact ECM7 evaluable

Note: Donors were the biological replicates in GSE294329 and SCP2582. Because the GSE165784 series-level patient count conflicts with the five deposited PDR sample records, the public-sample accession was used as the consistency unit. Input representations differ among deposited datasets and are not intended as cross-cohort capture-efficiency comparisons. For GSE294329, 29,813 denotes barcode columns entering this reanalysis and is distinct from the source record’s author-reported 18,213 profiled cells. ECM7 comprised COL1A1, COL1A2, COL5A1, LUM, THBS2, FBLN2, and CCN2/CTGF. The arrow (→) indicates the transition from the input representation to the number of cells retained after quality control and doublet removal.

**Table 2:** Frozen three-cohort evidence synthesis for the prespecified direct-ECM myeloid phenotype.

Cohort	Locked Primary Test	Frozen Result	Interpretation
GSE294329	Exact ECM7 UCell discovery followed by artefact adjudication	Discovery rule met; absolute burden and any-target detection higher in 0/5 donors; rounded-DecontX robustness failed	Rank-based signal retained but not evidence of a coherent direct ECM-producing state
SCP2582	Exact ECM7 not evaluable; amended six-gene A + B + C + D gate evaluable	No eligible frozen state met all four criteria	Independent PVR cohort; amended six-gene gate negative; exact ECM7 replication unevaluable
GSE165784	Exact ECM7 A + B + C + D adjudication	No eligible myeloid state passed; stromal ECM7 higher in 5/5 samples; SAM-like clusters 1 and 6	PDR localisation context: stromal ECM7 predominance and distinct secondary SAM-like states

Note: The positive GSE294329 rank-based discovery record was not overwritten, but post-unblinding absolute-expression and artefact adjudication did not support a direct ECM-producing interpretation. Exact seven-gene replication was not evaluable in SCP2582; the amended six-gene test was evaluable but negative. In GSE165784, the direct-ECM gate was negative in myeloid states, the ECM7 programme localised to the frozen stromal compartment, and secondary SAM-like states were detected. No cross-cohort cell-level inference was performed, and secondary programmes did not rescue a failed primary endpoint.

## Data Availability

The source single-cell datasets are publicly available from the Gene Expression Omnibus (GSE294329 and GSE165784) and Single Cell Portal (SCP2582). Analysis code and available supporting results are deposited in Zenodo record 22072421 (https://zenodo.org/records/22072421; accessed on 30 September 2026). Some large derived analysis objects are absent from this package, so it does not currently enable an exact rerun using the deposited files alone. No new participant data were generated.

## Supplementary Materials

The following supporting information can be downloaded at: https://www.mdpi.com/article/10.3390/bioengineering13101185/s1. The Supplementary Materials preserve target-blind annotation, frozen-state provenance, complete donor/sample-level decision records, stromal localisation, SAM-like results, and the reproducibility and correction history. Figure S1: GSE294329 Target-Blind Broad Annotation; Figure S2: GSE294329 Frozen Myeloid Structure and ECM7 UCell Robustness; Figure S3: GSE294329 Lineage-Identity and Synthetic-Mixture Challenge; Figure S4: SCP2582 Target-Blind Broad Annotation; Figure S5: SCP2582 Frozen Myeloid Provenance; Figure S6: GSE165784 Target-Blind Broad Annotation; Figure S7: GSE165784 Frozen Myeloid Provenance; Figure S8: Detailed GSE165784 Direct-ECM Effects; Figure S9: SCP2582 Frozen-Cluster Technical Scores and Lineage Markers; Table S1: Dataset and Sample Inventory; Table S2: Donor/Sample-Specific QC; Table S3: Frozen Compartments, Eligibility, and Firewall; Table S4: GSE294329 Discovery and Artefact Adjudication; Table S5: SCP2582 Independent Replication; Table S6: GSE165784 Direct-ECM Adjudication; Table S7: GSE165784 Stromal Localisation; Table S8: GSE165784 Secondary SAM-Like Context; Table S9: Reproducibility and Correction History; Table S10: Exploratory Structural Collagen CPM Contrasts; Table S11: Frozen Cluster Inventory; Table S12: Exploratory SCP2582 DecontX Sensitivity and Raw Gate Checks; Table S13: Exploratory Conventional Fixed-Cell Clustering and Raw Gate Checks; Table S14: Exploratory Discovery UCell Rank Diagnostic; Table S15: Original SCP2582 Processed Feature Provenance; Table S16: Exploratory NABA Collagen-Panel Sensitivity.
