# Supplementary material for: Testing a Prespecified Matrix Transcriptional Programme in Myeloid Cells from Proliferative Vitreoretinopathy Membranes: A Donor-Aware Computational Single-Cell Study
Source: Bioengineering (Basel). 2026 Oct 12;13(10):1185. doi: 10.3390/bioengineering13101185 (PMC13702008; doi:10.3390/bioengineering13101185)
Supplement: Supplementary file 1 [file bioengineering-13-01185-s001.zip › PVR_Supplementary_Figures_S1-S9.pdf]

Figure S1. GSE294329 Target-Blind Broad Annotation

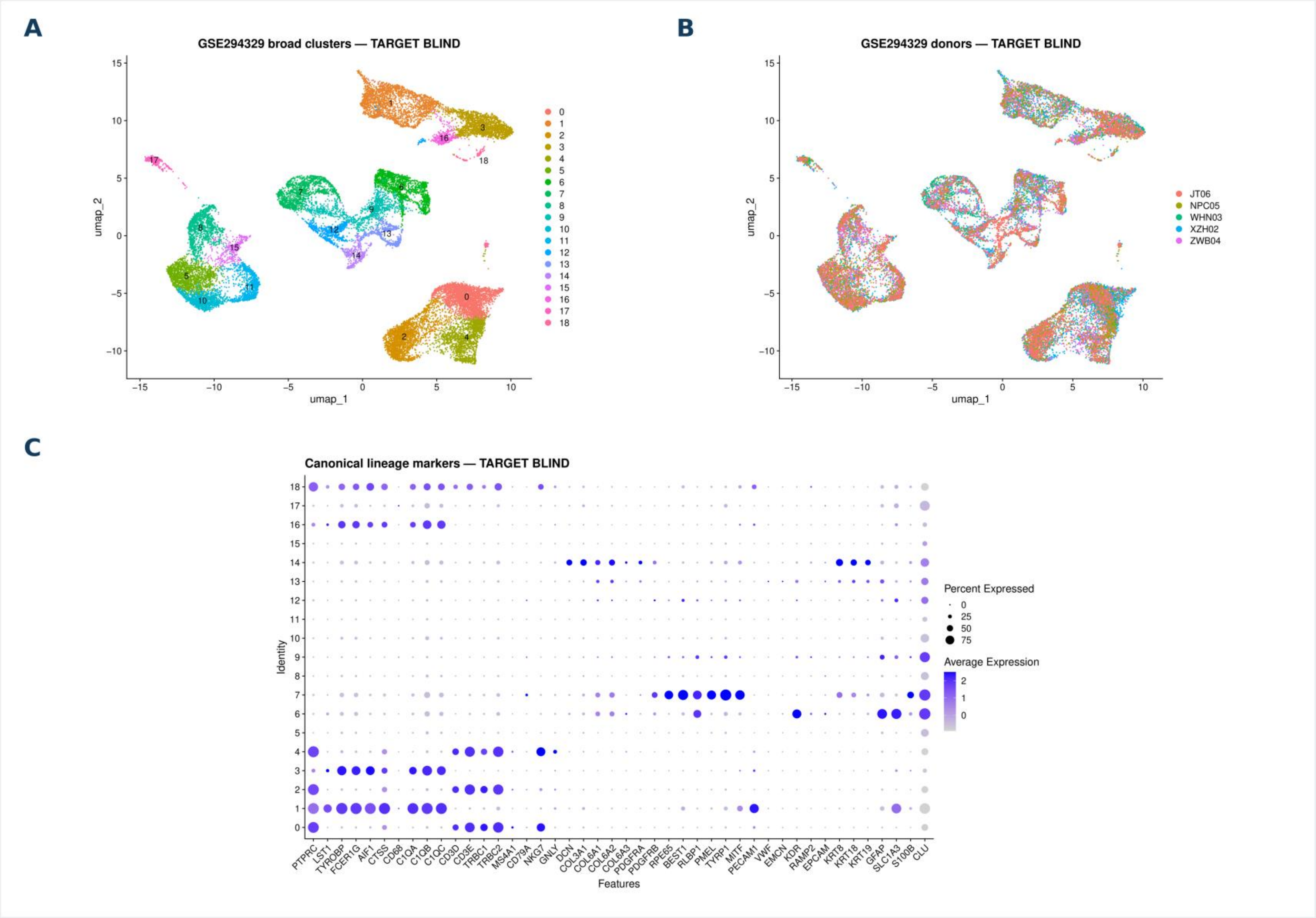

Figure S1. Target-blind broad annotation of GSE294329. (A) UMAP of 23,065 cells retained after donor-specific quality control and scDblFinder doublet removal, coloured by 19 target-blind broad clusters. (B) The same embedding coloured by the five PVR donors. (C) Canonical lineage-marker dot plot used for broad biological annotation. The protected primary ECM genes were excluded from feature selection, dimensionality reduction, clustering, marker assessment and annotation; the displayed structure was frozen before target unblinding.

Figure S2. GSE294329 Frozen Myeloid Structure and ECM7 UCell Robustness

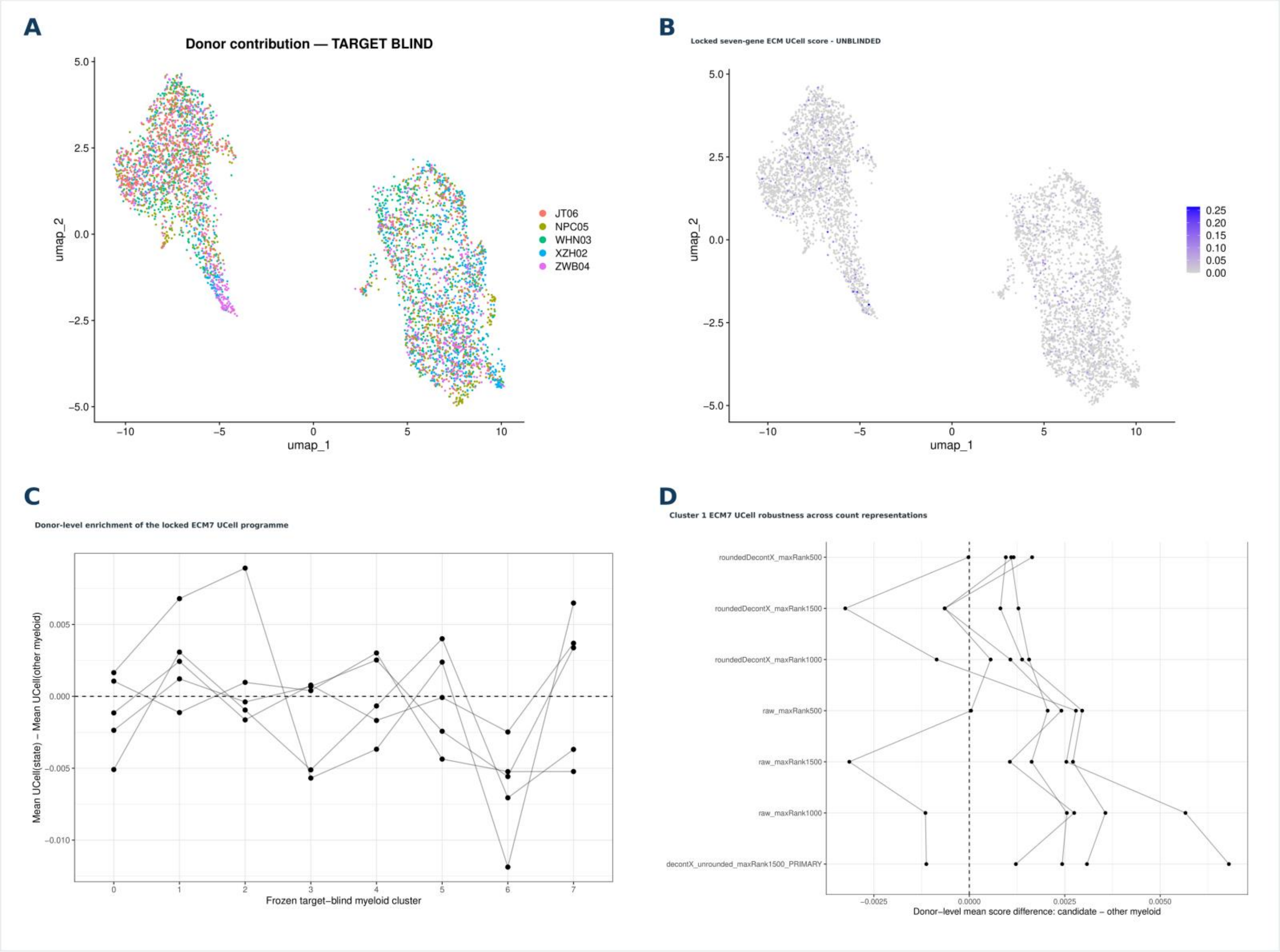

Figure S2. Frozen GSE294329 myeloid structure and ECM7 UCell score robustness. (A) Target-blind UMAP of 4,564 high-confidence myeloid cells coloured by donor. (B) Post-unblinding cell-level overlay of the locked ECM7 UCell score, presented descriptively. (C) Donor-level mean state-minus-other-myeloid ECM7 UCell effects across the frozen clusters; clusters 0-5 were eligible under the locked rule, whereas clusters 6-7 were ineligible. (D) Donor-level cluster 1 effects across raw, unrounded-DecontX and rounded-DecontX representations and maxRank settings. The rounded-DecontX maxRank 1500 setting did not satisfy the same-direction rule. Panels B-D were post-unblinding robustness or adjudication analyses and did not redefine the frozen states or replace candidate cluster 1.

Figure S3. GSE294329 Lineage Identity and Synthetic-Mixture Challenge

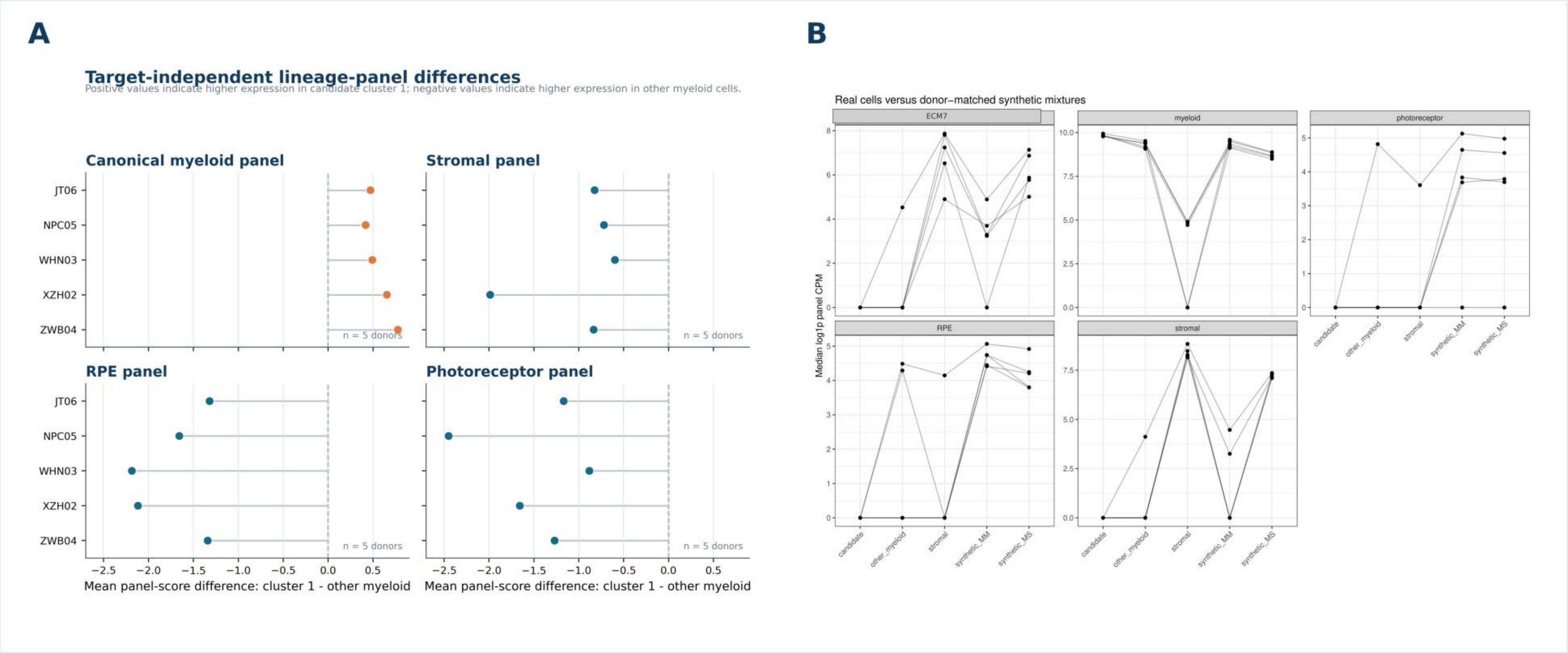

Figure S3. Post-unblinding lineage-identity and synthetic-mixture adjudication in GSE294329. (A) Donor-level candidate-minus-other-myeloid differences in the mean scores of fixed target-independent canonical myeloid, stromal, retinal pigment epithelium (RPE) and photoreceptor panels. Candidate cluster 1 had a higher canonical myeloid-panel score and lower scores for each non-myeloid panel in all five donors. (B) Median log1p panel CPM in real candidate cluster 1, other myeloid cells, stromal cells and donor-matched synthetic myeloid-myeloid (MM) and myeloid-stromal (MS) mixtures. Cluster 1 retained canonical myeloid identity and did not phenocopy the stromal-admixture controls. These analyses were used for artifact adjudication only and did not modify the frozen candidate state.

Figure S4. SCP2582 Target-Blind Broad Annotation

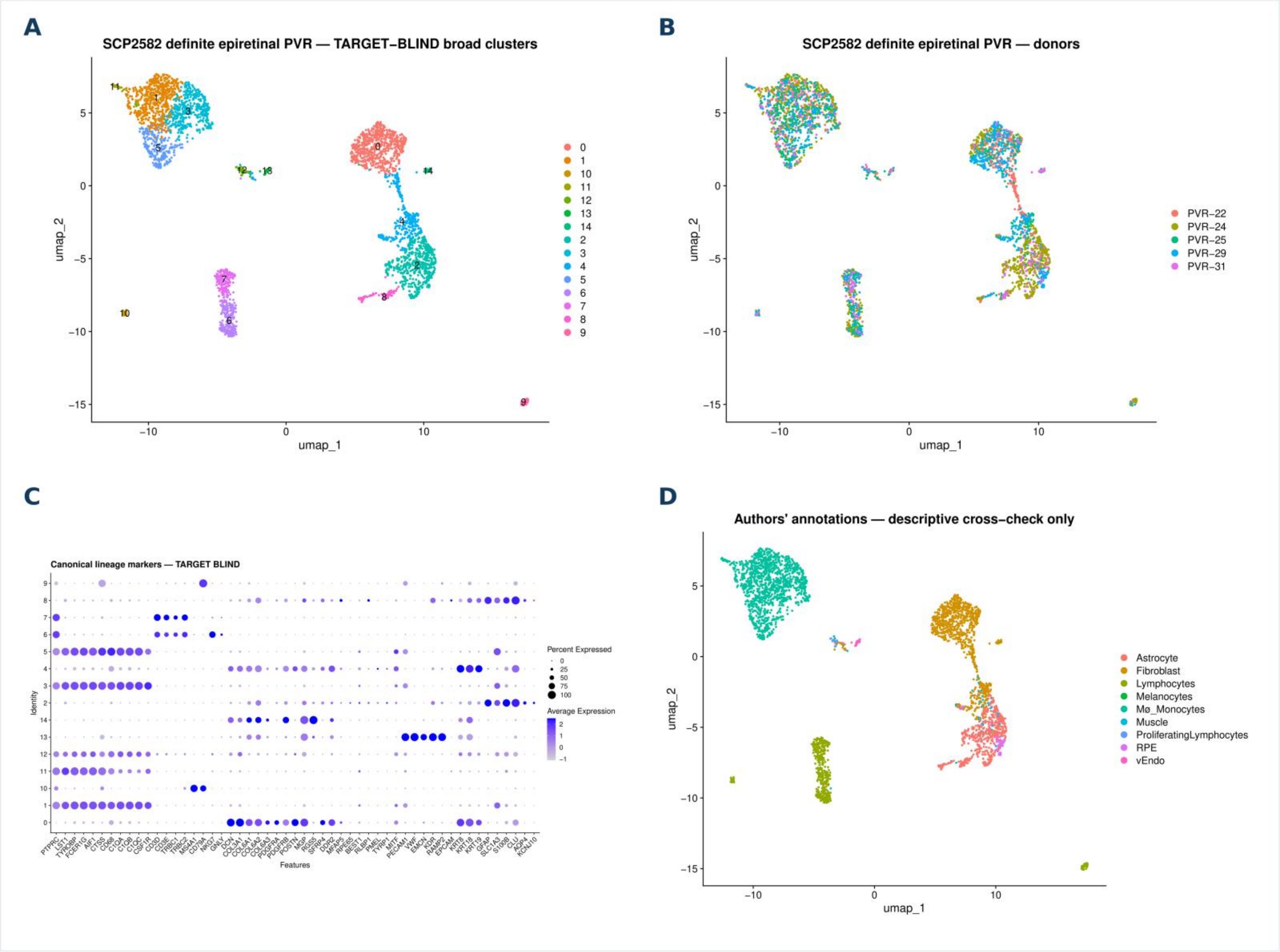

Figure S4. Target-blind broad annotation of SCP2582. (A) UMAP of 3,421 post-QC singlets from five definite epiretinal PVR donors, coloured by 15 target-blind broad clusters. (B) The same embedding coloured by donor. (C) Canonical lineage-marker dot plot used for target-blind annotation. (D) Deposited author annotations projected onto the same cells as a descriptive cross-check only; they were not used to select the frozen myeloid compartment. Protected target genes were excluded from target-blind structure and annotation.

Figure S5. SCP2582 Frozen Myeloid Provenance

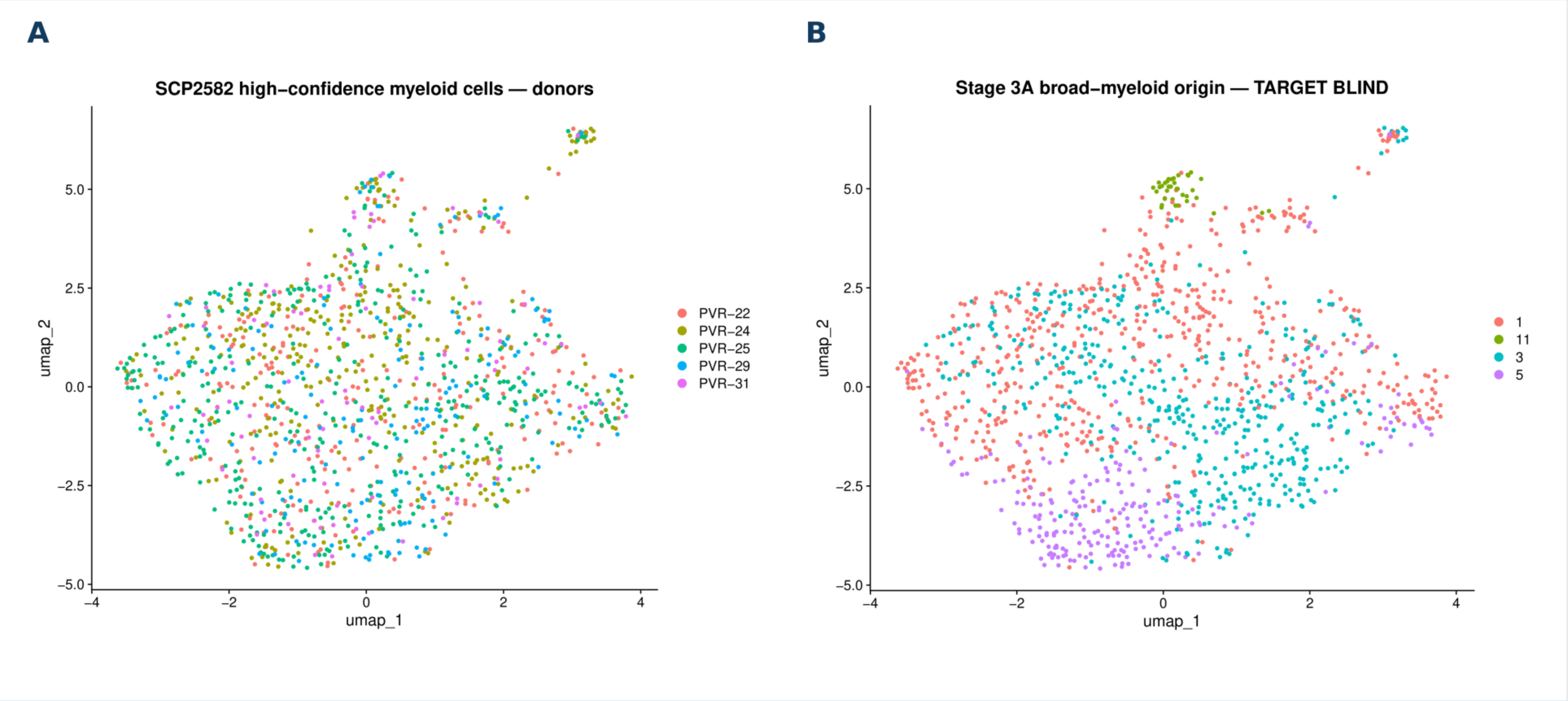

Figure S5. Target-blind provenance of the frozen SCP2582 myeloid compartment. (A) UMAP of 1,281 high-confidence myeloid cells coloured by the five definite epiretinal PVR donors. (B) The same embedding coloured by the Stage 3A target-blind broad-cluster origin. High-confidence myeloid cells were derived from broad clusters 1, 3, 5 and 11. The compartment and its subclusters were frozen before target unblinding.

Figure S6. GSE165784 Target-Blind Broad Annotation

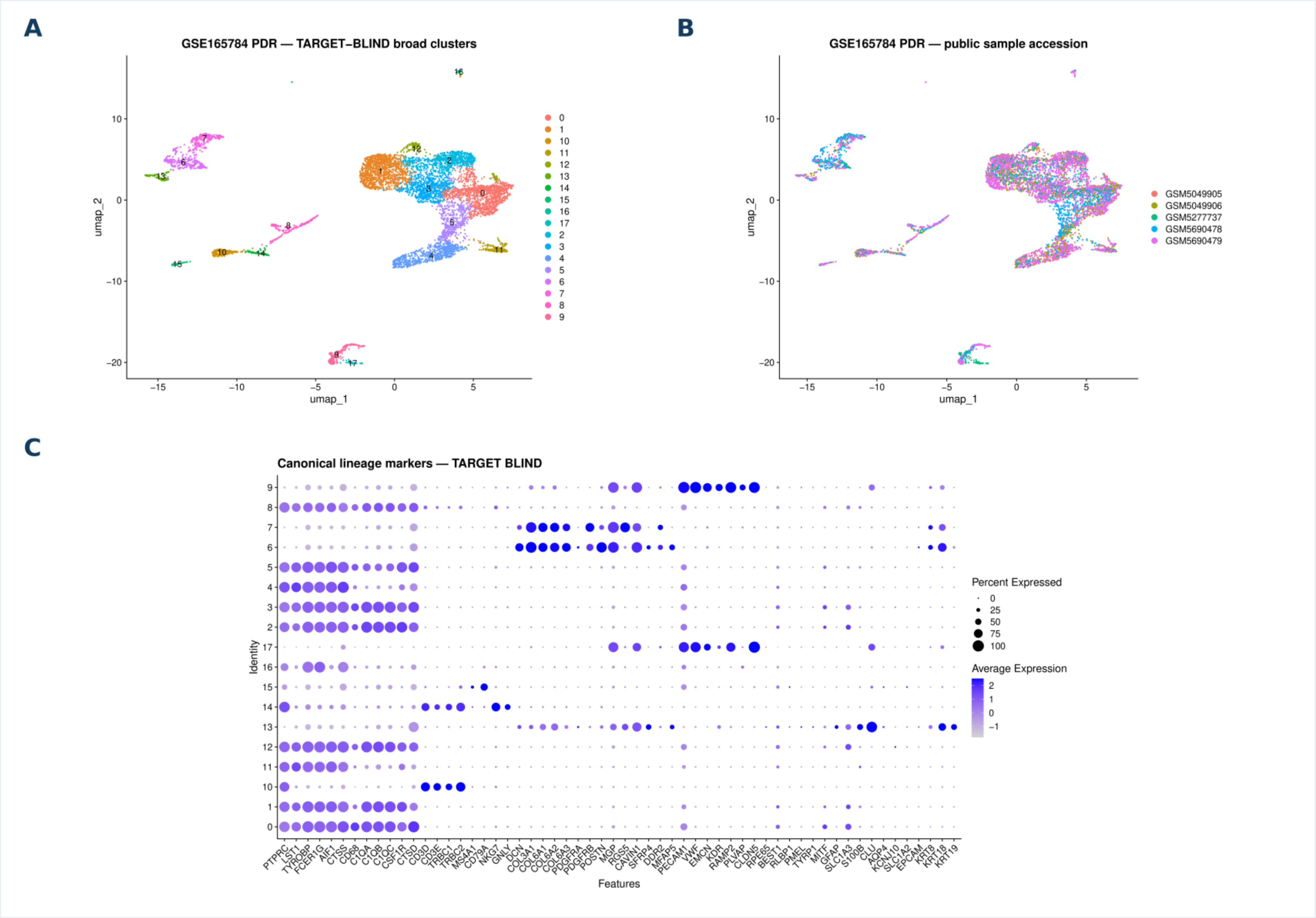

Figure S6. Target-blind broad annotation of GSE165784. (A) UMAP of 6,817 cells retained after sample-specific quality control and scDblFinder doublet removal, coloured by 18 target-blind broad clusters. (B) The same embedding coloured by five public PDR sample accessions. (C) Canonical lineage-marker dot plot used for broad biological annotation. Protected primary ECM genes and contextual SAM-like targets were excluded from target-blind structure and annotation. Results are described across public samples without assuming that the five accessions represent five independent donors.

Figure S7. GSE165784 Frozen Myeloid Provenance

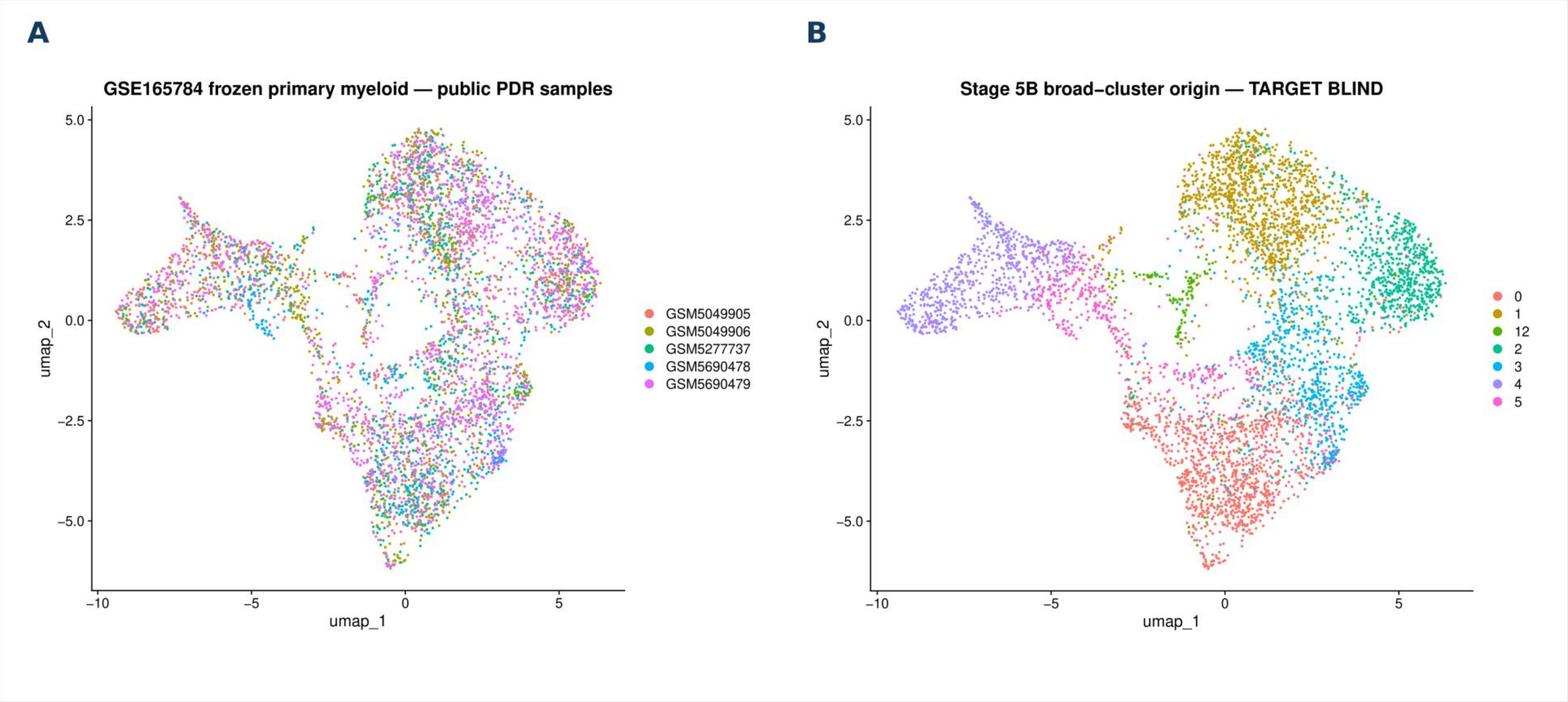

Figure S7. Target-blind provenance of the frozen GSE165784 primary macrophage/monocyte compartment. (A) UMAP of 5,105 frozen primary myeloid cells coloured by five public PDR sample accessions. (B) The same embedding coloured by the Stage 5B target-blind broad-cluster origin. The compartment and its subclusters were frozen before target unblinding, and the target-blind audit identified no protected-feature hits. Results are framed across public samples rather than independent donors.

Figure S8. Detailed GSE165784 Direct-ECM Effects

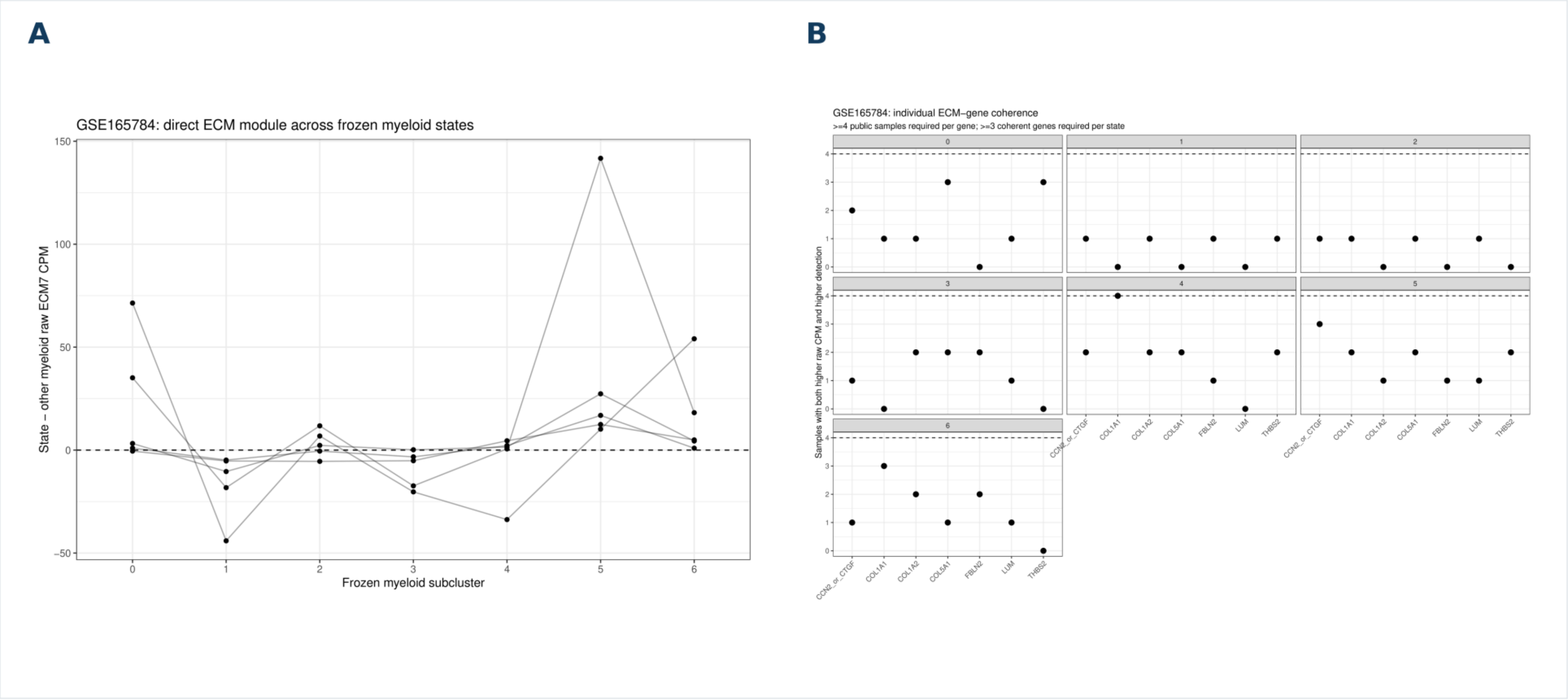

Figure S8. Detailed direct-ECM effects in GSE165784. (A) Sample-level state-minus-other-myeloid differences in raw summed ECM7 CPM for eligible frozen myeloid subclusters 0-6; each connected trajectory represents one public PDR sample. (B) Number of samples in which both raw CPM and detection were higher in the state for each primary ECM gene and subcluster. The dashed line marks the locked per-gene coherence threshold of four samples. Only COL1A1 in cluster 4 met that per-gene threshold, and no state reached the criterion of at least three coherent primary ECM genes. The four-sample line in panel B is the gene-level threshold; the direct-ECM gate's criterion C requires at least three coherent genes per state.

Figure S9. SCP2582 Frozen-Cluster Technical Scores and Lineage Markers

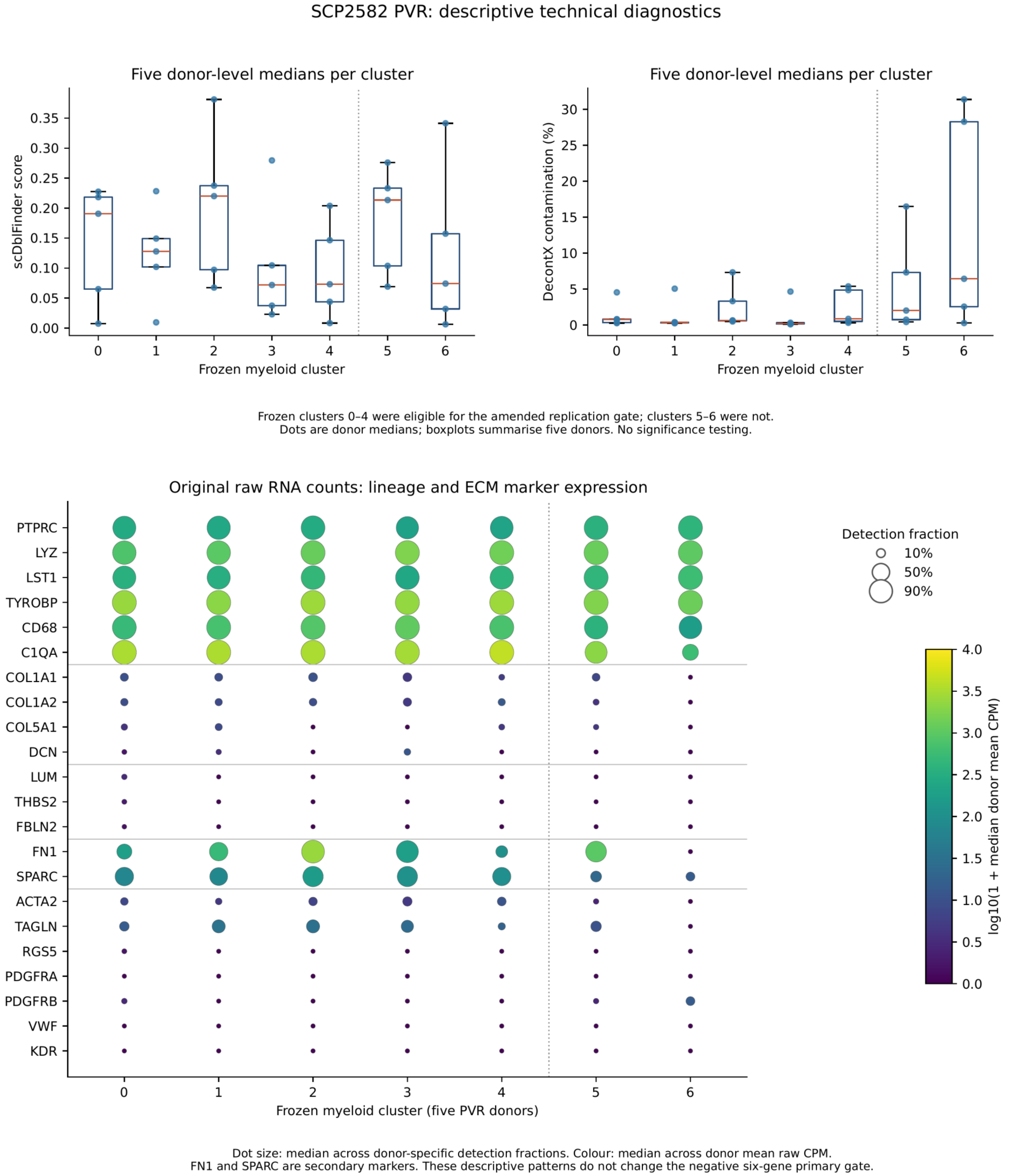

Figure S9. Descriptive checks in frozen SCP2582 myeloid states from five PVR donors. Upper panels: scDblFinder doublet scores and DecontX contamination estimates by cluster; dots are donor medians and boxplots summarise donor medians. Clusters 0–4 met the frozen replication-presence rule; clusters 5 and 6 did not. Lower panel: expression of myeloid, stromal and extracellular-matrix markers from raw RNA counts. Dot area denotes median donor-level detection; colour denotes log10(1 + median donor mean CPM). FN1 and SPARC were secondary markers and cannot rescue the negative amended six-gene gate. These descriptive distributions cannot exclude rare doublets, ambient transcripts or transient myeloid–stromal states.
