# Supplementary material for: Testing a Prespecified Matrix Transcriptional Programme in Myeloid Cells from Proliferative Vitreoretinopathy Membranes: A Donor-Aware Computational Single-Cell Study
Source: Bioengineering (Basel). 2026 Oct 12;13(10):1185. doi: 10.3390/bioengineering13101185 (PMC13702008; doi:10.3390/bioengineering13101185)
Supplement: Supplementary file 1 [file bioengineering-13-01185-s001.zip › PVR_Supplementary_Tables_S1-S16.pdf]

SUPPLEMENTARY MATERIALS

Supplementary Tables S1–S16

Testing a Prespecified Matrix Transcriptional Programme in Myeloid Cells from Proliferative Vitreoretinopathy Membranes: A Donor-Aware Computational Single-Cell Study

Georgios D. Panos

Table S1. Dataset and Sample Inventory

TARGET-BLIND COHORT DEFINITION

Accession identifiers, deposited anatomy, primary versus sensitivity inclusion, and cohort role. GSE165784 sample accessions are not asserted to be independent donors.

GSE294329 discovery cohort

GSM	Donor	Biosample	SRX	Sex	PVR Stage
GSM8902421	XZH02	SAMN47878066	SRX28362304	male	C
GSM8902422	WHN03	SAMN47878065	SRX28362303	male	C
GSM8902423	ZWB04	SAMN47878064	SRX28362302	male	C
GSM8902424	NPC05	SAMN47878063	SRX28362301	male	C
GSM8902425	JT06	SAMN47878062	SRX28362300	male	C

SCP2582 frozen anatomical roles

Donor	Orig. Ident.	Tissue	n_cells	Author Annotated Myeloid Cells	Replication Role	Informative Myeloid Primary
PVR-03232022	PVR0323	new	1606	477	SENSITIVITY_UNRESOLVED_NEW	TRUE
PVR-22	Retina1	epiretinal	684	279	PRIMARY_EPIRETINAL	TRUE
PVR-24	20190503	epiretinal	1247	374	PRIMARY_EPIRETINAL	TRUE
PVR-25	20190614	epiretinal	820	434	PRIMARY_EPIRETINAL	TRUE
PVR-26	20190722	subretinal	372	110	ANATOMICAL_SENSITIVITY_SUBRETINAL	TRUE
PVR-29	20190906	epiretinal	692	202	PRIMARY_EPIRETINAL	TRUE
PVR-30	ciliary	CB	656	399	ANATOMICAL_SENSITIVITY_CILIARY_BODY	TRUE

Donor	Orig. Ident.	Tissue	n_cells	Author Annotated Myeloid Cells	Replication Role	Informative Myeloid Primary
PVR-31	epi	epiretinal	295	102	PRIMARY_EPIRETINAL	TRUE

GSE165784 repository inventory and independence caveat

GSM	Title	Disease	Age	Role	Donor Independence Status
GSM5049904	PVR-FM	PVR	59	DESCRIPTIVE_PVR_SINGLE_SAMPLE	ONE_PVR_SAMPLE; no donor-level replication possible
GSM5049905	PDR-FM-1	PDR	51	PRIMARY_PDR_ADJUDICATION_SAMPLE	UNRESOLVED: five PDR GSM accessions but GEO series overall-design text states three PDR patients
GSM5049906	PDR-FM-2	PDR	51	PRIMARY_PDR_ADJUDICATION_SAMPLE	UNRESOLVED: five PDR GSM accessions but GEO series overall-design text states three PDR patients
GSM5277737	PDR-FM-3	PDR	47	PRIMARY_PDR_ADJUDICATION_SAMPLE	UNRESOLVED: five PDR GSM accessions but GEO series overall-design text states three PDR patients
GSM5690478	PDR-FM-4	PDR	52	PRIMARY_PDR_ADJUDICATION_SAMPLE	UNRESOLVED: five PDR GSM accessions but GEO series overall-design text states three PDR patients
GSM5690479	PDR-FM-5	PDR	53	PRIMARY_PDR_ADJUDICATION_SAMPLE	UNRESOLVED: five PDR GSM accessions but GEO series overall-design text states three PDR patients

Table S2. Donor/Sample-Specific QC

TARGET-BLIND PREPROCESSING

QC used log10-transformed detected-feature and UMI bounds defined per donor/sample as median - 3 robust MAD to median + 4 robust MAD, with fixed minima of 300 features and 500 UMIs and mitochondrial fraction <=20%.

Reconstructed frozen QC bounds and archived retention

Part 1 of 2 (source columns 1, 2 and 3–10; identifier columns repeated)

Cohort	Donor or public unit	Input Cells	log10_nFeature_lower	log10_nFeature_upper	log10_nCount_lower	log10_nCount_upper	fixed_min_nFeature	fixed_min_nCount	max_percent_mt
GSE294329	XZH02	7051	2.47856649559	4.49999524871	2.69983772587	5.24872614339	300	500	20
GSE294329	WHN03	5626	2.47856649559	4.86665782145	2.69983772587	5.82637314743	300	500	20
GSE294329	ZWB04	5354	2.47856649559	4.71705204389	2.69983772587	5.47586299891	300	500	20
GSE294329	NPC05	4767	2.47856649559	4.49079182816	2.69983772587	5.22039023486	300	500	20
GSE294329	JT06	7015	2.47856649559	4.77537275188	2.69983772587	5.51593451064	300	500	20
SCP2582	PVR-22	684	3.0005161801	4.43000507937	3.42708588781	5.62806977988	300	500	20
SCP2582	PVR-24	1247	2.61410637742	4.4117675996	3.04218943096	5.21825260056	300	500	20
SCP2582	PVR-25	820	2.92544545054	4.26486134121	3.21362680251	5.43942116957	300	500	20
SCP2582	PVR-29	692	2.91751479673	4.54817756534	2.98805749532	6.1899094099	300	500	20
SCP2582	PVR-31	295	2.63691899006	4.44219461258	2.69983772587	5.86747564943	300	500	20
GSE165784	GSM5049905	1765	2.58555122975	4.00322242316	2.84281677973	4.70124782082	300	500	20
GSE165784	GSM5049906	1413	2.76870958421	3.80845298171	3.01864801667	4.618754475	300	500	20
GSE165784	GSM5277737	1058	2.51101526287	3.86010917205	2.69983772587	4.54654678747	300	500	20
GSE165784	GSM5690478	1255	2.86334831468	4.05104127298	3.13638925623	4.95862617949	300	500	20
GSE165784	GSM5690479	2520	2.81389929143	3.9495237405	3.04072566248	4.86592096551	300	500	20

Part 2 of 2 (source columns 1, 2 and 11–16; identifier columns repeated)

Cohort	donor_or_public_unit	reconstructed_post_basic_QC	archived_post_basic_QC	reconstruction_matches_archive	scDblFinder_doublets	post_doublet	median_DecontX_contamination
GSE294329	XZH02	5864	5864	TRUE	329	5535	0.040994
GSE294329	WHN03	4182	4182	TRUE	240	3942	0.035548
GSE294329	ZWB04	4618	4618	TRUE	273	4345	0.034787
GSE294329	NPC05	4305	4305	TRUE	245	4060	0.034907
GSE294329	JT06	5612	5612	TRUE	429	5183	0.046402
SCP2582	PVR-22	684	684	TRUE	26	658	0.041762

Cohort	donor_or_public_ unit	reconstructed_post_ basic_QC	archived_post_ basic_QC	reconstruction_ matches_archive	scDblFinder_ doublets	post_doublet	median_DecontX_ contamination
SCP2582	PVR-24	1181	1181	TRUE	45	1136	0.002703
SCP2582	PVR-25	774	774	TRUE	41	733	0.003889
SCP2582	PVR-29	649	649	TRUE	22	627	0.007217
SCP2582	PVR-31	287	287	TRUE	20	267	0.006490
GSE165784	GSM5049905	1230	1230	TRUE	54	1176	0.006328
GSE165784	GSM5049906	1316	1316	TRUE	55	1261	0.002482
GSE165784	GSM5277737	971	971	TRUE	36	935	0.005312
GSE165784	GSM5690478	1176	1176	TRUE	46	1130	0.022375
GSE165784	GSM5690479	2447	2447	TRUE	132	2315	0.030038

Table S3. Frozen Compartments, Eligibility, and Firewall

TARGET-BLIND FREEZE

Cell compartments, state presence and eligibility, and protected-feature audits recorded before target unblinding.

Frozen compartment summary

Cohort	Primary Units	post-QC singlets	Frozen Myeloid Cells	Frozen Stromal Cells	Frozen Myeloid States	Eligible States	Primary Resolution
GSE294329	5 donors	23065	4564		8	0-5 (6 states)	0.6
SCP2582	5 definite epiretinal donors	3421	1281		7	0-4 (5 states)	0.6
GSE165784	5 public PDR samples	6817	5105	548	9	0-6 (7 states)	0.6

GSE294329 state eligibility

Cluster	n_donors_present	eligible_locked
0	5	TRUE
1	5	TRUE
2	5	TRUE
3	5	TRUE
4	5	TRUE
5	5	TRUE
6	3	FALSE
7	2	FALSE

GSE294329 donor-state presence

Cluster	Donor	n_state	n_donor	Proportion	present_locked
0	JT06	109	818	0.133251833741	TRUE
0	NPC05	213	772	0.275906735751	TRUE
0	WHN03	195	1058	0.184310018904	TRUE
0	XZH02	302	1001	0.301698301698	TRUE
0	ZWB04	220	915	0.24043715847	TRUE
1	JT06	262	818	0.320293398533	TRUE
1	NPC05	147	772	0.190414507772	TRUE
1	WHN03	251	1058	0.237240075614	TRUE

Cluster	Donor	n_state	n_donor	Proportion	present_locked
1	XZH02	77	1001	0.0769230769231	TRUE
1	ZWB04	155	915	0.169398907104	TRUE
2	JT06	108	818	0.132029339853	TRUE
2	NPC05	91	772	0.117875647668	TRUE
2	WHN03	147	1058	0.138941398866	TRUE
2	XZH02	127	1001	0.126873126873	TRUE
2	ZWB04	206	915	0.225136612022	TRUE
3	JT06	74	818	0.0904645476773	TRUE
3	NPC05	107	772	0.138601036269	TRUE
3	WHN03	114	1058	0.10775047259	TRUE
3	XZH02	222	1001	0.221778221778	TRUE
3	ZWB04	113	915	0.12349726776	TRUE
4	JT06	69	818	0.0843520782396	TRUE
4	NPC05	82	772	0.10621761658	TRUE
4	WHN03	208	1058	0.196597353497	TRUE
4	XZH02	132	1001	0.131868131868	TRUE
4	ZWB04	99	915	0.108196721311	TRUE
5	JT06	168	818	0.205378973105	TRUE
5	NPC05	87	772	0.112694300518	TRUE
5	WHN03	121	1058	0.114366729679	TRUE
5	XZH02	83	1001	0.0829170829171	TRUE
5	ZWB04	105	915	0.114754098361	TRUE
6	JT06	8	818	0.00977995110024	FALSE
6	NPC05	14	772	0.0181347150259	TRUE
6	WHN03	13	1058	0.0122873345936	TRUE
6	XZH02	55	1001	0.0549450549451	TRUE
6	ZWB04	9	915	0.00983606557377	FALSE
7	JT06	20	818	0.0244498777506	TRUE
7	NPC05	31	772	0.0401554404145	TRUE
7	WHN03	9	1058	0.00850661625709	FALSE
7	XZH02	3	1001	0.002997002997	FALSE

Cluster	Donor	n_state	n_donor	Proportion	present_locked
7	ZWB04	8	915	0.00874316939891	FALSE

SCP2582 state eligibility

Cluster	n_donors_present	eligible_for_stage3C
0	5	TRUE
1	5	TRUE
2	5	TRUE
3	4	TRUE
4	4	TRUE
5	3	FALSE
6	0	FALSE

SCP2582 donor-state presence

Cluster	Donor	n_state	n_donor	Proportion	present_locked
0	PVR-22	130	271	0.479704797048	TRUE
0	PVR-24	188	349	0.538681948424	TRUE
0	PVR-25	91	395	0.230379746835	TRUE
0	PVR-29	47	178	0.26404494382	TRUE
0	PVR-31	40	88	0.454545454545	TRUE
1	PVR-22	53	271	0.19557195572	TRUE
1	PVR-24	50	349	0.143266475645	TRUE
1	PVR-25	65	395	0.164556962025	TRUE
1	PVR-29	47	178	0.26404494382	TRUE
1	PVR-31	19	88	0.215909090909	TRUE
2	PVR-22	44	271	0.162361623616	TRUE
2	PVR-24	20	349	0.0573065902579	TRUE
2	PVR-25	80	395	0.20253164557	TRUE
2	PVR-29	25	178	0.140449438202	TRUE
2	PVR-31	10	88	0.113636363636	TRUE
3	PVR-22	14	271	0.0516605166052	TRUE

Cluster	Donor	n_state	n_donor	Proportion	present_locked
3	PVR-24	21	349	0.0601719197708	TRUE
3	PVR-25	63	395	0.159493670886	TRUE
3	PVR-29	27	178	0.151685393258	TRUE
3	PVR-31	6	88	0.0681818181818	FALSE
4	PVR-22	14	271	0.0516605166052	TRUE
4	PVR-24	32	349	0.0916905444126	TRUE
4	PVR-25	37	395	0.0936708860759	TRUE
4	PVR-29	16	178	0.0898876404494	TRUE
4	PVR-31	6	88	0.0681818181818	FALSE
5	PVR-22	11	271	0.0405904059041	TRUE
5	PVR-24	29	349	0.0830945558739	TRUE
5	PVR-25	50	395	0.126582278481	TRUE
5	PVR-29	9	178	0.0505617977528	FALSE
5	PVR-31	6	88	0.0681818181818	FALSE
6	PVR-22	5	271	0.0184501845018	FALSE
6	PVR-24	9	349	0.025787965616	FALSE
6	PVR-25	9	395	0.0227848101266	FALSE
6	PVR-29	7	178	0.0393258426966	FALSE
6	PVR-31	1	88	0.0113636363636	FALSE

GSE165784 state eligibility

Cluster	n_samples_present	eligible_for_stage5D
0	5	TRUE
1	5	TRUE
2	5	TRUE
3	5	TRUE
4	5	TRUE
5	5	TRUE
6	5	TRUE
7	3	FALSE
8	2	FALSE

GSE165784 sample-state presence

Cluster	public_unit	n_state	n_sample_myeloid	Proportion	present_locked
0	GSM5049905	139	911	0.152579582876	TRUE
0	GSM5049906	266	1063	0.250235183443	TRUE
0	GSM5277737	171	616	0.277597402597	TRUE
0	GSM5690478	66	687	0.0960698689956	TRUE
0	GSM5690479	406	1828	0.222100656455	TRUE
1	GSM5049905	176	911	0.193194291987	TRUE
1	GSM5049906	174	1063	0.163687676388	TRUE
1	GSM5277737	147	616	0.238636363636	TRUE
1	GSM5690478	161	687	0.234352256186	TRUE
1	GSM5690479	306	1828	0.167396061269	TRUE
2	GSM5049905	128	911	0.140504939627	TRUE
2	GSM5049906	118	1063	0.111006585136	TRUE
2	GSM5277737	71	616	0.11525974026	TRUE
2	GSM5690478	60	687	0.0873362445415	TRUE
2	GSM5690479	304	1828	0.166301969365	TRUE
3	GSM5049905	131	911	0.143798024149	TRUE
3	GSM5049906	179	1063	0.168391345249	TRUE
3	GSM5277737	24	616	0.038961038961	TRUE
3	GSM5690478	203	687	0.295487627365	TRUE
3	GSM5690479	136	1828	0.074398249453	TRUE
4	GSM5049905	92	911	0.100987925357	TRUE
4	GSM5049906	79	1063	0.0743179680151	TRUE
4	GSM5277737	106	616	0.172077922078	TRUE
4	GSM5690478	101	687	0.147016011645	TRUE
4	GSM5690479	187	1828	0.102297592998	TRUE
5	GSM5049905	174	911	0.190998902305	TRUE
5	GSM5049906	108	1063	0.101599247413	TRUE
5	GSM5277737	50	616	0.0811688311688	TRUE
5	GSM5690478	25	687	0.0363901018923	TRUE

Cluster	public_unit	n_state	n_sample_myeloid	Proportion	present_locked
5	GSM5690479	181	1828	0.0990153172867	TRUE
6	GSM5049905	62	911	0.0680570801317	TRUE
6	GSM5049906	65	1063	0.0611476952023	TRUE
6	GSM5277737	38	616	0.0616883116883	TRUE
6	GSM5690478	60	687	0.0873362445415	TRUE
6	GSM5690479	207	1828	0.113238512035	TRUE
7	GSM5049905	6	911	0.00658616904501	FALSE
7	GSM5049906	38	1063	0.035747883349	TRUE
7	GSM5277737	7	616	0.0113636363636	FALSE
7	GSM5690478	11	687	0.0160116448326	TRUE
7	GSM5690479	48	1828	0.0262582056893	TRUE
8	GSM5049905	3	911	0.0032930845225	FALSE
8	GSM5049906	36	1063	0.0338664158043	TRUE
8	GSM5277737	2	616	0.00324675324675	FALSE
8	GSM5690478	0	687	0	FALSE
8	GSM5690479	53	1828	0.0289934354486	TRUE

SCP2582 broad firewall

Component	n_rows_or_features	protected_hits
integration_features	3000	0
marker_output	34899	0

SCP2582 myeloid firewall

Component	n_rows_or_features	protected_hits
integration_features	2000	0
marker_output	5971	0
frozen_centroid_features	2000	0

GSE165784 broad firewall

Component	n_rows_or_features	protected_hits
integration_features	3000	0
marker_output	28262	0

GSE165784 myeloid firewall

Component	n_rows_or_features	protected_hits
integration_features	2500	0
marker_output	7586	0
frozen_centroid_features	2500	0

Table S4. GSE294329 Discovery and Artefact Adjudication

PRESPECIFIED DISCOVERY + POST-UNBLINDING ADJUDICATION

The Stage 2A candidate remains frozen as cluster 1. Stage 2B analyses are sensitivity/adjudication only. CPM-first correction outputs supersede the legacy pseudobulk implementation.

Locked cluster eligibility

Cluster	n_donors_present	eligible_locked
0	5	TRUE
1	5	TRUE
2	5	TRUE
3	5	TRUE
4	5	TRUE
5	5	TRUE
6	3	FALSE
7	2	FALSE

Locked discovery summary

Cluster	n_informative_ donors	n_donors_pre sent	eligible_locked	median_delta _mean_ UCell	min_delta_m ean_ UCell	max_delta_ mean_ UCell	donors_positive _ mean	median_delta _ median_UCell	donors_positive _ median	is_selected_ candidate	discovery_supp ort_ locked
0	5	5	TRUE	-0.0011	-0.0051	0.0016	2	0	0	FALSE	FALSE
1	5	5	TRUE	0.0024	-0.0011	0.0068	4	0	0	TRUE	TRUE
2	5	5	TRUE	-0.0004	-0.0016	0.0089	2	0	0	FALSE	FALSE
3	5	5	TRUE	0.0004	-0.0057	0.0008	3	0	0	FALSE	FALSE
4	5	5	TRUE	-0.0007	-0.0037	0.0030	2	0	0	FALSE	FALSE
5	5	5	TRUE	-0.0001	-0.0044	0.0040	2	0	0	FALSE	FALSE
6	5	3	FALSE	-0.0056	-0.0119	-0.0025	0	0	0	FALSE	FALSE
7	5	2	FALSE	0.0034	-0.0052	0.0065	3	0	0	FALSE	FALSE

Candidate donor effects

Cluster	Donor	n_state	n_other	mean_state	mean_other	delta_mean_UCell	median_state	median_other	delta_median_UCel l
1	JT06	262	556	0.0091	0.0060	0.0031	0	0	0
1	NPC05	147	625	0.0044	0.0020	0.0024	0	0	0

Cluster	Donor	n_state	n_other	mean_state	mean_other	delta_mean_UCell	median_state	median_other	delta_median_UCell
1	WHN03	251	807	0.0064	0.0052	0.0012	0	0	0
1	XZH02	77	924	0.0175	0.0107	0.0068	0	0	0
1	ZWB04	155	760	0.0042	0.0054	-0.0011	0	0	0

Corrected seven-gene burden and detection

Part 1 of 4 (source columns 1 and 2–10; identifier columns repeated)

Donor	group_candidate	n_cells_candidate	mean_raw_7gene_counts_candidate	median_raw_7gene_counts_candidate	mean_raw_7gene_log1pCPM_candidate	median_raw_7gene_log1pCPM_candidate	pct_any_raw_target_candidate	mean_roundedDecontX_7gene_counts_candidate	median_roundedDecontX_7gene_counts_candidate
JT06	candidate	262	0.1107	0	0.6478	0	0.0916030534351	0.0382	0
NPC05	candidate	147	0.0544	0	0.2413	0	0.0340136054422	0.0408	0
WHN03	candidate	251	0.0916	0	0.4188	0	0.0597609561753	0.0598	0
XZH02	candidate	77	0.2727	0	1.1478	0	0.168831168831	0.0519	0
ZWB04	candidate	155	0.0516	0	0.3042	0	0.0451612903226	0.0387	0

Part 2 of 4 (source columns 1 and 11–19; identifier columns repeated)

Donor	mean_roundedDecontX_7gene_log1pCPM_candidate	median_roundedDecontX_7gene_log1pCPM_candidate	pct_any_roundedDecontX_target_candidate	group_other	n_cells_other	mean_raw_7gene_counts_other	median_raw_7gene_counts_other	mean_raw_7gene_log1pCPM_other	median_raw_7gene_log1pCPM_other
JT06	0.2595	0	0.0381679389313	other_myeloid	556	0.5863	0	1.5605	0
NPC05	0.1858	0	0.0272108843537	other_myeloid	625	0.4768	0	1.3704	0
WHN03	0.2820	0	0.0398406374502	other_myeloid	807	0.4845	0	1.5099	0
XZH02	0.2648	0	0.038961038961	other_myeloid	924	1.7316	1	3.3531	4.5288
ZWB04	0.2293	0	0.0322580645161	other_myeloid	760	0.5092	0	1.4191	0

Part 3 of 4 (source columns 1 and 20–28; identifier columns repeated)

Donor	pct_any_raw_target_other	mean_roundedDecontX_7gene_counts_other	median_roundedDecontX_7gene_counts_other	mean_roundedDecontX_7gene_log1pCPM_other	median_roundedDecontX_7gene_log1pCPM_other	pct_any_roundedDecontX_target_other	delta_mean_raw_7gene_counts	delta_mean_raw_7gene_log1pCPM	delta_pct_any_raw_target
JT06	0.311151079137	0.2500	0	0.6902	0	0.145683453237	-0.4756	-0.9128	-0.2195

Donor	pct_any_raw_target_other	mean_roundedDecontX_7gene_counts_other	median_roundedDecontX_7gene_counts_other	mean_roundedDecontX_7gene_log1pCPM_other	median_roundedDecontX_7gene_log1pCPM_other	pct_any_roundedDecontX_target_other	delta_mean_raw_7gene_counts	delta_mean_raw_7gene_log1pCPM	delta_pct_any_raw_target
NPC05	0.2944	0.2320	0	0.6716	0	0.144	-0.4224	-1.1291	-0.2604
WHN03	0.309789343247	0.2924	0	0.8778	0	0.175960346964	-0.3929	-1.0911	-0.2500
XZH02	0.650432900433	0.6190	0	1.5641	0	0.332251082251	-1.4589	-2.2053	-0.4816
ZWB04	0.284210526316	0.1987	0	0.5680	0	0.111842105263	-0.4576	-1.1149	-0.2390

Part 4 of 4 (source columns 1 and 29–31; identifier columns repeated)

Donor	delta_mean_roundedDecontX_7gene_counts	delta_mean_roundedDecontX_7gene_log1pCPM	delta_pct_any_roundedDecontX_target
JT06	-0.2118	-0.4307	-0.1075
NPC05	-0.1912	-0.4857	-0.1168
WHN03	-0.2327	-0.5957	-0.1361
XZH02	-0.5671	-1.2993	-0.2933
ZWB04	-0.1600	-0.3387	-0.0796

Library complexity

Part 1 of 2 (source columns 1 and 2–10; identifier columns repeated)

Donor	group_candidate	n_cells_candidate	mean_raw_UMI_candidate	median_raw_UMI_candidate	mean_detected_features_candidate	median_detected_features_candidate	group_other	n_cells_other	mean_raw_UMI_other
JT06	candidate	262	946.7977	777	504.1107	448.5000	other_myeloid	556	7858.7500
NPC05	candidate	147	1281.0476	882	620.7415	494	other_myeloid	625	10857.6832
WHN03	candidate	251	1001.4980	840	529.8088	470	other_myeloid	807	8391.6258
XZH02	candidate	77	1267.1169	936	570.5455	485	other_myeloid	924	11714.3203
ZWB04	candidate	155	1027.3032	805	538.2645	460	other_myeloid	760	7258.2276

Part 2 of 2 (source columns 1 and 11–15; identifier columns repeated)

Donor	median_raw_UMI_other	mean_detected_features_other	median_detected_features_other	mean_UMI_ratio	mean_feature_ratio
JT06	2156.5000	1940.1547	1105	0.1205	0.2598

Donor	median_raw_UMI_other	mean_detected_features_other	median_detected_features_other	mean_UMI_ratio	mean_feature_ratio
NPC05	10613	2577.8336	2926	0.1180	0.2408
WHN03	8086	2224.0235	2538	0.1193	0.2382
XZH02	12054	2653.5076	3010	0.1082	0.2150
ZWB04	5366.5000	1872.8697	1857	0.1415	0.2874

Corrected target-gene direction summary

Target	median_delta_raw_detection	donors_higher_raw_detection	donors_lower_raw_detection	median_delta_rounded_detection	donors_higher_rounded_detection	donors_lower_rounded_detection	median_delta_raw_count_per_cell	donors_higher_raw_count_per_cell	donors_lower_raw_count_per_cell
CCN2_or_CTGF	-0.0589	0	5	0	2	1	-0.0627	0	5
COL1A1	-0.0756	0	5	0	2	1	-0.0804	0	5
COL1A2	-0.0700	0	5	0	2	0	-0.0828	0	5
COL5A1	-0.1204	0	5	-0.1204	0	5	-0.2237	0	5
FBLN2	-0.0192	0	5	0	1	0	-0.0192	0	5
LUM	-0.0018	0	4	0	0	1	-0.0018	0	4
THBS2	0	0	2	0	0	2	0	0	2

UCell robustness summary

score_variant	n_donors	median_delta_mean	donors_positive_mean	median_delta_median	donors_positive_median	same_direction_rule
decontX_unrounded_maxRank1500_PRIMARY	5	0.0024	4	0	0	TRUE
raw_maxRank1000	5	0.0027	4	0	0	TRUE
raw_maxRank1500	5	0.0016	4	0	0	TRUE
raw_maxRank500	5	0.0024	5	0	0	TRUE
roundedDecontX_maxRank1000	5	0.0011	4	0	0	TRUE
roundedDecontX_maxRank1500	5	-0.0006	2	0	0	FALSE
roundedDecontX_maxRank500	5	0.0011	4	0	0	TRUE

Leave-one-gene-out summary

matrix_source	omitted_gene	median_delta_mean	donors_positive_ mean	same_direction_ rule
raw	COL1A1	0.0003	4	TRUE
roundedDecontX	COL1A1	-0.0006	2	FALSE
raw	COL1A2	0.0006	3	FALSE
roundedDecontX	COL1A2	-0.0007	2	FALSE
raw	COL5A1	0.0016	4	TRUE
roundedDecontX	COL5A1	0.0009	3	FALSE
raw	LUM	0.0019	4	TRUE
roundedDecontX	LUM	-0.0007	2	FALSE
raw	THBS2	0.0019	4	TRUE
roundedDecontX	THBS2	-0.0007	2	FALSE
raw	FBLN2	0.0020	4	TRUE
roundedDecontX	FBLN2	-0.0007	2	FALSE
raw	CCN2_or_CTGF	0.0017	4	TRUE
roundedDecontX	CCN2_or_CTGF	-0.0004	2	FALSE

DecontX fractional residual audit

Target	donor	group	n_cells	raw_detected_ fraction	decontX_positive_ fraction	rounded_decontX_ detected_fraction	fractional_only_ fraction	decontX_gt_1e6_ fraction	decontX_gt_0.5_ fraction
COL1A1	JT06	candidate	262	0.0343511450382	0.0343511450382	0.0114503816794	0.0229007633588	0.0343511450382	0.0114503816794
COL1A1	JT06	other_myeloid	556	0.091726618705	0.091726618705	0.00539568345324	0.0863309352518	0.0215827338129	0.00539568345324
COL1A1	NPC05	candidate	147	0.0204081632653	0.0204081632653	0.0136054421769	0.00680272108844	0.0204081632653	0.0136054421769
COL1A1	NPC05	other_myeloid	625	0.096	0.096	0.0032	0.0928	0.0032	0.0032
COL1A1	WHN03	candidate	251	0.0119521912351	0.0119521912351	0	0.0119521912351	0.0119521912351	0
COL1A1	WHN03	other_myeloid	807	0.0433705080545	0.0433705080545	0.00123915737299	0.0421313506815	0.00619578686493	0.00123915737299
COL1A1	XZH02	candidate	77	0.103896103896	0.103896103896	0	0.103896103896	0	0
COL1A1	XZH02	other_myeloid	924	0.44696969697	0.44696969697	0	0.44696969697	0	0
COL1A1	ZWB04	candidate	155	0	0	0	0	0	0
COL1A1	ZWB04	other_myeloid	760	0.0802631578947	0.0802631578947	0	0.0802631578947	0	0
COL1A2	JT06	candidate	262	0.0229007633588	0.0229007633588	0	0.0229007633588	0	0

Target	donor	group	n_cells	raw_detected_ fraction	decontX_positive_ fraction	rounded_decontX_ detected_fraction	fractional_only_ fraction	decontX_gt_1e6_ fraction	decontX_gt_0.5_ fraction
COL1A2	JT06	other_myeloid	556	0.0701438848921	0.0701438848921	0	0.0701438848921	0	0
COL1A2	NPC05	candidate	147	0.00680272108844	0.00680272108844	0	0.00680272108844	0	0
COL1A2	NPC05	other_myeloid	625	0.0768	0.0768	0	0.0768	0	0
COL1A2	WHN03	candidate	251	0.00796812749004	0.00796812749004	0.00398406374502	0.00398406374502	0.00796812749004	0.00398406374502
COL1A2	WHN03	other_myeloid	807	0.0681536555143	0.0681536555143	0.00123915737299	0.0669144981413	0.0681536555143	0.00123915737299
COL1A2	XZH02	candidate	77	0.0519480519481	0.0519480519481	0.025974025974	0.025974025974	0.0519480519481	0.025974025974
COL1A2	XZH02	other_myeloid	924	0.161255411255	0.161255411255	0	0.161255411255	0.00324675324675	0
COL1A2	ZWB04	candidate	155	0.0129032258065	0.0129032258065	0	0.0129032258065	0	0
COL1A2	ZWB04	other_myeloid	760	0.119736842105	0.119736842105	0	0.119736842105	0.075	0
COL5A1	JT06	candidate	262	0.0190839694656	0.0190839694656	0.0190839694656	0	0.0190839694656	0.0190839694656
COL5A1	JT06	other_myeloid	556	0.138489208633	0.138489208633	0.138489208633	0	0.138489208633	0.138489208633
COL5A1	NPC05	candidate	147	0.0204081632653	0.0204081632653	0.0204081632653	0	0.0204081632653	0.0204081632653
COL5A1	NPC05	other_myeloid	625	0.1408	0.1408	0.1408	0	0.1408	0.1408
COL5A1	WHN03	candidate	251	0.0358565737052	0.0358565737052	0.0358565737052	0	0.0358565737052	0.0358565737052
COL5A1	WHN03	other_myeloid	807	0.171003717472	0.171003717472	0.171003717472	0	0.171003717472	0.171003717472
COL5A1	XZH02	candidate	77	0.0519480519481	0.0519480519481	0.012987012987	0.038961038961	0.0519480519481	0.012987012987
COL5A1	XZH02	other_myeloid	924	0.344155844156	0.344155844156	0.332251082251	0.0119047619048	0.338744588745	0.332251082251
COL5A1	ZWB04	candidate	155	0.0322580645161	0.0322580645161	0.0322580645161	0	0.0322580645161	0.0322580645161
COL5A1	ZWB04	other_myeloid	760	0.111842105263	0.111842105263	0.111842105263	0	0.111842105263	0.111842105263
LUM	JT06	candidate	262	0	0	0	0	0	0
LUM	JT06	other_myeloid	556	0.00179856115108	0.00179856115108	0.00179856115108	0	0.00179856115108	0.00179856115108
LUM	NPC05	candidate	147	0	0	0	0	0	0
LUM	NPC05	other_myeloid	625	0	0	0	0	0	0
LUM	WHN03	candidate	251	0	0	0	0	0	0
LUM	WHN03	other_myeloid	807	0.00247831474597	0.00247831474597	0	0.00247831474597	0	0
LUM	XZH02	candidate	77	0	0	0	0	0	0
LUM	XZH02	other_myeloid	924	0.0021645021645	0.0021645021645	0	0.0021645021645	0	0
LUM	ZWB04	candidate	155	0	0	0	0	0	0
LUM	ZWB04	other_myeloid	760	0.00131578947368	0.00131578947368	0	0.00131578947368	0	0
THBS2	JT06	candidate	262	0	0	0	0	0	0

Target	donor	group	n_cells	raw_detected_ fraction	decontX_positive_ fraction	rounded_decontX_ detected_fraction	fractional_only_ fraction	decontX_gt_1e6_ fraction	decontX_gt_0.5_ fraction
THBS2	JT06	other_myeloid	556	0	0	0	0	0	0
THBS2	NPC05	candidate	147	0	0	0	0	0	0
THBS2	NPC05	other_myeloid	625	0.0016	0.0016	0.0016	0	0.0016	0.0016
THBS2	WHN03	candidate	251	0	0	0	0	0	0
THBS2	WHN03	other_myeloid	807	0	0	0	0	0	0
THBS2	XZH02	candidate	77	0	0	0	0	0	0
THBS2	XZH02	other_myeloid	924	0.0021645021645	0.0021645021645	0.00108225108225	0.00108225108225	0.0021645021645	0.00108225108225
THBS2	ZWB04	candidate	155	0	0	0	0	0	0
THBS2	ZWB04	other_myeloid	760	0	0	0	0	0	0
FBLN2	JT06	candidate	262	0.0152671755725	0.0152671755725	0.00381679389313	0.0114503816794	0.0152671755725	0.00381679389313
FBLN2	JT06	other_myeloid	556	0.0611510791367	0.0611510791367	0	0.0611510791367	0.00719424460432	0
FBLN2	NPC05	candidate	147	0	0	0	0	0	0
FBLN2	NPC05	other_myeloid	625	0.0192	0.0192	0	0.0192	0	0
FBLN2	WHN03	candidate	251	0	0	0	0	0	0
FBLN2	WHN03	other_myeloid	807	0.00991325898389	0.00991325898389	0	0.00991325898389	0	0
FBLN2	XZH02	candidate	77	0	0	0	0	0	0
FBLN2	XZH02	other_myeloid	924	0.0400432900433	0.0400432900433	0	0.0400432900433	0.00324675324675	0
FBLN2	ZWB04	candidate	155	0	0	0	0	0	0
FBLN2	ZWB04	other_myeloid	760	0.0118421052632	0.0118421052632	0	0.0118421052632	0	0
CCN2_or_CTGF	JT06	candidate	262	0.00763358778626	0.00763358778626	0.00381679389313	0.00381679389313	0.00763358778626	0.00381679389313
CCN2_or_CTGF	JT06	other_myeloid	556	0.0755395683453	0.0755395683453	0.00179856115108	0.0737410071942	0.0143884892086	0.00179856115108
CCN2_or_CTGF	NPC05	candidate	147	0	0	0	0	0	0
CCN2_or_CTGF	NPC05	other_myeloid	625	0.0352	0.0352	0	0.0352	0	0
CCN2_or_CTGF	WHN03	candidate	251	0.00796812749004	0.00796812749004	0	0.00796812749004	0	0
CCN2_or_CTGF	WHN03	other_myeloid	807	0.0669144981413	0.0669144981413	0.00247831474597	0.0644361833953	0.00247831474597	0.00247831474597
CCN2_or_CTGF	XZH02	candidate	77	0.025974025974	0.025974025974	0.012987012987	0.012987012987	0.025974025974	0.012987012987
CCN2_or_CTGF	XZH02	other_myeloid	924	0.0865800865801	0.0865800865801	0	0.0865800865801	0.0021645021645	0
CCN2_or_CTGF	ZWB04	candidate	155	0	0	0	0	0	0
CCN2_or_CTGF	ZWB04	other_myeloid	760	0.0473684210526	0.0473684210526	0	0.0473684210526	0	0

Technical artefact metrics

Metric	Donor	mean_candidate	mean_other	delta_mean	median_candidate	median_other	delta_median
decontX_contamination	JT06	0.1258	0.1202	0.0056	0.0707	0.0613	0.0094
decontX_contamination	NPC05	0.0991	0.0920	0.0071	0.0356	0.0390	-0.0034
decontX_contamination	WHN03	0.1160	0.0643	0.0517	0.0526	0.0379	0.0147
decontX_contamination	XZH02	0.1021	0.1019	0.0001	0.0216	0.0383	-0.0167
decontX_contamination	ZWB04	0.1209	0.0827	0.0382	0.0964	0.0455	0.0508
scDblFinder.score	JT06	0.0073	0.0474	-0.0402	0.0001	0.0060	-0.0059
scDblFinder.score	NPC05	0.0023	0.0386	-0.0363	0.0021	0.0043	-0.0021
scDblFinder.score	WHN03	0.0024	0.0578	-0.0554	0.0008	0.0104	-0.0097
scDblFinder.score	XZH02	0.0009	0.0474	-0.0465	0.0006	0.0056	-0.0049
scDblFinder.score	ZWB04	0.0021	0.0250	-0.0229	0.0012	0.0028	-0.0016

Target-independent lineage panels

Part 1 of 2 (source columns 1, 2 and 3–10; identifier columns repeated)

score_variant	Donor	n_state	n_other	mean_state	mean_other	delta_mean	median_state	median_other	delta_median
raw_logCPM_myeloid	JT06	262	556	9.9338	9.4617	0.4721	9.9390	9.5186	0.4204
raw_logCPM_myeloid	NPC05	147	625	9.7858	9.3678	0.4180	9.7779	9.4004	0.3775
raw_logCPM_myeloid	WHN03	251	807	9.7504	9.2582	0.4923	9.7920	9.3679	0.4241
raw_logCPM_myeloid	XZH02	77	924	9.7488	9.0943	0.6545	9.8283	9.1810	0.6472
raw_logCPM_myeloid	ZWB04	155	760	9.7757	8.9988	0.7769	9.8292	9.0734	0.7558
raw_logCPM_stromal	JT06	262	556	0.5802	1.4045	-0.8243	0	0	0
raw_logCPM_stromal	NPC05	147	625	0.2569	0.9767	-0.7198	0	0	0
raw_logCPM_stromal	WHN03	251	807	0.3030	0.9006	-0.5976	0	0	0
raw_logCPM_stromal	XZH02	77	924	0.8787	2.8655	-1.9868	0	4.1189	-4.1189
raw_logCPM_stromal	ZWB04	155	760	0.2386	1.0732	-0.8347	0	0	0
raw_logCPM_RPE	JT06	262	556	0.9055	2.2253	-1.3198	0	0	0
raw_logCPM_RPE	NPC05	147	625	0.7076	2.3645	-1.6570	0	0	0
raw_logCPM_RPE	WHN03	251	807	0.9806	3.1657	-2.1851	0	4.4867	-4.4867
raw_logCPM_RPE	XZH02	77	924	0.9140	3.0313	-2.1173	0	4.2901	-4.2901
raw_logCPM_RPE	ZWB04	155	760	0.7679	2.1086	-1.3406	0	0	0

score_variant	Donor	n_state	n_other	mean_state	mean_other	delta_mean	median_state	median_other	delta_median
raw_logCPM_photoreceptor	JT06	262	556	0.7770	1.9452	-1.1682	0	0	0
raw_logCPM_photoreceptor	NPC05	147	625	1.1184	3.5676	-2.4492	0	4.8178	-4.8178
raw_logCPM_photoreceptor	WHN03	251	807	0.1783	1.0608	-0.8825	0	0	0
raw_logCPM_photoreceptor	XZH02	77	924	0.3423	1.9988	-1.6564	0	0	0
raw_logCPM_photoreceptor	ZWB04	155	760	1.1319	2.4012	-1.2693	0	0	0

Part 2 of 2 (source columns 1, 2 and 11–13; identifier columns repeated)

score_variant	Donor	pct_state_positive	pct_other_positive	Panel
raw_logCPM_myeloid	JT06	1	0.998201438849	myeloid
raw_logCPM_myeloid	NPC05	1	0.9984	myeloid
raw_logCPM_myeloid	WHN03	1	0.993804213135	myeloid
raw_logCPM_myeloid	XZH02	1	0.992424242424	myeloid
raw_logCPM_myeloid	ZWB04	1	0.992105263158	myeloid
raw_logCPM_stromal	JT06	0.0801526717557	0.285971223022	stromal
raw_logCPM_stromal	NPC05	0.0408163265306	0.2192	stromal
raw_logCPM_stromal	WHN03	0.0438247011952	0.189591078067	stromal
raw_logCPM_stromal	XZH02	0.12987012987	0.582251082251	stromal
raw_logCPM_stromal	ZWB04	0.0322580645161	0.221052631579	stromal
raw_logCPM_RPE	JT06	0.129770992366	0.415467625899	RPE
raw_logCPM_RPE	NPC05	0.102040816327	0.4784	RPE
raw_logCPM_RPE	WHN03	0.139442231076	0.589838909542	RPE
raw_logCPM_RPE	XZH02	0.12987012987	0.593073593074	RPE
raw_logCPM_RPE	ZWB04	0.109677419355	0.410526315789	RPE
raw_logCPM_photoreceptor	JT06	0.110687022901	0.381294964029	photoreceptor
raw_logCPM_photoreceptor	NPC05	0.163265306122	0.6608	photoreceptor
raw_logCPM_photoreceptor	WHN03	0.0278884462151	0.223048327138	photoreceptor
raw_logCPM_photoreceptor	XZH02	0.0519480519481	0.433982683983	photoreceptor

score_variant	Donor	pct_state_positive	pct_other_positive	Panel
raw_logCPM_photoreceptor	ZWB04	0.161290322581	0.456578947368	photoreceptor

Synthetic myeloid-stromal mixture challenge

Donor	Panel	Category	n	mean_score	median_score	q25	q75
JT06	Fibrosis7	candidate	262	0.6478	0	0	0
JT06	Fibrosis7	other_myeloid	556	1.5605	0	0	4.15355766329
JT06	Fibrosis7	synthetic_MM	300	2.5849	3.6979	0	4.72899403935
JT06	Fibrosis7	synthetic_MS	300	4.1428	5.0105	0	6.7161107216
JT06	Fibrosis7	stromal	497	3.9214	4.9017	0	7.35781007118
JT06	myeloid	candidate	262	9.9338	9.9390	9.64953985405	10.2590950722
JT06	myeloid	other_myeloid	556	9.4617	9.5186	9.160615434	9.84843830423
JT06	myeloid	synthetic_MM	300	9.5791	9.5869	9.27764191482	9.87037055454
JT06	myeloid	synthetic_MS	300	8.5967	8.8808	8.0056801862	9.34635291889
JT06	myeloid	stromal	497	2.0432	0	0	5.22679442496
JT06	stromal	candidate	262	0.5802	0	0	0
JT06	stromal	other_myeloid	556	1.4045	0	0	3.87733248821
JT06	stromal	synthetic_MM	300	2.3797	3.2521	0	4.58339242271
JT06	stromal	synthetic_MS	300	6.8298	7.1613	6.08967386902	7.92790422733
JT06	stromal	stromal	497	7.8868	8.2155	7.34729245544	9.07796534908
JT06	RPE	candidate	262	0.9055	0	0	0
JT06	RPE	other_myeloid	556	2.2253	0	0	5.02434608013
JT06	RPE	synthetic_MM	300	3.5843	4.7423	0	5.33291051959
JT06	RPE	synthetic_MS	300	2.6386	3.8008	0	5.23643690745
JT06	RPE	stromal	497	1.3945	0	0	3.49936668746
JT06	photoreceptor	candidate	262	0.7770	0	0	0
JT06	photoreceptor	other_myeloid	556	1.9452	0	0	4.55432827211
JT06	photoreceptor	synthetic_MM	300	2.6563	3.8344	0	4.79673256219
JT06	photoreceptor	synthetic_MS	300	2.6201	3.6979	0	4.96856497595
JT06	photoreceptor	stromal	497	1.6459	0	0	4.45709884153
NPC05	Fibrosis7	candidate	147	0.2413	0	0	0
NPC05	Fibrosis7	other_myeloid	625	1.3704	0	0	3.90709743362

Donor	Panel	Category	n	mean_score	median_score	q25	q75
NPC05	Fibrosis7	synthetic_MM	300	2.3091	3.2422	0	4.38092366834
NPC05	Fibrosis7	synthetic_MS	300	5.0542	5.7713	3.83777679408	7.2231182462
NPC05	Fibrosis7	stromal	98	5.5580	7.2391	0	8.55884254437
NPC05	myeloid	candidate	147	9.7858	9.7779	9.50814844462	10.1363403395
NPC05	myeloid	other_myeloid	625	9.3678	9.4004	9.07317112246	9.74704516424
NPC05	myeloid	synthetic_MM	300	9.4811	9.4900	9.23895805231	9.75110797343
NPC05	myeloid	synthetic_MS	300	8.5951	8.8546	8.14273784625	9.27475600431
NPC05	myeloid	stromal	98	3.2111	4.8517	0	5.63888320545
NPC05	stromal	candidate	147	0.2569	0	0	0
NPC05	stromal	other_myeloid	625	0.9767	0	0	0
NPC05	stromal	synthetic_MM	300	1.7626	0	0	3.86377901938
NPC05	stromal	synthetic_MS	300	6.6953	7.1250	5.86326973575	7.90424805318
NPC05	stromal	stromal	98	7.9408	8.1514	7.79507388788	8.80190589777
NPC05	RPE	candidate	147	0.7076	0	0	0
NPC05	RPE	other_myeloid	625	2.3645	0	0	4.84882427495
NPC05	RPE	synthetic_MM	300	3.7696	4.4454	3.6824375709	5.07768486623
NPC05	RPE	synthetic_MS	300	2.7706	3.7979	0	4.75064298371
NPC05	RPE	stromal	98	1.0267	0	0	0
NPC05	photoreceptor	candidate	147	1.1184	0	0	0
NPC05	photoreceptor	other_myeloid	625	3.5676	4.8178	0	5.54456005847
NPC05	photoreceptor	synthetic_MM	300	4.5900	5.1276	4.58210374718	5.51545325629
NPC05	photoreceptor	synthetic_MS	300	4.3690	4.9780	4.37182213264	5.55086254603
NPC05	photoreceptor	stromal	98	2.7856	3.6068	0	5.29453968131
WHN03	Fibrosis7	candidate	251	0.4188	0	0	0
WHN03	Fibrosis7	other_myeloid	807	1.5099	0	0	4.25681182646
WHN03	Fibrosis7	synthetic_MM	300	2.3082	3.2888	0	4.54527550523
WHN03	Fibrosis7	synthetic_MS	300	5.8070	6.8684	4.29297085979	8.42023845587
WHN03	Fibrosis7	stromal	64	5.9908	7.8082	0	9.04151715488
WHN03	myeloid	candidate	251	9.7504	9.7920	9.48564322293	10.0725489077
WHN03	myeloid	other_myeloid	807	9.2582	9.3679	8.99050897061	9.68933497667
WHN03	myeloid	synthetic_MM	300	9.3188	9.3367	9.04403806774	9.60819175284

Donor	Panel	Category	n	mean_score	median_score	q25	q75
WHN03	myeloid	synthetic_MS	300	8.4469	8.6830	7.94477825133	9.21722862123
WHN03	myeloid	stromal	64	3.2558	4.9063	0	5.51513942592
WHN03	stromal	candidate	251	0.3030	0	0	0
WHN03	stromal	other_myeloid	807	0.9006	0	0	0
WHN03	stromal	synthetic_MM	300	1.4081	0	0	3.7531366626
WHN03	stromal	synthetic_MS	300	6.6378	7.2843	5.50982748382	8.64324720642
WHN03	stromal	stromal	64	7.4918	8.4765	7.14733073162	9.53721198003
WHN03	RPE	candidate	251	0.9806	0	0	0
WHN03	RPE	other_myeloid	807	3.1657	4.4867	0	5.4360246761
WHN03	RPE	synthetic_MM	300	4.4018	5.0648	4.44184353376	5.50552100671
WHN03	RPE	synthetic_MS	300	4.1397	4.9174	4.10077253273	5.46883276961
WHN03	RPE	stromal	64	2.9678	4.1465	0	5.38255153818
WHN03	photoreceptor	candidate	251	0.1783	0	0	0
WHN03	photoreceptor	other_myeloid	807	1.0608	0	0	0
WHN03	photoreceptor	synthetic_MM	300	1.8160	0	0	4.01778238315
WHN03	photoreceptor	synthetic_MS	300	1.3207	0	0	3.59719597319
WHN03	photoreceptor	stromal	64	0.5009	0	0	0
XZH02	Fibrosis7	candidate	77	1.1478	0	0	0
XZH02	Fibrosis7	other_myeloid	924	3.3531	4.5288	0	5.31421142009
XZH02	Fibrosis7	synthetic_MM	300	4.3800	4.8895	4.29003193818	5.3363925429
XZH02	Fibrosis7	synthetic_MS	300	6.8189	7.1376	5.24761607857	8.95172442239
XZH02	Fibrosis7	stromal	272	6.5510	7.8676	0	10.024718899
XZH02	myeloid	candidate	77	9.7488	9.8283	9.48081311292	10.0848756816
XZH02	myeloid	other_myeloid	924	9.0943	9.1810	8.81045251928	9.53841959287
XZH02	myeloid	synthetic_MM	300	9.2289	9.2082	8.94467334279	9.53736679741
XZH02	myeloid	synthetic_MS	300	8.3446	8.6359	7.75813373261	9.18884342945
XZH02	myeloid	stromal	272	3.1082	4.7175	0	5.58217571594
XZH02	stromal	candidate	77	0.8787	0	0	0
XZH02	stromal	other_myeloid	924	2.8655	4.1189	0	4.97237423215
XZH02	stromal	synthetic_MM	300	3.7702	4.4682	3.7593442117	4.93578808922
XZH02	stromal	synthetic_MS	300	7.3719	7.3565	6.24525221506	8.79435279127

Donor	Panel	Category	n	mean_score	median_score	q25	q75
XZH02	stromal	stromal	272	8.4660	8.8367	7.96090447399	9.5007462159
XZH02	RPE	candidate	77	0.9140	0	0	0
XZH02	RPE	other_myeloid	924	3.0313	4.2901	0	5.1600934474
XZH02	RPE	synthetic_MM	300	4.0552	4.7384	3.94503090208	5.20877515996
XZH02	RPE	synthetic_MS	300	3.2328	4.2445	0	4.94404715535
XZH02	RPE	stromal	272	1.7887	0	0	4.22587246067
XZH02	photoreceptor	candidate	77	0.3423	0	0	0
XZH02	photoreceptor	other_myeloid	924	1.9988	0	0	4.34383617972
XZH02	photoreceptor	synthetic_MM	300	2.7820	3.6905	0	4.41910693569
XZH02	photoreceptor	synthetic_MS	300	2.6723	3.7879	0	4.38731853801
XZH02	photoreceptor	stromal	272	1.5474	0	0	3.72395031631
ZWB04	Fibrosis7	candidate	155	0.3042	0	0	0
ZWB04	Fibrosis7	other_myeloid	760	1.4191	0	0	4.07547115702
ZWB04	Fibrosis7	synthetic_MM	300	2.2282	0	0	4.41515121778
ZWB04	Fibrosis7	synthetic_MS	300	5.2432	5.8736	0	8.13207824097
ZWB04	Fibrosis7	stromal	116	4.9369	6.5228	0	8.77785369124
ZWB04	myeloid	candidate	155	9.7757	9.8292	9.51205886242	10.0967402425
ZWB04	myeloid	other_myeloid	760	8.9988	9.0734	8.67376868326	9.50203294947
ZWB04	myeloid	synthetic_MM	300	9.1417	9.1169	8.84134097397	9.44148916789
ZWB04	myeloid	synthetic_MS	300	8.3119	8.4958	7.87124496801	8.91273255107
ZWB04	myeloid	stromal	116	1.7951	0	0	4.68192439323
ZWB04	stromal	candidate	155	0.2386	0	0	0
ZWB04	stromal	other_myeloid	760	1.0732	0	0	0
ZWB04	stromal	synthetic_MM	300	1.8076	0	0	4.14886511733
ZWB04	stromal	synthetic_MS	300	6.9221	7.1010	5.8907196182	8.40460168239
ZWB04	stromal	stromal	116	7.9362	8.2770	7.34426886728	9.39429526177
ZWB04	RPE	candidate	155	0.7679	0	0	0
ZWB04	RPE	other_myeloid	760	2.1086	0	0	4.86096286302
ZWB04	RPE	synthetic_MM	300	3.2873	4.4141	0	5.14558171033
ZWB04	RPE	synthetic_MS	300	3.1502	4.2090	0	5.13184383267
ZWB04	RPE	stromal	116	2.0955	0	0	4.74996784688

Donor	Panel	Category	n	mean_score	median_score	q25	q75
ZWB04	photoreceptor	candidate	155	1.1319	0	0	0
ZWB04	photoreceptor	other_myeloid	760	2.4012	0	0	4.96094653799
ZWB04	photoreceptor	synthetic_MM	300	3.5265	4.6489	0	5.15945954791
ZWB04	photoreceptor	synthetic_MS	300	3.4444	4.5569	0	5.08717031681
ZWB04	photoreceptor	stromal	116	2.0287	0	0	4.66269900237

Table S5. SCP2582 Independent Replication

LOCKED REPLICATION; SIX-GENE AMENDMENT BEFORE EXPRESSION VALUES

Exact seven-gene replication was not evaluable because CCN2/CTGF was absent. The amended assayable six-gene A+B+C+D test was evaluable and negative. Synthetic mixtures were not triggered because no state passed the primary gate.

Primary replication decision

Part 1 of 2 (source columns 1 and 2–10; identifier columns repeated)

Cluster	n_donors_present_ locked	A_raw_module_CPM_ positive_donors	A_pass	B_raw_any_target_ detection_positive_ donors	B_pass	C_number_of_ coherent_genes	C_pass	D_roundedDecontX_ module_CPM_ positive_donors	D_pass
0	5	5	TRUE	4	TRUE	1	FALSE	2	FALSE
1	5	3	FALSE	3	FALSE	1	FALSE	1	FALSE
2	5	1	FALSE	1	FALSE	0	FALSE	0	FALSE
3	4	1	FALSE	0	FALSE	0	FALSE	1	FALSE
4	4	2	FALSE	1	FALSE	0	FALSE	2	FALSE

Part 2 of 2 (source columns 1 and 11–13; identifier columns repeated)

Cluster	PRIMARY_ REPLICATION_PASS	EXACT_7_GENE_ REPLICATION_ EVALUABLE	MODIFIED_6_GENE_ REPLICATION_PASS
0	FALSE	FALSE	FALSE
1	FALSE	FALSE	FALSE
2	FALSE	FALSE	FALSE
3	FALSE	FALSE	FALSE
4	FALSE	FALSE	FALSE

Donor-level module effects

Part 1 of 2 (source columns 1, 2, 3 and 4–10; identifier columns repeated)

Cluster	Donor	present_locked	n_state	n_other	raw_module_CPM_ state	raw_module_CPM_ other	delta_raw_module_ CPM	raw_any_target_ detection_state	raw_any_target_ detection_other
0	PVR-22	TRUE	130	141	775.7013	766.2326	9.4687	1	1
0	PVR-24	TRUE	188	161	20.6027	13.8508	6.7519	0.159574468085	0.111801242236
0	PVR-25	TRUE	91	304	13.3098	11.4397	1.8701	0.175824175824	0.174342105263
0	PVR-29	TRUE	47	131	36.9300	24.1228	12.8071	0.36170212766	0.229007633588

Cluster	Donor	present_locked	n_state	n_other	raw_module_CPM_ state	raw_module_CPM_ other	delta_raw_module_ CPM	raw_any_target_ detection_state	raw_any_target_ detection_other
0	PVR-31	TRUE	40	48	6.4313	4.5557	1.8756	0.1	0.0833333333333
1	PVR-22	TRUE	53	218	786.2854	766.9208	19.3646	1	1
1	PVR-24	TRUE	50	299	17.3004	17.4172	-0.1168	0.14	0.137123745819
1	PVR-25	TRUE	65	330	14.9535	11.2758	3.6777	0.184615384615	0.172727272727
1	PVR-29	TRUE	47	131	21.7478	29.9921	-8.2443	0.212765957447	0.282442748092
1	PVR-31	TRUE	19	69	9.8156	4.1864	5.6292	0.157894736842	0.0724637681159
2	PVR-22	TRUE	44	227	742.5670	776.9253	-34.3584	1	1
2	PVR-24	TRUE	20	329	0	18.7307	-18.7307	0	0.145896656535
2	PVR-25	TRUE	80	315	14.4357	11.0441	3.3916	0.2375	0.15873015873
2	PVR-29	TRUE	25	153	20.2197	29.6245	-9.4048	0.24	0.267973856209
2	PVR-31	TRUE	10	78	0	6.2152	-6.2152	0	0.102564102564
3	PVR-22	TRUE	14	257	741.6374	772.3319	-30.6945	1	1
3	PVR-24	TRUE	21	328	6.7972	18.5357	-11.7384	0.0952380952381	0.140243902439
3	PVR-25	TRUE	63	332	6.8456	13.4635	-6.6180	0.174603174603	0.174698795181
3	PVR-29	TRUE	27	151	36.5965	26.9152	9.6813	0.259259259259	0.264900662252
3	PVR-31	FALSE	6	82	5.4271	5.3203	0.1068	0.166666666667	0.0853658536585
4	PVR-22	TRUE	14	257	714.0542	773.6100	-59.5558	1	1
4	PVR-24	TRUE	32	317	23.6751	16.9342	6.7409	0.125	0.13880126183
4	PVR-25	TRUE	37	358	6.0468	12.2924	-6.2457	0.0810810810811	0.184357541899
4	PVR-29	TRUE	16	162	35.0018	27.3932	7.6085	0.3125	0.259259259259
4	PVR-31	FALSE	6	82	0	5.5399	-5.5399	0	0.0975609756098

Part 2 of 2 (source columns 1, 2, 3 and 11–14; identifier columns repeated)

Cluster	Donor	present_locked	delta_raw_any_ target_detection	roundedDecontX_ module_CPM_state	roundedDecontX_ module_CPM_other	delta_ roundedDecontX_ module_CPM
0	PVR-22	TRUE	0	11.9174	4.1320	7.7854
0	PVR-24	TRUE	0.0478	5.0045	3.4780	1.5265
0	PVR-25	TRUE	0.0015	0	0	0
0	PVR-29	TRUE	0.1327	1.4919	3.9228	-2.4309

Cluster	Donor	present_locked	delta_raw_any_ target_detection	roundedDecontX_ module_CPM_state	roundedDecontX_ module_CPM_other	delta_ roundedDecontX_ module_CPM
0	PVR-31	TRUE	0.0167	0	0	0
1	PVR-22	TRUE	0	7.5526	7.8463	-0.2937
1	PVR-24	TRUE	0.0029	7.4732	3.7954	3.6778
1	PVR-25	TRUE	0.0119	0	0	0
1	PVR-29	TRUE	-0.0697	0	4.2435	-4.2435
1	PVR-31	TRUE	0.0854	0	0	0
2	PVR-22	TRUE	0	1.3616	9.1889	-7.8273
2	PVR-24	TRUE	-0.1459	0	4.6079	-4.6079
2	PVR-25	TRUE	0.0788	0	0	0
2	PVR-29	TRUE	-0.0280	0	3.8744	-3.8744
2	PVR-31	TRUE	-0.1026	0	0	0
3	PVR-22	TRUE	0	4.4120	7.9868	-3.5748
3	PVR-24	TRUE	-0.0450	0	4.7404	-4.7404
3	PVR-25	TRUE	-0.0001	0	0	0
3	PVR-29	TRUE	-0.0056	20.4049	1.0232	19.3818
3	PVR-31	FALSE	0.0813	0	0	0
4	PVR-22	TRUE	0	4.8600	7.9440	-3.0840
4	PVR-24	TRUE	-0.0138	4.7529	4.2466	0.5063
4	PVR-25	TRUE	-0.1033	0	0	0
4	PVR-29	TRUE	0.0532	5.8466	2.9575	2.8891
4	PVR-31	FALSE	-0.0976	0	0	0

Gene coherence summary

Cluster	Target	n_donors_present_ locked	donors_higher_raw_ CPM	donors_higher_raw_ detection	donors_both_higher	median_delta_raw_ CPM	median_delta_raw_ detection	donors_higher_ roundedDecontX_CPM	gene_coherent_ locked
0	COL1A1	5	5	4	4	1.6078	0.0124	0	TRUE
0	COL1A2	5	3	3	3	1.2075	0.0166	0	FALSE
0	COL5A1	5	4	3	3	1.0500	0.0022	2	FALSE
0	LUM	5	3	3	3	0.4197	0.0026	0	FALSE

Cluster	Target	n_donors_present_locked	donors_higher_raw_CPM	donors_higher_raw_detection	donors_both_higher	median_delta_raw_CPM	median_delta_raw_detection	donors_higher_roundedDecontX_CPM	gene_coherent_locked
0	THBS2	5	2	2	2	0	0	0	FALSE
0	FBLN2	5	1	1	1	-0.0682	-0.0009	2	FALSE
1	COL1A1	5	3	3	3	2.0207	0.0046	0	FALSE
1	COL1A2	5	3	3	3	3.1350	0.0100	0	FALSE
1	COL5A1	5	4	4	4	3.6281	0.0486	2	TRUE
1	LUM	5	1	1	1	-0.5456	-0.0145	0	FALSE
1	THBS2	5	2	2	2	0	0	0	FALSE
1	FBLN2	5	1	1	1	-0.1819	-0.0025	0	FALSE
2	COL1A1	5	1	1	1	-3.8875	-0.0227	0	FALSE
2	COL1A2	5	2	2	2	-3.8845	-0.0190	0	FALSE
2	COL5A1	5	1	2	1	-2.6931	-0.0128	0	FALSE
2	LUM	5	1	1	1	-0.7769	-0.0128	0	FALSE
2	THBS2	5	1	1	1	0	0	0	FALSE
2	FBLN2	5	1	1	1	-0.2008	-0.0032	0	FALSE
3	COL1A1	4	1	3	1	-1.6132	0.0138	1	FALSE
3	COL1A2	4	1	2	1	-3.1120	0.0028	0	FALSE
3	COL5A1	4	0	0	0	-3.6085	-0.0364	0	FALSE
3	LUM	4	1	2	1	-1.3155	0.0016	0	FALSE
3	THBS2	4	2	2	2	0.1040	0.0049	0	FALSE
3	FBLN2	4	1	1	1	-0.4670	-0.0046	1	FALSE
4	COL1A1	4	2	2	1	-1.4197	0.0018	1	FALSE
4	COL1A2	4	1	2	1	-2.4679	0.0036	0	FALSE
4	COL5A1	4	3	2	2	1.7612	0.0153	1	FALSE
4	LUM	4	0	0	0	-1.4874	-0.0152	0	FALSE
4	THBS2	4	0	0	0	-0.7332	-0.0104	0	FALSE
4	FBLN2	4	0	0	0	-0.8334	-0.0093	0	FALSE

Secondary FN1/SPARC

Target	Cluster	Donor	present_locked	delta_raw_CPM	delta_raw_ detection
FN1	0	PVR-22	TRUE	-1376.5511	0
FN1	0	PVR-24	TRUE	-134.8633	0.0282
FN1	0	PVR-25	TRUE	-1405.6698	0.0591
FN1	0	PVR-29	TRUE	-4.7721	0.0601
FN1	0	PVR-31	TRUE	-882.3384	-0.2500
FN1	1	PVR-22	TRUE	428.8275	0
FN1	1	PVR-24	TRUE	-112.3092	-0.1241
FN1	1	PVR-25	TRUE	-559.1932	0.0203
FN1	1	PVR-29	TRUE	30.0549	0.0312
FN1	1	PVR-31	TRUE	41.4629	0.0862
FN1	2	PVR-22	TRUE	2809.7061	0
FN1	2	PVR-24	TRUE	1068.2509	0.3964
FN1	2	PVR-25	TRUE	5651.1414	0.0984
FN1	2	PVR-29	TRUE	42.0976	0.0831
FN1	2	PVR-31	TRUE	1935.2843	0.4385
FN1	3	PVR-22	TRUE	-434.8804	0
FN1	3	PVR-24	TRUE	-138.5806	-0.1902
FN1	3	PVR-25	TRUE	-2292.2839	0.0934
FN1	3	PVR-29	TRUE	-38.1425	-0.0677
FN1	3	PVR-31	FALSE	-617.1993	0.3455
FN1	4	PVR-22	TRUE	-1506.2963	0
FN1	4	PVR-24	TRUE	-29.8920	-0.0084
FN1	4	PVR-25	TRUE	-380.9832	0.0270
FN1	4	PVR-29	TRUE	-43.6664	-0.1165
FN1	4	PVR-31	FALSE	-720.1917	-0.5488
SPARC	0	PVR-22	TRUE	-17.9279	0.0065
SPARC	0	PVR-24	TRUE	-4.8063	-0.0064
SPARC	0	PVR-25	TRUE	-40.8385	0.0199
SPARC	0	PVR-29	TRUE	-11.5795	0.1455

Target	Cluster	Donor	present_locked	delta_raw_CPM	delta_raw_ detection
SPARC	0	PVR-31	TRUE	-4.4225	-0.0750
SPARC	1	PVR-22	TRUE	35.2250	0.0138
SPARC	1	PVR-24	TRUE	14.1753	0.0594
SPARC	1	PVR-25	TRUE	-28.6026	-0.0343
SPARC	1	PVR-29	TRUE	57.5463	0.1166
SPARC	1	PVR-31	TRUE	-2.8980	-0.0069
SPARC	2	PVR-22	TRUE	42.8160	-0.0410
SPARC	2	PVR-24	TRUE	30.4360	0.1707
SPARC	2	PVR-25	TRUE	98.6076	0.1823
SPARC	2	PVR-29	TRUE	83.4523	0.1088
SPARC	2	PVR-31	TRUE	5.6246	0.0949
SPARC	3	PVR-22	TRUE	45.7938	0.0117
SPARC	3	PVR-24	TRUE	-0.0694	0.1535
SPARC	3	PVR-25	TRUE	7.8258	0.2318
SPARC	3	PVR-29	TRUE	23.0937	-0.0976
SPARC	3	PVR-31	FALSE	15.8417	0.3049
SPARC	4	PVR-22	TRUE	-58.3044	0.0117
SPARC	4	PVR-24	TRUE	-8.9308	-0.1394
SPARC	4	PVR-25	TRUE	-3.9536	0.0005
SPARC	4	PVR-29	TRUE	-66.9243	-0.0401
SPARC	4	PVR-31	FALSE	-0.8165	-0.0528

Secondary UCell

Cluster	Donor	present_locked	Source	mean_state	mean_other	delta_mean	median_state	median_other	delta_median
0	PVR-22	TRUE	raw	0.2167	0.2151	0.0016	0.2197	0.2257	-0.0060
0	PVR-22	TRUE	roundedDecontX	0.0004	0	0.0004	0	0	0
0	PVR-24	TRUE	raw	0.0020	0.0021	-0.0001	0	0	0
0	PVR-24	TRUE	roundedDecontX	0.0003	0.0004	-0.0001	0	0	0
0	PVR-25	TRUE	raw	0.0008	0.0016	-0.0008	0	0	0
0	PVR-25	TRUE	roundedDecontX	0	0	0	0	0	0

Cluster	Donor	present_locked	Source	mean_state	mean_other	delta_mean	median_state	median_other	delta_median
0	PVR-29	TRUE	raw	0.0040	0.0047	-0.0006	0	0	0
0	PVR-29	TRUE	roundedDecontX	0	0.0029	-0.0029	0	0	0
0	PVR-31	TRUE	raw	0	0	0	0	0	0
0	PVR-31	TRUE	roundedDecontX	0	0	0	0	0	0
1	PVR-22	TRUE	raw	0.2185	0.2152	0.0033	0.2295	0.2202	0.0093
1	PVR-22	TRUE	roundedDecontX	0	0.0002	-0.0002	0	0	0
1	PVR-24	TRUE	raw	0.0055	0.0015	0.0040	0	0	0
1	PVR-24	TRUE	roundedDecontX	0.0013	0.0002	0.0011	0	0	0
1	PVR-25	TRUE	raw	0.0020	0.0013	0.0006	0	0	0
1	PVR-25	TRUE	roundedDecontX	0	0	0	0	0	0
1	PVR-29	TRUE	raw	0.0000	0.0061	-0.0061	0	0	0
1	PVR-29	TRUE	roundedDecontX	0	0.0029	-0.0029	0	0	0
1	PVR-31	TRUE	raw	0	0	0	0	0	0
1	PVR-31	TRUE	roundedDecontX	0	0	0	0	0	0
2	PVR-22	TRUE	raw	0.2061	0.2178	-0.0116	0.2215	0.2221	-0.0006
2	PVR-22	TRUE	roundedDecontX	0	0.0002	-0.0002	0	0	0
2	PVR-24	TRUE	raw	0	0.0022	-0.0022	0	0	0
2	PVR-24	TRUE	roundedDecontX	0	0.0004	-0.0004	0	0	0
2	PVR-25	TRUE	raw	0.0024	0.0012	0.0013	0	0	0
2	PVR-25	TRUE	roundedDecontX	0	0	0	0	0	0
2	PVR-29	TRUE	raw	0.0015	0.0050	-0.0035	0	0	0
2	PVR-29	TRUE	roundedDecontX	0	0.0025	-0.0025	0	0	0
2	PVR-31	TRUE	raw	0	0	0	0	0	0
2	PVR-31	TRUE	roundedDecontX	0	0	0	0	0	0
3	PVR-22	TRUE	raw	0.2062	0.2164	-0.0102	0.2189	0.2226	-0.0038
3	PVR-22	TRUE	roundedDecontX	0	0.0002	-0.0002	0	0	0
3	PVR-24	TRUE	raw	0	0.0022	-0.0022	0	0	0
3	PVR-24	TRUE	roundedDecontX	0	0.0004	-0.0004	0	0	0
3	PVR-25	TRUE	raw	0	0.0017	-0.0017	0	0	0
3	PVR-25	TRUE	roundedDecontX	0	0	0	0	0	0
3	PVR-29	TRUE	raw	0.0135	0.0029	0.0105	0	0	0

Cluster	Donor	present_locked	Source	mean_state	mean_other	delta_mean	median_state	median_other	delta_median
3	PVR-29	TRUE	roundedDecontX	0.0118	0.0004	0.0114	0	0	0
3	PVR-31	FALSE	raw	0	0	0	0	0	0
3	PVR-31	FALSE	roundedDecontX	0	0	0	0	0	0
4	PVR-22	TRUE	raw	0.2054	0.2165	-0.0111	0.2090	0.2246	-0.0156
4	PVR-22	TRUE	roundedDecontX	0	0.0002	-0.0002	0	0	0
4	PVR-24	TRUE	raw	0	0.0022	-0.0022	0	0	0
4	PVR-24	TRUE	roundedDecontX	0	0.0004	-0.0004	0	0	0
4	PVR-25	TRUE	raw	0.0017	0.0014	0.0003	0	0	0
4	PVR-25	TRUE	roundedDecontX	0	0	0	0	0	0
4	PVR-29	TRUE	raw	0.0132	0.0036	0.0096	0	0	0
4	PVR-29	TRUE	roundedDecontX	0.0040	0.0020	0.0020	0	0	0
4	PVR-31	FALSE	raw	0	0	0	0	0	0
4	PVR-31	FALSE	roundedDecontX	0	0	0	0	0	0

Library complexity and technical artefacts

Part 1 of 2 (source columns 1, 2, 3 and 4–10; identifier columns repeated)

Cluster	Donor	present_locked	n_state	n_other	mean_raw_UMI_state	mean_raw_UMI_other	ratio_mean_raw_UMI	mean_detected_features_state	mean_detected_features_other
0	PVR-22	TRUE	130	141	15380.6231	16207.1418	0.9490	2885.2077	3062.0355
0	PVR-24	TRUE	188	161	8519.8457	8968.6770	0.9500	1968.6809	2141.9752
0	PVR-25	TRUE	91	304	14035.8022	16965.4046	0.8273	2828.5604	3149.2599
0	PVR-29	TRUE	47	131	14403.3404	12024.9618	1.1978	3029.4043	2660.4962
0	PVR-31	TRUE	40	48	15549.0750	18292.1458	0.8500	2677.0250	3105.7708
1	PVR-22	TRUE	53	218	15573.5849	15868.2936	0.9814	2991.1132	2973.8303
1	PVR-24	TRUE	50	299	8092.3000	8833.0201	0.9161	2004.8200	2055.9498
1	PVR-25	TRUE	65	330	14403.6308	16662.1364	0.8645	2894.0769	3111.0879
1	PVR-29	TRUE	47	131	11739.9787	12980.5191	0.9044	2862.6170	2720.3359
1	PVR-31	TRUE	19	69	16086.1053	17309.4203	0.9293	2909.1053	2911.3768
2	PVR-22	TRUE	44	227	17782.2955	15428.4890	1.1526	3240.1136	2926.2511
2	PVR-24	TRUE	20	329	10805.3500	8600.5502	1.2564	2331.2500	2031.4438
2	PVR-25	TRUE	80	315	18184.0750	15809.5714	1.1502	3467.1500	2975.8794

Cluster	Donor	present_locked	n_state	n_other	mean_raw_UMI_state	mean_raw_UMI_other	ratio_mean_raw_UMI	mean_detected_features_state	mean_detected_features_other
2	PVR-29	TRUE	25	153	15826.1600	12134.4641	1.3042	3174.8000	2689.7843
2	PVR-31	TRUE	10	78	21282.6000	16502.0513	1.2897	3576.5000	2825.5513
3	PVR-22	TRUE	14	257	16758.2857	15759.0350	1.0634	3023.6429	2974.6809
3	PVR-24	TRUE	21	328	14011.2857	8388.5701	1.6703	2564.4286	2015.6006
3	PVR-25	TRUE	63	332	25506.0317	14541.7500	1.7540	3792	2939.3916
3	PVR-29	TRUE	27	151	9108.3333	13286.7682	0.6855	2044.2593	2885.5099
3	PVR-31	FALSE	6	82	30710.1667	16045.4268	1.9140	4144.8333	2820.5976
4	PVR-22	TRUE	14	257	15204.9286	15843.6537	0.9597	3060.3571	2972.6809
4	PVR-24	TRUE	32	317	6599.7500	8941.6278	0.7381	2030.2188	2050.4826
4	PVR-25	TRUE	37	358	13408.9730	16588.2933	0.8083	2835.5946	3100.1592
4	PVR-29	TRUE	16	162	10713.7500	12844.4877	0.8341	2301.4375	2802.9877
4	PVR-31	FALSE	6	82	9321	17610.4878	0.5293	2035.5000	2974.9390

Part 2 of 2 (source columns 1, 2, 3 and 11–17; identifier columns repeated)

Cluster	Donor	present_locked	ratio_mean_detected_features	mean_decontX_contamination_state	mean_decontX_contamination_other	delta_decontX_contamination	mean_scDblFinder_score_state	mean_scDblFinder_score_other	delta_scDblFinder_score
0	PVR-22	TRUE	0.9423	0.049189	0.062671	-0.013482	0.0963	0.1209	-0.0245
0	PVR-24	TRUE	0.9191	0.004730	0.010401	-0.005670	0.0576	0.1209	-0.0633
0	PVR-25	TRUE	0.8982	0.005122	0.018089	-0.012967	0.2534	0.2421	0.0113
0	PVR-29	TRUE	1.1387	0.015069	0.032221	-0.017152	0.2422	0.1821	0.0601
0	PVR-31	TRUE	0.8620	0.022200	0.030768	-0.008568	0.2404	0.2385	0.0019
1	PVR-22	TRUE	1.0058	0.053413	0.056882	-0.003470	0.1455	0.1003	0.0452
1	PVR-24	TRUE	0.9751	0.012153	0.006542	0.005610	0.0817	0.0877	-0.0059
1	PVR-25	TRUE	0.9302	0.004726	0.017145	-0.012419	0.2693	0.2399	0.0294
1	PVR-29	TRUE	1.0523	0.006388	0.035335	-0.028947	0.1664	0.2093	-0.0429
1	PVR-31	TRUE	0.9992	0.010343	0.031426	-0.021083	0.2394	0.2394	-0.0000
2	PVR-22	TRUE	1.1073	0.079144	0.051757	0.027387	0.1216	0.1067	0.0149
2	PVR-24	TRUE	1.1476	0.011149	0.007115	0.004034	0.1359	0.0838	0.0520
2	PVR-25	TRUE	1.1651	0.043165	0.007974	0.035191	0.2450	0.2447	0.0003
2	PVR-29	TRUE	1.1803	0.010876	0.030439	-0.019563	0.2692	0.1864	0.0828

Cluster	Donor	present_locked	ratio_mean_detected_features	mean_decontX_contamination_state	mean_decontX_contamination_other	delta_decontX_contamination	mean_scDblFinder_score_state	mean_scDblFinder_score_other	delta_scDblFinder_score
2	PVR-31	TRUE	1.2658	0.014951	0.028402	-0.013451	0.3504	0.2251	0.1253
3	PVR-22	TRUE	1.0165	0.047546	0.056675	-0.009129	0.1117	0.1090	0.0028
3	PVR-24	TRUE	1.2723	0.008532	0.007270	0.001262	0.1486	0.0829	0.0657
3	PVR-25	TRUE	1.2901	0.001462	0.017689	-0.016227	0.2990	0.2344	0.0646
3	PVR-29	TRUE	0.7085	0.012724	0.030368	-0.017644	0.0876	0.2178	-0.1302
3	PVR-31	FALSE	1.4695	0.003960	0.028550	-0.024590	0.1154	0.2484	-0.1330
4	PVR-22	TRUE	1.0295	0.048233	0.056638	-0.008405	0.0764	0.1109	-0.0344
4	PVR-24	TRUE	0.9901	0.012709	0.006805	0.005904	0.0101	0.0946	-0.0844
4	PVR-25	TRUE	0.9147	0.009722	0.015657	-0.005935	0.2332	0.2459	-0.0128
4	PVR-29	TRUE	0.8211	0.005075	0.029925	-0.024850	0.1113	0.2066	-0.0953
4	PVR-31	FALSE	0.6842	0.110383	0.020763	0.089620	0.1597	0.2452	-0.0855

Target-independent lineage burdens

Cluster	Donor	present_locked	Panel	mean_log1pCPM_state	mean_log1pCPM_other	delta_mean_log1pCPM
0	PVR-22	TRUE	myeloid	9.6648	9.7397	-0.0750
0	PVR-22	TRUE	stromal	6.1069	6.0244	0.0825
0	PVR-22	TRUE	RPE	3.0269	3.6150	-0.5880
0	PVR-22	TRUE	photoreceptor	0.1955	0.5111	-0.3156
0	PVR-24	TRUE	myeloid	9.1130	9.2347	-0.1216
0	PVR-24	TRUE	stromal	1.2372	1.4444	-0.2072
0	PVR-24	TRUE	RPE	1.3071	1.2126	0.0945
0	PVR-24	TRUE	photoreceptor	0.1006	0.1980	-0.0974
0	PVR-25	TRUE	myeloid	9.9906	9.7973	0.1933
0	PVR-25	TRUE	stromal	1.1207	0.8654	0.2553
0	PVR-25	TRUE	RPE	1.8291	2.5154	-0.6863
0	PVR-25	TRUE	photoreceptor	0.1783	0.4672	-0.2889
0	PVR-29	TRUE	myeloid	9.9887	9.7924	0.1963
0	PVR-29	TRUE	stromal	1.6593	1.4063	0.2529

Cluster	Donor	present_locked	Panel	mean_log1pCPM_ state	mean_log1pCPM_ other	delta_mean_ log1pCPM
0	PVR-29	TRUE	RPE	1.9406	1.6741	0.2665
0	PVR-29	TRUE	photoreceptor	0.1807	0.1716	0.0090
0	PVR-31	TRUE	myeloid	9.6440	9.5243	0.1197
0	PVR-31	TRUE	stromal	0.7123	1.3378	-0.6255
0	PVR-31	TRUE	RPE	1.1073	2.2938	-1.1865
0	PVR-31	TRUE	photoreceptor	0	0.3877	-0.3877
1	PVR-22	TRUE	myeloid	9.7629	9.6894	0.0735
1	PVR-22	TRUE	stromal	6.1775	6.0364	0.1410
1	PVR-22	TRUE	RPE	3.5568	3.2785	0.2783
1	PVR-22	TRUE	photoreceptor	0.4903	0.3280	0.1624
1	PVR-24	TRUE	myeloid	9.2352	9.1581	0.0771
1	PVR-24	TRUE	stromal	1.4725	1.3094	0.1631
1	PVR-24	TRUE	RPE	1.6995	1.1906	0.5089
1	PVR-24	TRUE	photoreceptor	0.0935	0.1543	-0.0608
1	PVR-25	TRUE	myeloid	9.7859	9.8528	-0.0669
1	PVR-25	TRUE	stromal	0.9441	0.9203	0.0238
1	PVR-25	TRUE	RPE	2.0801	2.4119	-0.3318
1	PVR-25	TRUE	photoreceptor	0.3311	0.4143	-0.0832
1	PVR-29	TRUE	myeloid	9.8923	9.8270	0.0653
1	PVR-29	TRUE	stromal	1.7606	1.3700	0.3907
1	PVR-29	TRUE	RPE	1.8442	1.7087	0.1355
1	PVR-29	TRUE	photoreceptor	0.0959	0.2021	-0.1062
1	PVR-31	TRUE	myeloid	9.4706	9.6085	-0.1379
1	PVR-31	TRUE	stromal	1.2943	0.9872	0.3071
1	PVR-31	TRUE	RPE	2.0647	1.6690	0.3956
1	PVR-31	TRUE	photoreceptor	0.2016	0.2142	-0.0126
2	PVR-22	TRUE	myeloid	9.8003	9.6851	0.1153
2	PVR-22	TRUE	stromal	5.8038	6.1144	-0.3106
2	PVR-22	TRUE	RPE	3.7424	3.2535	0.4888
2	PVR-22	TRUE	photoreceptor	0.6657	0.3004	0.3653

Cluster	Donor	present_locked	Panel	mean_log1pCPM_ state	mean_log1pCPM_ other	delta_mean_ log1pCPM
2	PVR-24	TRUE	myeloid	9.3858	9.1560	0.2299
2	PVR-24	TRUE	stromal	0.7740	1.3667	-0.5927
2	PVR-24	TRUE	RPE	1.1439	1.2708	-0.1269
2	PVR-24	TRUE	photoreceptor	0	0.1544	-0.1544
2	PVR-25	TRUE	myeloid	9.9327	9.8187	0.1141
2	PVR-25	TRUE	stromal	0.7676	0.9640	-0.1963
2	PVR-25	TRUE	RPE	3.2704	2.1253	1.1451
2	PVR-25	TRUE	photoreceptor	0.3473	0.4142	-0.0669
2	PVR-29	TRUE	myeloid	10.0019	9.8185	0.1834
2	PVR-29	TRUE	stromal	1.8772	1.4071	0.4701
2	PVR-29	TRUE	RPE	3.0485	1.5314	1.5171
2	PVR-29	TRUE	photoreceptor	0.1682	0.1750	-0.0068
2	PVR-31	TRUE	myeloid	9.7247	9.5600	0.1648
2	PVR-31	TRUE	stromal	1.3785	1.0118	0.3667
2	PVR-31	TRUE	RPE	2.7468	1.6272	1.1196
2	PVR-31	TRUE	photoreceptor	0.3744	0.1906	0.1838
3	PVR-22	TRUE	myeloid	9.7330	9.7022	0.0309
3	PVR-22	TRUE	stromal	6.1081	6.0616	0.0465
3	PVR-22	TRUE	RPE	4.3995	3.2748	1.1247
3	PVR-22	TRUE	photoreceptor	0.9026	0.3301	0.5725
3	PVR-24	TRUE	myeloid	9.6137	9.1407	0.4730
3	PVR-24	TRUE	stromal	1.8291	1.3010	0.5281
3	PVR-24	TRUE	RPE	1.9688	1.2184	0.7504
3	PVR-24	TRUE	photoreceptor	0.8531	0.1003	0.7528
3	PVR-25	TRUE	myeloid	9.7921	9.8512	-0.0591
3	PVR-25	TRUE	stromal	1.0880	0.8931	0.1949
3	PVR-25	TRUE	RPE	3.3750	2.1641	1.2109
3	PVR-25	TRUE	photoreceptor	1.1747	0.2537	0.9210
3	PVR-29	TRUE	myeloid	9.8535	9.8426	0.0109
3	PVR-29	TRUE	stromal	1.0896	1.5417	-0.4521

Cluster	Donor	present_locked	Panel	mean_log1pCPM_ state	mean_log1pCPM_ other	delta_mean_ log1pCPM
3	PVR-29	TRUE	RPE	0.8707	1.9007	-1.0300
3	PVR-29	TRUE	photoreceptor	0.1719	0.1744	-0.0025
3	PVR-31	FALSE	myeloid	9.5044	9.5841	-0.0797
3	PVR-31	FALSE	stromal	1.9317	0.9892	0.9424
3	PVR-31	FALSE	RPE	2.3897	1.7080	0.6818
3	PVR-31	FALSE	photoreceptor	0.5419	0.1873	0.3546
4	PVR-22	TRUE	myeloid	9.9590	9.6899	0.2692
4	PVR-22	TRUE	stromal	6.0087	6.0670	-0.0583
4	PVR-22	TRUE	RPE	3.2608	3.3368	-0.0760
4	PVR-22	TRUE	photoreceptor	0	0.3793	-0.3793
4	PVR-24	TRUE	myeloid	9.6374	9.1219	0.5156
4	PVR-24	TRUE	stromal	1.2890	1.3372	-0.0482
4	PVR-24	TRUE	RPE	0.8127	1.3091	-0.4963
4	PVR-24	TRUE	photoreceptor	0.1536	0.1447	0.0089
4	PVR-25	TRUE	myeloid	10.0031	9.8251	0.1779
4	PVR-25	TRUE	stromal	0.8173	0.9352	-0.1179
4	PVR-25	TRUE	RPE	2.0788	2.3860	-0.3072
4	PVR-25	TRUE	photoreceptor	0.3866	0.4021	-0.0155
4	PVR-29	TRUE	myeloid	9.8743	9.8412	0.0331
4	PVR-29	TRUE	stromal	0.5090	1.5683	-1.0594
4	PVR-29	TRUE	RPE	1.1643	1.8018	-0.6375
4	PVR-29	TRUE	photoreceptor	0.3045	0.1611	0.1434
4	PVR-31	FALSE	myeloid	9.9049	9.5548	0.3500
4	PVR-31	FALSE	stromal	0	1.1306	-1.1306
4	PVR-31	FALSE	RPE	1.4627	1.7758	-0.3132
4	PVR-31	FALSE	photoreceptor	0.6522	0.1792	0.4729

Conditional diagnostics status

Analysis	Status
Secondary UCell	RUN successfully; secondary only
Synthetic myeloid-stromal mixtures	NOT RUN because no cluster passed the primary A+B+C+D gate

Table S6. GSE165784 Direct-ECM Adjudication

LOCKED EXTERNAL ADJUDICATION

Every eligible frozen myeloid state was tested against the exact seven-slot ECM programme. Public PDR sample accessions are consistency units; donor independence is not asserted.

Primary direct-ECM decision

Cluster	n_samples_present_locked	A_raw_module_CPM_positive_samples	A_pass	B_raw_detection_positive_samples	B_pass	C_number_of_coherent_genes	C_pass	D_roundedDecontX_positive_samples	D_pass	PRIMARY_DIRECT_ECM_PASS
0	5	4	TRUE	2	FALSE	0	FALSE	0	FALSE	FALSE
1	5	0	FALSE	2	FALSE	0	FALSE	0	FALSE	FALSE
2	5	3	FALSE	1	FALSE	0	FALSE	0	FALSE	FALSE
3	5	1	FALSE	1	FALSE	0	FALSE	0	FALSE	FALSE
4	5	4	TRUE	3	FALSE	1	FALSE	0	FALSE	FALSE
5	5	5	TRUE	3	FALSE	0	FALSE	4	TRUE	FALSE
6	5	5	TRUE	4	TRUE	0	FALSE	0	FALSE	FALSE

Sample-level module effects

Part 1 of 2 (source columns 1, 2, 3 and 4–10; identifier columns repeated)

Cluster	public_unit	present_locked	n_state	n_other	raw_module_CPM_state	raw_module_CPM_other	delta_raw_module_CPM	raw_any_target_detection_state	raw_any_target_detection_other
0	GSM5049905	TRUE	139	772	111.0296	39.6088	71.4208	0.10071942446	0.0945595854922
0	GSM5049906	TRUE	266	797	6.5796	5.6076	0.9721	0.0300751879699	0.0250941028858
0	GSM5277737	TRUE	171	445	4.6442	5.1368	-0.4926	0.0116959064327	0.0157303370787
0	GSM5690478	TRUE	66	621	298.3511	263.2289	35.1221	0.712121212121	0.816425120773
0	GSM5690479	TRUE	406	1422	68.4446	65.2295	3.2151	0.307881773399	0.368495077356
1	GSM5049905	TRUE	176	735	17.8700	61.9384	-44.0685	0.0795454545455	0.0993197278912
1	GSM5049906	TRUE	174	889	2.0799	6.9230	-4.8431	0.0172413793103	0.0281214848144
1	GSM5277737	TRUE	147	469	1.4369	6.7259	-5.2890	0.00680272108844	0.0170575692964
1	GSM5690478	TRUE	161	526	252.8339	271.0769	-18.2429	0.863354037267	0.78897338403
1	GSM5690479	TRUE	306	1522	58.0243	68.4403	-10.4160	0.467320261438	0.332457293035
2	GSM5049905	TRUE	128	783	56.0613	49.2236	6.8377	0.1328125	0.0893997445722
2	GSM5049906	TRUE	118	945	5.3505	5.8423	-0.4918	0.0169491525424	0.0275132275132
2	GSM5277737	TRUE	71	545	0	5.4704	-5.4704	0	0.0165137614679

Cluster	public_unit	present_locked	n_state	n_other	raw_module_CPM_state	raw_module_CPM_other	delta_raw_module_CPM	raw_any_target_detection_state	raw_any_target_detection_other
2	GSM5690478	TRUE	60	627	276.9131	265.1239	11.7892	0.7	0.81658692185
2	GSM5690479	TRUE	304	1524	67.7982	65.5111	2.2871	0.299342105263	0.366141732283
3	GSM5049905	TRUE	131	780	32.3668	52.6526	-20.2859	0.0839694656489	0.0974358974359
3	GSM5049906	TRUE	179	884	3.0431	6.3179	-3.2747	0.0167597765363	0.0282805429864
3	GSM5277737	TRUE	24	592	0	5.1570	-5.1570	0	0.0152027027027
3	GSM5690478	TRUE	203	484	253.9277	271.3005	-17.3728	0.822660098522	0.79958677686
3	GSM5690479	TRUE	136	1692	65.9438	65.7676	0.1761	0.316176470588	0.358156028369
4	GSM5049905	TRUE	92	819	20.4361	54.1828	-33.7466	0.0869565217391	0.0964590964591
4	GSM5049906	TRUE	79	984	7.7954	5.6180	2.1774	0.0253164556962	0.0264227642276
4	GSM5277737	TRUE	106	510	8.6031	4.0751	4.5280	0.0283018867925	0.0117647058824
4	GSM5690478	TRUE	101	586	266.4599	265.8813	0.5785	0.821782178218	0.803754266212
4	GSM5690479	TRUE	187	1641	67.0819	65.6074	1.4744	0.368983957219	0.353443022547
5	GSM5049905	TRUE	174	737	59.0001	48.8218	10.1783	0.0977011494253	0.0949796472185
5	GSM5049906	TRUE	108	955	21.5175	4.6625	16.8549	0.037037037037	0.0251308900524
5	GSM5277737	TRUE	50	566	16.8023	4.3611	12.4412	0.02	0.0141342756184
5	GSM5690478	TRUE	25	662	404.4153	262.6942	141.7211	0.72	0.809667673716
5	GSM5690479	TRUE	181	1647	91.5478	64.1984	27.3494	0.232044198895	0.368548876746
6	GSM5049905	TRUE	62	849	99.6344	45.5708	54.0636	0.0645161290323	0.0977620730271
6	GSM5049906	TRUE	65	998	6.6574	5.7307	0.9267	0.0461538461538	0.0250501002004
6	GSM5277737	TRUE	38	578	9.5128	4.5628	4.9500	0.0526315789474	0.0121107266436
6	GSM5690478	TRUE	60	627	282.7562	264.5896	18.1666	0.833333333333	0.803827751196
6	GSM5690479	TRUE	207	1621	69.5517	65.1280	4.4236	0.492753623188	0.337446020975

Part 2 of 2 (source columns 1, 2, 3 and 11–14; identifier columns repeated)

Cluster	public_unit	present_locked	delta_raw_any_target_detection	roundedDecontX_module_CPM_state	roundedDecontX_module_CPM_other	delta_roundedDecontX_module_CPM
0	GSM5049905	TRUE	0.0062	0	1.9932	-1.9932
0	GSM5049906	TRUE	0.0050	0	0	0
0	GSM5277737	TRUE	-0.0040	0	1.1453	-1.1453

Cluster	public_unit	present_locked	delta_raw_any_ target_detection	roundedDecontX_ module_CPM_state	roundedDecontX_ module_CPM_other	delta_ roundedDecontX_ module_CPM
0	GSM5690478	TRUE	-0.1043	0	6.0154	-6.0154
0	GSM5690479	TRUE	-0.0606	0	0.3446	-0.3446
1	GSM5049905	TRUE	-0.0198	0.7805	2.0602	-1.2797
1	GSM5049906	TRUE	-0.0109	0	0	0
1	GSM5277737	TRUE	-0.0103	0	1.3510	-1.3510
1	GSM5690478	TRUE	0.0744	0	7.7131	-7.7131
1	GSM5690479	TRUE	0.1349	0	0.3904	-0.3904
2	GSM5049905	TRUE	0.0434	0	1.9383	-1.9383
2	GSM5049906	TRUE	-0.0106	0	0	0
2	GSM5277737	TRUE	-0.0165	0	0.9978	-0.9978
2	GSM5690478	TRUE	-0.1166	0	5.9735	-5.9735
2	GSM5690479	TRUE	-0.0668	0	0.3271	-0.3271
3	GSM5049905	TRUE	-0.0135	0	1.9613	-1.9613
3	GSM5049906	TRUE	-0.0115	0	0	0
3	GSM5277737	TRUE	-0.0152	0	0.9402	-0.9402
3	GSM5690478	TRUE	0.0231	0	7.9984	-7.9984
3	GSM5690479	TRUE	-0.0420	0	0.3053	-0.3053
4	GSM5049905	TRUE	-0.0095	0	1.9506	-1.9506
4	GSM5049906	TRUE	-0.0011	0	0	0
4	GSM5277737	TRUE	0.0165	0	1.1686	-1.1686
4	GSM5690478	TRUE	0.0180	0	6.4930	-6.4930
4	GSM5690479	TRUE	0.0155	0	0.3266	-0.3266
5	GSM5049905	TRUE	0.0027	12.7626	0.2421	12.5205
5	GSM5049906	TRUE	0.0119	0	0	0
5	GSM5277737	TRUE	0.0059	17.0436	0	17.0436
5	GSM5690478	TRUE	-0.0897	240.1249	0	240.1249
5	GSM5690479	TRUE	-0.1365	5.1568	0	5.1568
6	GSM5049905	TRUE	-0.0332	0	1.8635	-1.8635
6	GSM5049906	TRUE	0.0211	0	0	0

Cluster	public_unit	present_locked	delta_raw_any_ target_detection	roundedDecontX_ module_CPM_state	roundedDecontX_ module_CPM_other	delta_ roundedDecontX_ module_CPM
6	GSM5277737	TRUE	0.0405	0	1.0172	-1.0172
6	GSM5690478	TRUE	0.0295	0	6.0034	-6.0034
6	GSM5690479	TRUE	0.1553	0	0.3393	-0.3393

Gene coherence summary

Cluster	target	n_samples_pres ent_ locked	samples_higher_ raw_ CPM	samples_higher_ra w_ detection	samples_both_ higher	median_delta_ra w_ CPM	median_delta_raw _ detection	samples_higher_ roundedDecontX_CP M	gene_coherent_ locked
0	COL1A1	5	3	1	1	0.6098	-0.0088	0	FALSE
0	COL1A2	5	3	1	1	0.2216	-0.0022	0	FALSE
0	COL5A1	5	4	3	3	1.6663	0.0059	0	FALSE
0	LUM	5	1	1	1	0	0	0	FALSE
0	THBS2	5	3	3	3	1.8321	0.0058	0	FALSE
0	FBLN2	5	1	0	0	0	-0.0026	0	FALSE
0	CCN2_or_CTGF	5	4	2	2	1.2657	-0.0022	0	FALSE
1	COL1A1	5	0	3	0	-2.6904	0.0082	0	FALSE
1	COL1A2	5	1	3	1	-4.6679	0.0068	0	FALSE
1	COL5A1	5	0	2	0	-1.6864	-0.0027	0	FALSE
1	LUM	5	0	0	0	-0.2098	-0.0007	0	FALSE
1	THBS2	5	1	2	1	-0.4196	-0.0011	1	FALSE
1	FBLN2	5	1	2	1	0	0	0	FALSE
1	CCN2_or_CTGF	5	1	2	1	-0.7508	-0.0021	0	FALSE
2	COL1A1	5	2	1	1	-0.3410	-0.0073	0	FALSE
2	COL1A2	5	2	1	0	-0.4973	-0.0042	0	FALSE
2	COL5A1	5	1	1	1	-1.2393	-0.0037	0	FALSE
2	LUM	5	2	1	1	0	0	0	FALSE
2	THBS2	5	0	0	0	-1.2973	-0.0089	0	FALSE
2	FBLN2	5	0	0	0	-0.5914	-0.0026	0	FALSE
2	CCN2_or_CTGF	5	2	1	1	-0.3317	-0.0018	0	FALSE
3	COL1A1	5	0	0	0	-2.4889	-0.0124	0	FALSE

Cluster	target	n_samples_present_locked	samples_higher_raw_CPM	samples_higher_raw_detection	samples_both_higher	median_delta_raw_CPM	median_delta_raw_detection	samples_higher_roundedDecontX_CPM	gene_coherent_locked
3	COL1A2	5	2	2	2	-0.4688	-0.0004	0	FALSE
3	COL5A1	5	2	2	2	-1.3402	-0.0034	0	FALSE
3	LUM	5	2	1	1	0	0	0	FALSE
3	THBS2	5	1	1	0	-0.3829	-0.0011	0	FALSE
3	FBLN2	5	2	2	2	0	0	0	FALSE
3	CCN2_or_CTGF	5	1	1	1	-1.7231	-0.0090	0	FALSE
4	COL1A1	5	4	4	4	3.1156	0.0036	0	TRUE
4	COL1A2	5	2	2	2	-0.5822	-0.0020	0	FALSE
4	COL5A1	5	2	2	2	-0.4795	-0.0024	0	FALSE
4	LUM	5	0	0	0	-0.1756	-0.0010	0	FALSE
4	THBS2	5	2	2	2	-0.3511	-0.0010	0	FALSE
4	FBLN2	5	1	1	1	0	0	0	FALSE
4	CCN2_or_CTGF	5	2	2	2	-1.1643	-0.0020	0	FALSE
5	COL1A1	5	4	2	2	9.4353	-0.0071	2	FALSE
5	COL1A2	5	3	1	1	1.8728	-0.0086	1	FALSE
5	COL5A1	5	3	2	2	1.3547	-0.0027	2	FALSE
5	LUM	5	2	1	1	0	0	0	FALSE
5	THBS2	5	3	2	2	1.1942	-0.0018	0	FALSE
5	FBLN2	5	2	1	1	0	0	2	FALSE
5	CCN2_or_CTGF	5	4	3	3	6.1364	0.0122	2	FALSE
6	COL1A1	5	4	4	3	8.4988	0.0112	0	FALSE
6	COL1A2	5	3	2	2	3.2381	-0.0017	0	FALSE
6	COL5A1	5	1	1	1	-1.5209	-0.0035	0	FALSE
6	LUM	5	1	1	1	0	0	0	FALSE
6	THBS2	5	0	0	0	-0.9308	-0.0027	0	FALSE
6	FBLN2	5	2	2	2	0	0	0	FALSE
6	CCN2_or_CTGF	5	1	1	1	-1.5629	-0.0036	0	FALSE

Secondary FN1/SPARC

Target	Cluster	public_unit	present_locked	delta_raw_CPM	delta_raw_ detection
FN1	0	GSM5049905	TRUE	-1606.6601	0.1612
FN1	0	GSM5049906	TRUE	198.1185	0.2530
FN1	0	GSM5277737	TRUE	-80.1708	0.0168
FN1	0	GSM5690478	TRUE	-835.3770	-0.1845
FN1	0	GSM5690479	TRUE	-184.2390	0.1992
FN1	1	GSM5049905	TRUE	-1420.0570	-0.0802
FN1	1	GSM5049906	TRUE	-121.9123	-0.1474
FN1	1	GSM5277737	TRUE	-264.8454	-0.0691
FN1	1	GSM5690478	TRUE	-650.4438	0.0421
FN1	1	GSM5690479	TRUE	-899.5641	0.0176
FN1	2	GSM5049905	TRUE	-2255.1150	-0.3339
FN1	2	GSM5049906	TRUE	-107.2341	-0.1682
FN1	2	GSM5277737	TRUE	-290.2789	-0.1644
FN1	2	GSM5690478	TRUE	-1081.4031	-0.1179
FN1	2	GSM5690479	TRUE	-696.5764	-0.2614
FN1	3	GSM5049905	TRUE	9469.4359	0.4466
FN1	3	GSM5049906	TRUE	-25.5655	-0.0425
FN1	3	GSM5277737	TRUE	-38.9712	0.0788
FN1	3	GSM5690478	TRUE	1070.6765	0.0865
FN1	3	GSM5690479	TRUE	-607.3695	-0.1942
FN1	4	GSM5049905	TRUE	-272.7831	0.2448
FN1	4	GSM5049906	TRUE	234.2891	0.2486
FN1	4	GSM5277737	TRUE	585.7099	0.2064
FN1	4	GSM5690478	TRUE	4.4783	-0.0041
FN1	4	GSM5690479	TRUE	3733.2143	0.1557
FN1	5	GSM5049905	TRUE	-2265.3978	-0.3596
FN1	5	GSM5049906	TRUE	-101.9918	-0.1845
FN1	5	GSM5277737	TRUE	-284.9158	-0.1914
FN1	5	GSM5690478	TRUE	-560.5299	-0.1290

Target	Cluster	public_unit	present_locked	delta_raw_CPM	delta_raw_ detection
FN1	5	GSM5690479	TRUE	-706.8247	-0.2947
FN1	6	GSM5049905	TRUE	-883.0180	0.1715
FN1	6	GSM5049906	TRUE	1.9771	0.0267
FN1	6	GSM5277737	TRUE	140.8868	0.1345
FN1	6	GSM5690478	TRUE	797.1356	0.0464
FN1	6	GSM5690479	TRUE	52.3492	0.2529
SPARC	0	GSM5049905	TRUE	1.5860	-0.0386
SPARC	0	GSM5049906	TRUE	-30.5100	-0.0943
SPARC	0	GSM5277737	TRUE	-10.0935	-0.0739
SPARC	0	GSM5690478	TRUE	-62.1564	-0.1701
SPARC	0	GSM5690479	TRUE	-130.7006	-0.2318
SPARC	1	GSM5049905	TRUE	0.7416	0.1407
SPARC	1	GSM5049906	TRUE	40.4007	0.2466
SPARC	1	GSM5277737	TRUE	32.8078	0.1561
SPARC	1	GSM5690478	TRUE	-14.3379	0.0947
SPARC	1	GSM5690479	TRUE	-40.8816	0.1148
SPARC	2	GSM5049905	TRUE	-18.1071	-0.0431
SPARC	2	GSM5049906	TRUE	-45.4800	-0.1195
SPARC	2	GSM5277737	TRUE	-28.6502	-0.0712
SPARC	2	GSM5690478	TRUE	-54.4912	-0.1751
SPARC	2	GSM5690479	TRUE	161.4044	0.1277
SPARC	3	GSM5049905	TRUE	-87.1885	-0.1739
SPARC	3	GSM5049906	TRUE	-64.1019	-0.1897
SPARC	3	GSM5277737	TRUE	-96.2212	-0.1993
SPARC	3	GSM5690478	TRUE	29.6762	0.0682
SPARC	3	GSM5690479	TRUE	-126.7830	-0.2259
SPARC	4	GSM5049905	TRUE	117.4325	0.3442
SPARC	4	GSM5049906	TRUE	63.8245	0.2494
SPARC	4	GSM5277737	TRUE	-10.3462	0.0848
SPARC	4	GSM5690478	TRUE	39.3428	0.0146

Target	Cluster	public_unit	present_locked	delta_raw_CPM	delta_raw_ detection
SPARC	4	GSM5690479	TRUE	96.9760	0.2995
SPARC	5	GSM5049905	TRUE	-74.0250	-0.2380
SPARC	5	GSM5049906	TRUE	-44.8475	-0.2193
SPARC	5	GSM5277737	TRUE	-145.5288	-0.3228
SPARC	5	GSM5690478	TRUE	-61.9484	-0.2489
SPARC	5	GSM5690479	TRUE	-161.7116	-0.4133
SPARC	6	GSM5049905	TRUE	90.8451	0.2275
SPARC	6	GSM5049906	TRUE	70.1106	0.2521
SPARC	6	GSM5277737	TRUE	66.0004	0.1955
SPARC	6	GSM5690478	TRUE	32.6614	0.0258
SPARC	6	GSM5690479	TRUE	144.7103	0.3442

Technical artefact diagnostics

Part 1 of 2 (source columns 1, 2, 3 and 4–10; identifier columns repeated)

Cluster	public_unit	present_locked	mean_raw_UMI_state	mean_raw_UMI_other	ratio_raw_UMI	mean_detected_ features_state	mean_detected_ features_other	ratio_detected_ features	mean_decontX_state
0	GSM5049905	TRUE	4989.2734	5265.2267	0.9476	1710.9281	1711.5622	0.9996	0.0526
0	GSM5049906	TRUE	4570.9586	6265.0590	0.7296	1496.1429	1820.1719	0.8220	0.0077
0	GSM5277737	TRUE	2518.3801	3937.2382	0.6396	1050.8830	1385.7888	0.7583	0.0148
0	GSM5690478	TRUE	6449.5909	8111.8196	0.7951	1847.2121	2259.5539	0.8175	0.0266
0	GSM5690479	TRUE	6045.6675	8376.7855	0.7217	1803.0813	2140.5443	0.8423	0.0937
1	GSM5049905	TRUE	7312.9261	4722.7061	1.5485	2113.6989	1615.1483	1.3087	0.0103
1	GSM5049906	TRUE	8289.5920	5361.9100	1.5460	2105.1839	1667.4342	1.2625	0.0012
1	GSM5277737	TRUE	4734.2041	3170.1194	1.4934	1571.4150	1205.4989	1.3035	0.0059
1	GSM5690478	TRUE	9507.1366	7476.1673	1.2717	2519.8820	2128.1331	1.1841	0.0193
1	GSM5690479	TRUE	11996.3203	7027.2378	1.7071	2621.0196	1953.9244	1.3414	0.0166
2	GSM5049905	TRUE	4320.0469	5370.7510	0.8044	1596.0781	1730.3282	0.9224	0.0071
2	GSM5049906	TRUE	4751.6695	5977.1746	0.7950	1609.1864	1755.3090	0.9168	0.0020
2	GSM5277737	TRUE	2420.7606	3689.6147	0.6561	1044.3944	1325.1835	0.7881	0.0169
2	GSM5690478	TRUE	6500.2333	8091.0670	0.8034	1872.4000	2253.1978	0.8310	0.0256

Cluster	public_unit	present_locked	mean_raw_UMI_state	mean_raw_UMI_other	ratio_raw_UMI	mean_detected_features_state	mean_detected_features_other	ratio_detected_features	mean_decontX_state
2	GSM5690479	TRUE	5531.1184	8323.4055	0.6645	1797.0066	2119.1699	0.8480	0.0279
3	GSM5049905	TRUE	4716.9313	5308.1359	0.8886	1642.9389	1722.9744	0.9535	0.0514
3	GSM5049906	TRUE	5507.4581	5908.7014	0.9321	1743.8324	1738.1278	1.0033	0.0049
3	GSM5277737	TRUE	2070.7500	3603.0676	0.5747	927.1667	1307.6436	0.7090	0.0634
3	GSM5690478	TRUE	8264.2512	7821.2190	1.0566	2331.7537	2173.0434	1.0730	0.0274
3	GSM5690479	TRUE	6690.1912	7952.9929	0.8412	1939.3235	2075.7429	0.9343	0.1718
4	GSM5049905	TRUE	6382.5543	5092.8803	1.2532	1960.1413	1683.5311	1.1643	0.0301
4	GSM5049906	TRUE	6495.2152	5788.6230	1.1221	1878.0380	1727.9329	1.0869	0.0036
4	GSM5277737	TRUE	4386.3113	3368.1667	1.3023	1491.2736	1251.5725	1.1915	0.0086
4	GSM5690478	TRUE	7877.3960	7965.0102	0.9890	2162.0099	2229.9249	0.9695	0.0243
4	GSM5690479	TRUE	8928.3476	7737.1901	1.1540	2221.3476	2047.8446	1.0847	0.0309
5	GSM5049905	TRUE	3214.4885	5697.3446	0.5642	1198.5632	1832.5577	0.6540	0.0325
5	GSM5049906	TRUE	3872.8241	6063.7298	0.6387	1398.4259	1777.6136	0.7867	0.0191
5	GSM5277737	TRUE	2380.6200	3646.0830	0.6529	1062.6400	1313.1537	0.8092	0.0279
5	GSM5690478	TRUE	5044.3200	8061.9411	0.6257	1632.9200	2242.1088	0.7283	0.0214
5	GSM5690479	TRUE	4586.5580	8218.6782	0.5581	1535.4586	2123.8537	0.7230	0.0842
6	GSM5049905	TRUE	6313.4032	5143.5018	1.2275	1957.8871	1693.4700	1.1561	0.0439
6	GSM5049906	TRUE	6932.7231	5770.0401	1.2015	1894.1692	1728.9880	1.0955	0.0048
6	GSM5277737	TRUE	5532.7105	3412.5796	1.6213	1729.4737	1264.1125	1.3681	0.0133
6	GSM5690478	TRUE	6896.4000	8053.1563	0.8564	2120.9167	2229.4163	0.9513	0.0203
6	GSM5690479	TRUE	10210.3237	7558.7866	1.3508	2419.8454	2020.3560	1.1977	0.0171

Part 2 of 2 (source columns 1, 2, 3 and 11–15; identifier columns repeated)

Cluster	public_unit	present_locked	mean_decontX_other	delta_decontX	mean_doublet_score_state	mean_doublet_score_other	delta_doublet_score
0	GSM5049905	TRUE	0.0268	0.0259	0.1073	0.1019	0.0054
0	GSM5049906	TRUE	0.0052	0.0025	0.1138	0.1406	-0.0268
0	GSM5277737	TRUE	0.0149	-0.0001	0.0901	0.1485	-0.0583
0	GSM5690478	TRUE	0.0236	0.0031	0.0984	0.1457	-0.0474
0	GSM5690479	TRUE	0.0479	0.0458	0.0702	0.0863	-0.0161

Cluster	public_unit	present_locked	mean_decontX_other	delta_decontX	mean_doublet_score_state	mean_doublet_score_other	delta_doublet_score
1	GSM5049905	TRUE	0.0356	-0.0253	0.1334	0.0953	0.0381
1	GSM5049906	TRUE	0.0067	-0.0055	0.1333	0.1340	-0.0007
1	GSM5277737	TRUE	0.0177	-0.0117	0.1772	0.1182	0.0590
1	GSM5690478	TRUE	0.0252	-0.0059	0.1766	0.1303	0.0463
1	GSM5690479	TRUE	0.0664	-0.0498	0.1147	0.0763	0.0384
2	GSM5049905	TRUE	0.0346	-0.0275	0.0975	0.1035	-0.0061
2	GSM5049906	TRUE	0.0063	-0.0042	0.1247	0.1351	-0.0104
2	GSM5277737	TRUE	0.0146	0.0023	0.0625	0.1414	-0.0789
2	GSM5690478	TRUE	0.0237	0.0019	0.0977	0.1453	-0.0477
2	GSM5690479	TRUE	0.0641	-0.0362	0.0579	0.0877	-0.0298
3	GSM5049905	TRUE	0.0273	0.0241	0.1249	0.0990	0.0259
3	GSM5049906	TRUE	0.0060	-0.0010	0.1590	0.1288	0.0302
3	GSM5277737	TRUE	0.0129	0.0505	0.0974	0.1337	-0.0363
3	GSM5690478	TRUE	0.0223	0.0051	0.1661	0.1308	0.0353
3	GSM5690479	TRUE	0.0489	0.1229	0.1008	0.0813	0.0195
4	GSM5049905	TRUE	0.0308	-0.0007	0.1363	0.0989	0.0374
4	GSM5049906	TRUE	0.0060	-0.0024	0.1885	0.1295	0.0589
4	GSM5277737	TRUE	0.0162	-0.0075	0.2079	0.1166	0.0913
4	GSM5690478	TRUE	0.0238	0.0005	0.1431	0.1408	0.0023
4	GSM5690479	TRUE	0.0611	-0.0302	0.1336	0.0769	0.0567
5	GSM5049905	TRUE	0.0303	0.0022	0.0385	0.1178	-0.0793
5	GSM5049906	TRUE	0.0043	0.0148	0.0696	0.1412	-0.0716
5	GSM5277737	TRUE	0.0137	0.0142	0.0534	0.1393	-0.0859
5	GSM5690478	TRUE	0.0239	-0.0026	0.0413	0.1450	-0.1037
5	GSM5690479	TRUE	0.0552	0.0290	0.0137	0.0903	-0.0766
6	GSM5049905	TRUE	0.0298	0.0141	0.0925	0.1034	-0.0110
6	GSM5049906	TRUE	0.0059	-0.0011	0.1982	0.1297	0.0685
6	GSM5277737	TRUE	0.0150	-0.0017	0.1880	0.1286	0.0594
6	GSM5690478	TRUE	0.0242	-0.0039	0.0911	0.1460	-0.0549
6	GSM5690479	TRUE	0.0633	-0.0461	0.0952	0.0811	0.0140

Target-independent lineage panels

Cluster	public_unit	present_locked	Panel	delta_mean_ log1pCPM
0	GSM5049905	TRUE	myeloid	-0.0123
0	GSM5049905	TRUE	stromal	0.2270
0	GSM5049906	TRUE	myeloid	-0.0501
0	GSM5049906	TRUE	stromal	0.0460
0	GSM5277737	TRUE	myeloid	0.0175
0	GSM5277737	TRUE	stromal	-0.1239
0	GSM5690478	TRUE	myeloid	0.0720
0	GSM5690478	TRUE	stromal	0.2089
0	GSM5690479	TRUE	myeloid	-0.0976
0	GSM5690479	TRUE	stromal	-0.5423
1	GSM5049905	TRUE	myeloid	0.1331
1	GSM5049905	TRUE	stromal	-0.0734
1	GSM5049906	TRUE	myeloid	0.1320
1	GSM5049906	TRUE	stromal	0.1891
1	GSM5277737	TRUE	myeloid	0.1129
1	GSM5277737	TRUE	stromal	0.0550
1	GSM5690478	TRUE	myeloid	0.1706
1	GSM5690478	TRUE	stromal	-0.0302
1	GSM5690479	TRUE	myeloid	0.2098
1	GSM5690479	TRUE	stromal	0.4988
2	GSM5049905	TRUE	myeloid	0.1551
2	GSM5049905	TRUE	stromal	0.0211
2	GSM5049906	TRUE	myeloid	0.1661
2	GSM5049906	TRUE	stromal	-0.0949
2	GSM5277737	TRUE	myeloid	0.0290
2	GSM5277737	TRUE	stromal	0.0658
2	GSM5690478	TRUE	myeloid	0.0097
2	GSM5690478	TRUE	stromal	0.4120

Cluster	public_unit	present_locked	Panel	delta_mean_ log1pCPM
2	GSM5690479	TRUE	myeloid	0.0091
2	GSM5690479	TRUE	stromal	-0.1184
3	GSM5049905	TRUE	myeloid	-0.1066
3	GSM5049905	TRUE	stromal	-0.1531
3	GSM5049906	TRUE	myeloid	-0.0901
3	GSM5049906	TRUE	stromal	-0.0967
3	GSM5277737	TRUE	myeloid	-0.2230
3	GSM5277737	TRUE	stromal	0.1943
3	GSM5690478	TRUE	myeloid	-0.1948
3	GSM5690478	TRUE	stromal	-0.2337
3	GSM5690479	TRUE	myeloid	-0.1259
3	GSM5690479	TRUE	stromal	-0.2358
4	GSM5049905	TRUE	myeloid	0.0306
4	GSM5049905	TRUE	stromal	-0.0015
4	GSM5049906	TRUE	myeloid	0.0670
4	GSM5049906	TRUE	stromal	0.0878
4	GSM5277737	TRUE	myeloid	0.1604
4	GSM5277737	TRUE	stromal	0.0258
4	GSM5690478	TRUE	myeloid	0.1051
4	GSM5690478	TRUE	stromal	-0.2100
4	GSM5690479	TRUE	myeloid	0.0658
4	GSM5690479	TRUE	stromal	0.1298
5	GSM5049905	TRUE	myeloid	0.0133
5	GSM5049905	TRUE	stromal	-0.0321
5	GSM5049906	TRUE	myeloid	-0.2284
5	GSM5049906	TRUE	stromal	-0.1097
5	GSM5277737	TRUE	myeloid	-0.5246
5	GSM5277737	TRUE	stromal	0.1572
5	GSM5690478	TRUE	myeloid	-0.4424
5	GSM5690478	TRUE	stromal	0.0529

Cluster	public_unit	present_locked	Panel	delta_mean_ log1pCPM
5	GSM5690479	TRUE	myeloid	-0.1800
5	GSM5690479	TRUE	stromal	-0.5667
6	GSM5049905	TRUE	myeloid	-0.5077
6	GSM5049905	TRUE	stromal	0.0148
6	GSM5049906	TRUE	myeloid	-0.1947
6	GSM5049906	TRUE	stromal	-0.0173
6	GSM5277737	TRUE	myeloid	-0.0156
6	GSM5277737	TRUE	stromal	-0.1754
6	GSM5690478	TRUE	myeloid	0.0570
6	GSM5690478	TRUE	stromal	0.2521
6	GSM5690479	TRUE	myeloid	-0.0523
6	GSM5690479	TRUE	stromal	0.6331

Synthetic myeloid-stromal mixtures

Cluster	public_unit	Category	n	mean_log1pCPM	median_log1pCPM	q25	q75
0	GSM5049905	state	139	0.6036	0	0	0
0	GSM5049905	other_myeloid	772	0.5420	0	0	0
0	GSM5049905	synthetic_MM	300	0.8907	0	0	0
0	GSM5049905	synthetic_MS	300	9.1092	9.1211	8.58206632083	9.81571977734
0	GSM5049905	stromal	71	9.6167	9.6752	9.09347211363	10.1602779061
0	GSM5049906	state	266	0.1638	0	0	0
0	GSM5049906	other_myeloid	797	0.1357	0	0	0
0	GSM5049906	synthetic_MM	300	0.3057	0	0	0
0	GSM5049906	synthetic_MS	300	8.2528	8.2233	7.59771268397	9.10102976999
0	GSM5049906	stromal	24	9.0857	9.1945	8.56844624942	9.75300648871
0	GSM5277737	state	171	0.0690	0	0	0
0	GSM5277737	other_myeloid	445	0.0925	0	0	0
0	GSM5277737	synthetic_MM	300	0.1766	0	0	0
0	GSM5277737	synthetic_MS	300	8.1830	8.4596	7.46293292473	9.03057945581
0	GSM5277737	stromal	110	8.9080	9.1059	8.35502626085	9.53867866152

Cluster	public_unit	Category	n	mean_log1pCPM	median_log1pCPM	q25	q75
0	GSM5690478	state	66	4.0593	5.2324	0	5.85128417723
0	GSM5690478	other_myeloid	621	4.5829	5.3723	4.73151171816	5.9173845712
0	GSM5690478	synthetic_MM	300	5.2884	5.4593	5.02850980638	5.90050942123
0	GSM5690478	synthetic_MS	300	8.8818	8.8535	8.42883706353	9.38826063128
0	GSM5690478	stromal	238	9.3184	9.2938	8.88528262938	9.82548058526
0	GSM5690479	state	406	1.6495	0	0	4.93785708818
0	GSM5690479	other_myeloid	1422	1.8376	0	0	4.66048632901
0	GSM5690479	synthetic_MM	300	2.8414	4.0603	0	4.59029284937
0	GSM5690479	synthetic_MS	300	7.8798	7.8944	7.00776315784	8.6537604532
0	GSM5690479	stromal	105	8.6293	8.6514	7.89687667527	9.36283929157
1	GSM5049905	state	176	0.4124	0	0	0
1	GSM5049905	other_myeloid	735	0.5846	0	0	0
1	GSM5049905	synthetic_MM	300	0.8651	0	0	0
1	GSM5049905	synthetic_MS	300	9.1140	9.0139	8.58278422718	9.79700964136
1	GSM5049905	stromal	71	9.6167	9.6752	9.09347211363	10.1602779061
1	GSM5049906	state	174	0.0806	0	0	0
1	GSM5049906	other_myeloid	889	0.1549	0	0	0
1	GSM5049906	synthetic_MM	300	0.2057	0	0	0
1	GSM5049906	synthetic_MS	300	8.3572	8.2858	7.835581244	9.11878951417
1	GSM5049906	stromal	24	9.0857	9.1945	8.56844624942	9.75300648871
1	GSM5277737	state	147	0.0382	0	0	0
1	GSM5277737	other_myeloid	469	0.1010	0	0	0
1	GSM5277737	synthetic_MM	300	0.1397	0	0	0
1	GSM5277737	synthetic_MS	300	8.1117	8.3836	7.49357023582	8.94742868761
1	GSM5277737	stromal	110	8.9080	9.1059	8.35502626085	9.53867866152
1	GSM5690478	state	161	4.7699	5.4143	4.74503721224	5.91941857868
1	GSM5690478	other_myeloid	526	4.4600	5.3502	4.65351472609	5.91547764128
1	GSM5690478	synthetic_MM	300	5.2627	5.4200	4.95707155977	5.86946912389
1	GSM5690478	synthetic_MS	300	8.9610	8.8964	8.48020450164	9.44707793159
1	GSM5690478	stromal	238	9.3184	9.2938	8.88528262938	9.82548058526
1	GSM5690479	state	306	2.1844	0	0	4.53257340089

Cluster	public_unit	Category	n	mean_log1pCPM	median_log1pCPM	q25	q75
1	GSM5690479	other_myeloid	1522	1.7177	0	0	4.78332840188
1	GSM5690479	synthetic_MM	300	2.3459	3.5935	0	4.55409480567
1	GSM5690479	synthetic_MS	300	7.9498	7.9930	7.22018435467	8.78448837212
1	GSM5690479	stromal	105	8.6293	8.6514	7.89687667527	9.36283929157
2	GSM5049905	state	128	0.7779	0	0	0
2	GSM5049905	other_myeloid	783	0.5143	0	0	0
2	GSM5049905	synthetic_MM	300	0.8380	0	0	0
2	GSM5049905	synthetic_MS	300	9.1263	9.1490	8.58511129473	9.76888422924
2	GSM5049905	stromal	71	9.6167	9.6752	9.09347211363	10.1602779061
2	GSM5049906	state	118	0.0981	0	0	0
2	GSM5049906	other_myeloid	945	0.1483	0	0	0
2	GSM5049906	synthetic_MM	300	0.2513	0	0	0
2	GSM5049906	synthetic_MS	300	8.3780	8.3324	7.72838164741	9.3418004577
2	GSM5049906	stromal	24	9.0857	9.1945	8.56844624942	9.75300648871
2	GSM5277737	state	71	0	0	0	0
2	GSM5277737	other_myeloid	545	0.0972	0	0	0
2	GSM5277737	synthetic_MM	300	0.1516	0	0	0
2	GSM5277737	synthetic_MS	300	8.1165	8.2896	7.40982763401	9.04926197469
2	GSM5277737	stromal	110	8.9080	9.1059	8.35502626085	9.53867866152
2	GSM5690478	state	60	4.0856	5.4144	0	6.09364917791
2	GSM5690478	other_myeloid	627	4.5754	5.3504	4.72961914981	5.91535474513
2	GSM5690478	synthetic_MM	300	5.1955	5.4490	4.93043135166	5.83301827901
2	GSM5690478	synthetic_MS	300	8.8764	8.8491	8.42850409405	9.47004130967
2	GSM5690478	stromal	238	9.3184	9.2938	8.88528262938	9.82548058526
2	GSM5690479	state	304	1.5979	0	0	4.79776668005
2	GSM5690479	other_myeloid	1524	1.8353	0	0	4.69520891791
2	GSM5690479	synthetic_MM	300	3.0010	4.1144	0	4.69601493565
2	GSM5690479	synthetic_MS	300	7.9588	7.8931	7.16350742983	8.76323064817
2	GSM5690479	stromal	105	8.6293	8.6514	7.89687667527	9.36283929157
3	GSM5049905	state	131	0.4902	0	0	0
3	GSM5049905	other_myeloid	780	0.5616	0	0	0

Cluster	public_unit	Category	n	mean_log1pCPM	median_log1pCPM	q25	q75
3	GSM5049905	synthetic_MM	300	0.9016	0	0	0
3	GSM5049905	synthetic_MS	300	8.9617	8.9837	8.43036504849	9.64788257906
3	GSM5049905	stromal	71	9.6167	9.6752	9.09347211363	10.1602779061
3	GSM5049906	state	179	0.0883	0	0	0
3	GSM5049906	other_myeloid	884	0.1537	0	0	0
3	GSM5049906	synthetic_MM	300	0.2177	0	0	0
3	GSM5049906	synthetic_MS	300	8.1352	8.1958	7.38881310395	8.76761533949
3	GSM5049906	stromal	24	9.0857	9.1945	8.56844624942	9.75300648871
3	GSM5277737	state	24	0	0	0	0
3	GSM5277737	other_myeloid	592	0.0895	0	0	0
3	GSM5277737	synthetic_MM	300	0.1743	0	0	0
3	GSM5277737	synthetic_MS	300	8.0861	8.3548	7.43572456145	8.95936081652
3	GSM5277737	stromal	110	8.9080	9.1059	8.35502626085	9.53867866152
3	GSM5690478	state	203	4.5809	5.3337	4.81799196629	5.8654422531
3	GSM5690478	other_myeloid	484	4.5123	5.3820	4.63345328479	5.95676526381
3	GSM5690478	synthetic_MM	300	5.3331	5.4823	4.93402880154	5.90197144796
3	GSM5690478	synthetic_MS	300	8.8878	8.8126	8.40604584578	9.41959224393
3	GSM5690478	stromal	238	9.3184	9.2938	8.88528262938	9.82548058526
3	GSM5690479	state	136	1.6518	0	0	4.89094810136
3	GSM5690479	other_myeloid	1692	1.8074	0	0	4.71726305002
3	GSM5690479	synthetic_MM	300	2.5859	3.9215	0	4.57640547327
3	GSM5690479	synthetic_MS	300	7.9491	7.9515	7.20061323267	8.911587422
3	GSM5690479	stromal	105	8.6293	8.6514	7.89687667527	9.36283929157
4	GSM5049905	state	92	0.4649	0	0	0
4	GSM5049905	other_myeloid	819	0.5611	0	0	0
4	GSM5049905	synthetic_MM	300	0.9452	0	0	0
4	GSM5049905	synthetic_MS	300	8.9363	8.9797	8.39883157296	9.75219478785
4	GSM5049905	stromal	71	9.6167	9.6752	9.09347211363	10.1602779061
4	GSM5049906	state	79	0.1430	0	0	0
4	GSM5049906	other_myeloid	984	0.1427	0	0	0
4	GSM5049906	synthetic_MM	300	0.3137	0	0	0

Cluster	public_unit	Category	n	mean_log1pCPM	median_log1pCPM	q25	q75
4	GSM5049906	synthetic_MS	300	8.2695	8.1989	7.67123211478	9.07049913699
4	GSM5049906	stromal	24	9.0857	9.1945	8.56844624942	9.75300648871
4	GSM5277737	state	106	0.1689	0	0	0
4	GSM5277737	other_myeloid	510	0.0688	0	0	0
4	GSM5277737	synthetic_MM	300	0.0871	0	0	0
4	GSM5277737	synthetic_MS	300	8.0522	8.2754	7.37915160648	8.96470223844
4	GSM5277737	stromal	110	8.9080	9.1059	8.35502626085	9.53867866152
4	GSM5690478	state	101	4.5750	5.2791	4.769757985	5.97687211991
4	GSM5690478	other_myeloid	586	4.5253	5.3854	4.68017611475	5.91019708327
4	GSM5690478	synthetic_MM	300	5.2918	5.4658	4.92352455603	5.8347034281
4	GSM5690478	synthetic_MS	300	8.8830	8.8642	8.38215572229	9.50209394925
4	GSM5690478	stromal	238	9.3184	9.2938	8.88528262938	9.82548058526
4	GSM5690479	state	187	1.8378	0	0	4.64502781144
4	GSM5690479	other_myeloid	1641	1.7910	0	0	4.7291179075
4	GSM5690479	synthetic_MM	300	2.9503	4.1427	0	4.67563789731
4	GSM5690479	synthetic_MS	300	8.0516	7.9793	7.25774066443	8.84437364962
4	GSM5690479	stromal	105	8.6293	8.6514	7.89687667527	9.36283929157
5	GSM5049905	state	174	0.5988	0	0	0
5	GSM5049905	other_myeloid	737	0.5402	0	0	0
5	GSM5049905	synthetic_MM	300	0.7699	0	0	0
5	GSM5049905	synthetic_MS	300	8.9642	9.0240	8.46514071097	9.6108435358
5	GSM5049905	stromal	71	9.6167	9.6752	9.09347211363	10.1602779061
5	GSM5049906	state	108	0.2281	0	0	0
5	GSM5049906	other_myeloid	955	0.1331	0	0	0
5	GSM5049906	synthetic_MM	300	0.2053	0	0	0
5	GSM5049906	synthetic_MS	300	8.2473	8.1877	7.54380699668	9.14632400268
5	GSM5049906	stromal	24	9.0857	9.1945	8.56844624942	9.75300648871
5	GSM5277737	state	50	0.1497	0	0	0
5	GSM5277737	other_myeloid	566	0.0804	0	0	0
5	GSM5277737	synthetic_MM	300	0.0289	0	0	0
5	GSM5277737	synthetic_MS	300	8.1619	8.2848	7.50321439186	8.96932773378

Cluster	public_unit	Category	n	mean_log1pCPM	median_log1pCPM	q25	q75
5	GSM5277737	stromal	110	8.9080	9.1059	8.35502626085	9.53867866152
5	GSM5690478	state	25	4.4443	5.6764	0	6.34988796158
5	GSM5690478	other_myeloid	662	4.5359	5.3480	4.70121675319	5.8923053427
5	GSM5690478	synthetic_MM	300	5.1676	5.3834	4.9456058492	5.80022714295
5	GSM5690478	synthetic_MS	300	8.8543	8.8509	8.37542554405	9.45704119464
5	GSM5690478	stromal	238	9.3184	9.2938	8.88528262938	9.82548058526
5	GSM5690479	state	181	1.2992	0	0	0
5	GSM5690479	other_myeloid	1647	1.8504	0	0	4.72623123696
5	GSM5690479	synthetic_MM	300	2.7752	3.9604	0	4.52974524128
5	GSM5690479	synthetic_MS	300	7.9404	7.8758	7.14637985182	8.59784598227
5	GSM5690479	stromal	105	8.6293	8.6514	7.89687667527	9.36283929157
6	GSM5049905	state	62	0.3955	0	0	0
6	GSM5049905	other_myeloid	849	0.5627	0	0	0
6	GSM5049905	synthetic_MM	300	0.8230	0	0	0
6	GSM5049905	synthetic_MS	300	8.9835	8.9775	8.43836570336	9.64842882339
6	GSM5049905	stromal	71	9.6167	9.6752	9.09347211363	10.1602779061
6	GSM5049906	state	65	0.2453	0	0	0
6	GSM5049906	other_myeloid	998	0.1360	0	0	0
6	GSM5049906	synthetic_MM	300	0.2583	0	0	0
6	GSM5049906	synthetic_MS	300	8.2290	8.2541	7.43911839549	9.13083881871
6	GSM5049906	stromal	24	9.0857	9.1945	8.56844624942	9.75300648871
6	GSM5277737	state	38	0.2678	0	0	0
6	GSM5277737	other_myeloid	578	0.0741	0	0	0
6	GSM5277737	synthetic_MM	300	0.1609	0	0	0
6	GSM5277737	synthetic_MS	300	8.1055	8.3538	7.36978100124	8.97954551913
6	GSM5277737	stromal	110	8.9080	9.1059	8.35502626085	9.53867866152
6	GSM5690478	state	60	4.7542	5.5095	4.79366058713	5.95735952324
6	GSM5690478	other_myeloid	627	4.5114	5.3500	4.68276179375	5.90553100605
6	GSM5690478	synthetic_MM	300	5.1590	5.4510	5.01169996905	5.84667735165
6	GSM5690478	synthetic_MS	300	8.8994	8.8691	8.46903221489	9.4112983151
6	GSM5690478	stromal	238	9.3184	9.2938	8.88528262938	9.82548058526

Cluster	public_unit	Category	n	mean_log1pCPM	median_log1pCPM	q25	q75
6	GSM5690479	state	207	2.3603	0	0	4.6808608316
6	GSM5690479	other_myeloid	1621	1.7237	0	0	4.72711826277
6	GSM5690479	synthetic_MM	300	2.8157	4.0768	0	4.78333489245
6	GSM5690479	synthetic_MS	300	7.8841	7.9071	7.14401172883	8.63019666276
6	GSM5690479	stromal	105	8.6293	8.6514	7.89687667527	9.36283929157

Table S7. GSE165784 Stromal Localisation

FROZEN COMPARTMENT ADJUDICATION

Stromal-minus-myeloid comparisons for the ECM7 module and each primary gene using raw CPM, raw detection, and rounded-DecontX CPM.

Sample-level localisation

public_unit	Feature	Role	myeloid_raw_CPM	stromal_raw_CPM	stromal_minus_myeloid_raw_CPM	myeloid_raw_detection	stromal_raw_detection	stromal_minus_myeloid_detection	myeloid_roundedDecontX_CPM	stromal_roundedDecontX_CPM	stromal_minus_myeloid_roundedDecontX_CPM
GSM5049905	ECM7_SUM	MODULE	50.0182	20078.1808	20028.1625	0.0954994511526	1	0.904500548847	1.7098	20148.8119	20147.1021
GSM5049905	COL1A1	GENE	25.6396	7940.5392	7914.8996	0.0537870472009	0.985915492958	0.932128445757	1.4961	7968.4724	7966.9764
GSM5049905	COL1A2	GENE	17.4433	8963.3742	8945.9308	0.0472008781559	1	0.952799121844	0	8994.9055	8994.9055
GSM5049905	COL5A1	GENE	0.4203	804.3518	803.9315	0.00219538968167	0.887323943662	0.88512855398	0	807.1814	807.1814
GSM5049905	LUM	GENE	1.2610	295.0218	293.7608	0.00439077936334	0.43661971831	0.432228938947	0	296.0596	296.0596
GSM5049905	THBS2	GENE	2.5219	243.5321	241.0102	0.00768386388584	0.478873239437	0.471189375551	0.2137	244.3888	244.1751
GSM5049905	FBLN2	GENE	0.6305	102.9793	102.3488	0.00219538968167	0.30985915493	0.307663765248	0	103.3416	103.3416
GSM5049905	CCN2_or_CTGF	GENE	2.1016	1728.3823	1726.2807	0.00878155872667	0.816901408451	0.808119849724	0	1734.4625	1734.4625
GSM5049906	ECM7_SUM	MODULE	5.7979	16026.1469	16020.3490	0.0263405456256	1	0.973659454374	0	16100.3680	16100.3680
GSM5049906	COL1A1	GENE	2.0937	5221.4857	5219.3920	0.0103480714958	0.875	0.864651928504	0	5245.6677	5245.6677
GSM5049906	COL1A2	GENE	0.6442	4616.4773	4615.8331	0.00376293508937	0.958333333333	0.954570398244	0	4637.8573	4637.8573
GSM5049906	COL5A1	GENE	1.1274	835.1747	834.0473	0.0065851364064	0.541666666667	0.53508153026	0	839.0426	839.0426
GSM5049906	LUM	GENE	0.1611	1039.0362	1038.8751	0.000940733772342	0.541666666667	0.540725932894	0	1043.8482	1043.8482
GSM5049906	THBS2	GENE	0.3221	282.7757	282.4536	0.000940733772342	0.375	0.374059266228	0	284.0853	284.0853

public_unit	Feature	Role	myeloid_raw_CPM	stromal_raw_CPM	stromal_minus_myeloid_raw_CPM	myeloid_raw_detection	stromal_raw_detection	stromal_minus_myeloid_detection	myeloid_roundedDecontX_CPM	stromal_roundedDecontX_CPM	stromal_minus_myeloid_roundedDecontX_CPM
GSM5049906	FBLN2	GENE	0	85.4903	85.4903	0	0.25	0.25	0	85.8862	85.8862
GSM5049906	CCN2_or_CTGF	GENE	1.4495	3945.7071	3944.2576	0.00752587017874	0.916666666667	0.909140796488	0	3963.9806	3963.9806
GSM5277737	ECM7_SUM	MODULE	5.0396	11418.4346	11413.3950	0.0146103896104	1	0.98538961039	0.9192	11445.3955	11444.4762
GSM5277737	COL1A1	GENE	1.8326	3060.3625	3058.5299	0.00649350649351	0.818181818182	0.811688311688	0	3067.5886	3067.5886
GSM5277737	COL1A2	GENE	0.4581	3646.8515	3646.3934	0.00162337662338	0.927272727273	0.925649350649	0	3655.4624	3655.4624
GSM5277737	COL5A1	GENE	1.3744	465.8123	464.4378	0.00324675324675	0.572727272727	0.569480519481	0	466.9121	466.9121
GSM5277737	LUM	GENE	0	1421.5722	1421.5722	0	0.736363636364	0.736363636364	0	1424.9288	1424.9288
GSM5277737	THBS2	GENE	0.4581	552.6995	552.2414	0.00162337662338	0.527272727273	0.525649350649	0	554.0046	554.0046
GSM5277737	FBLN2	GENE	0	159.2933	159.2933	0	0.345454545455	0.345454545455	0	159.6694	159.6694
GSM5277737	CCN2_or_CTGF	GENE	0.9163	2111.8432	2110.9269	0.00162337662338	0.727272727273	0.725649350649	0.9192	2116.8296	2115.9104
GSM5690478	ECM7_SUM	MODULE	265.9656	15163.4169	14897.4513	0.806404657933	1	0.193595342067	5.5475	15401.5329	15395.9854
GSM5690478	COL1A1	GENE	95.1838	6178.5933	6083.4095	0.4730713246	0.995798319328	0.522726994728	1.6642	6275.7253	6274.0611
GSM5690478	COL1A2	GENE	97.7465	4968.0881	4870.3416	0.497816593886	0.995798319328	0.497981725441	1.6642	5046.1901	5044.5258
GSM5690478	COL5A1	GENE	24.3451	1297.5826	1273.2375	0.154294032023	0.970588235294	0.816294203271	0.9246	1317.9815	1317.0569
GSM5690478	LUM	GENE	0	53.8060	53.8060	0	0.205882352941	0.205882352941	0	54.6519	54.6519
GSM5690478	THBS2	GENE	3.1118	144.2625	141.1507	0.0232896652111	0.504201680672	0.480912015461	0	146.5304	146.5304
GSM5690478	FBLN2	GENE	0.5491	35.6107	35.0616	0.00436681222707	0.235294117647	0.23092730542	0.1849	36.1706	35.9857

public_unit	Feature	Role	myeloid_raw_CPM	stromal_raw_CPM	stromal_minus_myeloid_raw_CPM	myeloid_raw_detection	stromal_raw_detection	stromal_minus_myeloid_detection	myeloid_roundedDecontX_CPM	stromal_roundedDecontX_CPM	stromal_minus_myeloid_roundedDecontX_CPM
GSM5690478	CCN2_or_CTGF	GENE	45.0293	2485.4737	2440.4444	0.25327510917	0.936974789916	0.683699680746	1.1095	2524.2831	2523.1736
GSM5690479	ECM7_SUM	MODULE	65.7788	12941.9420	12876.1632	0.355032822757	0.990476190476	0.635443367719	0.2880	13212.5617	13212.2737
GSM5690479	COL1A1	GENE	17.6106	4021.5184	4003.9078	0.112691466083	0.933333333333	0.82064186725	0	4105.7843	4105.7843
GSM5690479	COL1A2	GENE	19.0028	4826.6038	4807.6010	0.124726477024	0.971428571429	0.846702094405	0	4929.1368	4929.1368
GSM5690479	COL5A1	GENE	4.5245	547.1454	542.6209	0.0322757111597	0.771428571429	0.739152860269	0.1440	558.8817	558.7377
GSM5690479	LUM	GENE	2.2970	303.8611	301.5641	0.0169584245077	0.27619047619	0.259232051683	0	309.3809	309.3809
GSM5690479	THBS2	GENE	1.7402	348.8052	347.0650	0.0120350109409	0.47619047619	0.46415546525	0	356.2871	356.2871
GSM5690479	FBLN2	GENE	1.5314	198.3402	196.8088	0.0120350109409	0.361904761905	0.349869750964	0.1440	202.5946	202.4506
GSM5690479	CCN2_or_CTGF	GENE	19.0724	2695.6680	2676.5956	0.128555798687	0.809523809524	0.680968010837	0	2750.4963	2750.4963

Localisation summary

feature	role	samples_stromal_higher_raw_CPM	samples_stromal_higher_detection	samples_stromal_higher_roundedDecontX_CPM	median_stromal_minus_myeloid_raw_CPM
ECM7_SUM	MODULE	5	5	5	14897.4513
COL1A1	GENE	5	5	5	5219.3920
COL1A2	GENE	5	5	5	4807.6010
COL5A1	GENE	5	5	5	803.9315
LUM	GENE	5	5	5	301.5641
THBS2	GENE	5	5	5	282.4536
FBLN2	GENE	5	5	5	102.3488
CCN2_or_CTGF	GENE	5	5	5	2440.4444

Table S8. GSE165784 Secondary SAM-Like Context

SECONDARY/CONTEXTUAL; CANNOT RESCUE ECM

GPNMB, SPP1, LIPA, and FABP5 were evaluated separately from the primary direct-ECM programme. A gene required both higher raw CPM and higher detection in at least four public samples; a contextual state required at least three coherent genes.

Sample-level SAM-like effects

Cluster	public_unit	present_locked	Target	delta_raw_CPM	delta_raw_ detection	both_higher
0	GSM5049905	TRUE	GPNMB	-647.8704	0.0733	FALSE
0	GSM5049905	TRUE	SPP1	-69.3871	0.2263	FALSE
0	GSM5049905	TRUE	LIPA	-165.5751	0.0588	FALSE
0	GSM5049905	TRUE	FABP5	-358.1929	0.0753	FALSE
0	GSM5049906	TRUE	GPNMB	-1990.8636	-0.1813	FALSE
0	GSM5049906	TRUE	SPP1	4834.5469	0.3616	TRUE
0	GSM5049906	TRUE	LIPA	-878.0937	-0.2191	FALSE
0	GSM5049906	TRUE	FABP5	-165.4566	-0.0757	FALSE
0	GSM5277737	TRUE	GPNMB	-1411.1352	-0.1190	FALSE
0	GSM5277737	TRUE	SPP1	-7015.5187	0.0627	FALSE
0	GSM5277737	TRUE	LIPA	-444.1316	-0.3062	FALSE
0	GSM5277737	TRUE	FABP5	-127.7311	-0.1337	FALSE
0	GSM5690478	TRUE	GPNMB	-771.8674	-0.3983	FALSE
0	GSM5690478	TRUE	SPP1	-3131.5808	-0.0345	FALSE
0	GSM5690478	TRUE	LIPA	-222.1479	-0.1536	FALSE
0	GSM5690478	TRUE	FABP5	-397.8445	-0.3352	FALSE
0	GSM5690479	TRUE	GPNMB	-1430.3727	-0.0620	FALSE
0	GSM5690479	TRUE	SPP1	-2927.9909	0.0151	FALSE
0	GSM5690479	TRUE	LIPA	-693.5330	-0.1092	FALSE
0	GSM5690479	TRUE	FABP5	-147.4881	-0.0813	FALSE
1	GSM5049905	TRUE	GPNMB	1271.4371	0.4483	TRUE
1	GSM5049905	TRUE	SPP1	-5471.0472	0.1207	FALSE
1	GSM5049905	TRUE	LIPA	412.0472	0.4123	TRUE
1	GSM5049905	TRUE	FABP5	547.5599	0.3310	TRUE
1	GSM5049906	TRUE	GPNMB	3485.4264	0.3925	TRUE

Cluster	public_unit	present_locked	Target	delta_raw_CPM	delta_raw_ detection	both_higher
1	GSM5049906	TRUE	SPP1	-1367.9268	0.0944	FALSE
1	GSM5049906	TRUE	LIPA	1266.1870	0.2708	TRUE
1	GSM5049906	TRUE	FABP5	276.5100	0.3906	TRUE
1	GSM5277737	TRUE	GPNMB	1887.0199	0.3058	TRUE
1	GSM5277737	TRUE	SPP1	-4170.6251	0.1844	FALSE
1	GSM5277737	TRUE	LIPA	490.0327	0.3846	TRUE
1	GSM5277737	TRUE	FABP5	128.3197	0.2831	TRUE
1	GSM5690478	TRUE	GPNMB	648.7212	0.2143	TRUE
1	GSM5690478	TRUE	SPP1	-3721.7093	0.0293	FALSE
1	GSM5690478	TRUE	LIPA	227.3503	0.1262	TRUE
1	GSM5690478	TRUE	FABP5	227.4180	0.2001	TRUE
1	GSM5690479	TRUE	GPNMB	1708.4400	0.1846	TRUE
1	GSM5690479	TRUE	SPP1	-13415.7644	0.0230	FALSE
1	GSM5690479	TRUE	LIPA	540.4471	0.1643	TRUE
1	GSM5690479	TRUE	FABP5	180.3768	0.3122	TRUE
2	GSM5049905	TRUE	GPNMB	-493.5215	0.1043	FALSE
2	GSM5049905	TRUE	SPP1	-1931.0477	0.0241	FALSE
2	GSM5049905	TRUE	LIPA	-157.8740	-0.0947	FALSE
2	GSM5049905	TRUE	FABP5	-587.8883	-0.3801	FALSE
2	GSM5049906	TRUE	GPNMB	-1091.1782	0.1087	FALSE
2	GSM5049906	TRUE	SPP1	-2984.0269	-0.1668	FALSE
2	GSM5049906	TRUE	LIPA	-530.9047	-0.0087	FALSE
2	GSM5049906	TRUE	FABP5	-234.6838	-0.2909	FALSE
2	GSM5277737	TRUE	GPNMB	-1085.2024	-0.0958	FALSE
2	GSM5277737	TRUE	SPP1	-11272.7733	-0.2870	FALSE
2	GSM5277737	TRUE	LIPA	-215.8966	-0.0801	FALSE
2	GSM5277737	TRUE	FABP5	-195.4947	-0.2738	FALSE
2	GSM5690478	TRUE	GPNMB	-671.9415	-0.3247	FALSE
2	GSM5690478	TRUE	SPP1	-181.4466	-0.0990	FALSE
2	GSM5690478	TRUE	LIPA	-169.0930	-0.1455	FALSE

Cluster	public_unit	present_locked	Target	delta_raw_CPM	delta_raw_ detection	both_higher
2	GSM5690478	TRUE	FABP5	-470.6681	-0.4200	FALSE
2	GSM5690479	TRUE	GPNMB	-885.0599	0.0897	FALSE
2	GSM5690479	TRUE	SPP1	-2017.8610	-0.0007	FALSE
2	GSM5690479	TRUE	LIPA	-137.5822	0.0654	FALSE
2	GSM5690479	TRUE	FABP5	-229.3872	-0.3493	FALSE
3	GSM5049905	TRUE	GPNMB	-881.7685	-0.2977	FALSE
3	GSM5049905	TRUE	SPP1	-16874.4817	-0.1360	FALSE
3	GSM5049905	TRUE	LIPA	-162.8189	-0.0556	FALSE
3	GSM5049905	TRUE	FABP5	618.0471	0.1759	TRUE
3	GSM5049906	TRUE	GPNMB	-1884.3422	-0.1426	FALSE
3	GSM5049906	TRUE	SPP1	-4390.8942	-0.3946	FALSE
3	GSM5049906	TRUE	LIPA	-548.3269	-0.0430	FALSE
3	GSM5049906	TRUE	FABP5	-220.5125	-0.1680	FALSE
3	GSM5277737	TRUE	GPNMB	-1179.8197	-0.3001	FALSE
3	GSM5277737	TRUE	SPP1	-12273.0317	-0.3423	FALSE
3	GSM5277737	TRUE	LIPA	-375.9395	-0.1678	FALSE
3	GSM5277737	TRUE	FABP5	-120.7884	-0.1385	FALSE
3	GSM5690478	TRUE	GPNMB	-376.0426	0.0599	FALSE
3	GSM5690478	TRUE	SPP1	-2325.8080	0.0497	FALSE
3	GSM5690478	TRUE	LIPA	-107.3243	0.0223	FALSE
3	GSM5690478	TRUE	FABP5	21.0820	0.1865	TRUE
3	GSM5690479	TRUE	GPNMB	-1116.5390	-0.1596	FALSE
3	GSM5690479	TRUE	SPP1	-12299.0591	-0.0243	FALSE
3	GSM5690479	TRUE	LIPA	-249.2822	-0.0508	FALSE
3	GSM5690479	TRUE	FABP5	234.2925	0.1043	TRUE
4	GSM5049905	TRUE	GPNMB	365.3859	0.4092	TRUE
4	GSM5049905	TRUE	SPP1	27860.1338	0.3449	TRUE
4	GSM5049905	TRUE	LIPA	53.3601	0.2582	TRUE
4	GSM5049905	TRUE	FABP5	-97.1518	0.3091	FALSE
4	GSM5049906	TRUE	GPNMB	-292.4496	0.3061	FALSE

Cluster	public_unit	present_locked	Target	delta_raw_CPM	delta_raw_ detection	both_higher
4	GSM5049906	TRUE	SPP1	7343.5178	0.4490	TRUE
4	GSM5049906	TRUE	LIPA	-306.9080	0.2298	FALSE
4	GSM5049906	TRUE	FABP5	19.2579	0.1760	TRUE
4	GSM5277737	TRUE	GPNMB	-200.7103	0.2375	FALSE
4	GSM5277737	TRUE	SPP1	5103.1646	0.2141	TRUE
4	GSM5277737	TRUE	LIPA	-86.6971	0.0996	FALSE
4	GSM5277737	TRUE	FABP5	49.9425	0.1184	TRUE
4	GSM5690478	TRUE	GPNMB	-58.4298	0.1300	FALSE
4	GSM5690478	TRUE	SPP1	9520.6145	0.0119	TRUE
4	GSM5690478	TRUE	LIPA	4.1129	0.0757	TRUE
4	GSM5690478	TRUE	FABP5	-139.9002	-0.0387	FALSE
4	GSM5690479	TRUE	GPNMB	-322.1362	0.1474	FALSE
4	GSM5690479	TRUE	SPP1	18508.2693	0.0263	TRUE
4	GSM5690479	TRUE	LIPA	106.9215	0.1166	TRUE
4	GSM5690479	TRUE	FABP5	-27.5406	0.1463	FALSE
5	GSM5049905	TRUE	GPNMB	-1141.9472	-0.7396	FALSE
5	GSM5049905	TRUE	SPP1	-18108.2878	-0.5370	FALSE
5	GSM5049905	TRUE	LIPA	-372.0573	-0.5629	FALSE
5	GSM5049905	TRUE	FABP5	-611.7863	-0.4976	FALSE
5	GSM5049906	TRUE	GPNMB	-2121.1152	-0.6692	FALSE
5	GSM5049906	TRUE	SPP1	-4396.2929	-0.4797	FALSE
5	GSM5049906	TRUE	LIPA	-753.9318	-0.2607	FALSE
5	GSM5049906	TRUE	FABP5	-234.4833	-0.3589	FALSE
5	GSM5277737	TRUE	GPNMB	-1900.4857	-0.8127	FALSE
5	GSM5277737	TRUE	SPP1	-15191.2808	-0.6773	FALSE
5	GSM5277737	TRUE	LIPA	-489.3870	-0.4113	FALSE
5	GSM5277737	TRUE	FABP5	-232.9033	-0.3299	FALSE
5	GSM5690478	TRUE	GPNMB	-945.2545	-0.6120	FALSE
5	GSM5690478	TRUE	SPP1	-9704.0170	-0.2702	FALSE
5	GSM5690478	TRUE	LIPA	-386.1003	-0.5252	FALSE

Cluster	public_unit	present_locked	Target	delta_raw_CPM	delta_raw_ detection	both_higher
5	GSM5690478	TRUE	FABP5	-374.2889	-0.5258	FALSE
5	GSM5690479	TRUE	GPNMB	-1754.7882	-0.5775	FALSE
5	GSM5690479	TRUE	SPP1	-13696.5689	-0.1150	FALSE
5	GSM5690479	TRUE	LIPA	-734.2214	-0.4124	FALSE
5	GSM5690479	TRUE	FABP5	-103.1412	-0.3475	FALSE
6	GSM5049905	TRUE	GPNMB	767.6129	0.3198	TRUE
6	GSM5049905	TRUE	SPP1	27561.2824	0.3391	TRUE
6	GSM5049905	TRUE	LIPA	55.6469	0.1125	TRUE
6	GSM5049905	TRUE	FABP5	13.0293	0.1567	TRUE
6	GSM5049906	TRUE	GPNMB	684.3711	0.2574	TRUE
6	GSM5049906	TRUE	SPP1	5202.4038	0.2913	TRUE
6	GSM5049906	TRUE	LIPA	310.3555	0.1060	TRUE
6	GSM5049906	TRUE	FABP5	336.2461	0.3282	TRUE
6	GSM5277737	TRUE	GPNMB	180.1433	0.2419	TRUE
6	GSM5277737	TRUE	SPP1	29201.2965	0.2757	TRUE
6	GSM5277737	TRUE	LIPA	122.2496	0.3096	TRUE
6	GSM5277737	TRUE	FABP5	133.3741	0.2648	TRUE
6	GSM5690478	TRUE	GPNMB	1158.9742	0.1866	TRUE
6	GSM5690478	TRUE	SPP1	8788.2624	0.0654	TRUE
6	GSM5690478	TRUE	LIPA	176.3880	0.0554	TRUE
6	GSM5690478	TRUE	FABP5	627.7440	0.2191	TRUE
6	GSM5690479	TRUE	GPNMB	895.6858	0.1733	TRUE
6	GSM5690479	TRUE	SPP1	28232.6672	0.0327	TRUE
6	GSM5690479	TRUE	LIPA	187.3828	0.1161	TRUE
6	GSM5690479	TRUE	FABP5	-27.7875	0.2033	FALSE

SAM-like gene coherence

Cluster	Target	n_samples_present_ locked	samples_both_ higher	median_delta_raw_ CPM	median_delta_raw_ detection	sam_gene_coherent
0	GPNMB	5	0	-1411.1352	-0.1190	FALSE
0	SPP1	5	1	-2927.9909	0.0627	FALSE
0	LIPA	5	0	-444.1316	-0.1536	FALSE
0	FABP5	5	0	-165.4566	-0.0813	FALSE
1	GPNMB	5	5	1708.4400	0.3058	TRUE
1	SPP1	5	0	-4170.6251	0.0944	FALSE
1	LIPA	5	5	490.0327	0.2708	TRUE
1	FABP5	5	5	227.4180	0.3122	TRUE
2	GPNMB	5	0	-885.0599	0.0897	FALSE
2	SPP1	5	0	-2017.8610	-0.0990	FALSE
2	LIPA	5	0	-169.0930	-0.0801	FALSE
2	FABP5	5	0	-234.6838	-0.3493	FALSE
3	GPNMB	5	0	-1116.5390	-0.1596	FALSE
3	SPP1	5	0	-12273.0317	-0.1360	FALSE
3	LIPA	5	0	-249.2822	-0.0508	FALSE
3	FABP5	5	3	21.0820	0.1043	FALSE
4	GPNMB	5	1	-200.7103	0.2375	FALSE
4	SPP1	5	5	9520.6145	0.2141	TRUE
4	LIPA	5	3	4.1129	0.1166	FALSE
4	FABP5	5	2	-27.5406	0.1463	FALSE
5	GPNMB	5	0	-1754.7882	-0.6692	FALSE
5	SPP1	5	0	-13696.5689	-0.4797	FALSE
5	LIPA	5	0	-489.3870	-0.4124	FALSE
5	FABP5	5	0	-234.4833	-0.3589	FALSE
6	GPNMB	5	5	767.6129	0.2419	TRUE
6	SPP1	5	5	27561.2824	0.2757	TRUE
6	LIPA	5	5	176.3880	0.1125	TRUE
6	FABP5	5	4	133.3741	0.2191	TRUE

Contextual state decisions

Cluster	coherent_SAM_genes	SAM_LIKE_CONTEXT_PASS
0	0	FALSE
1	3	TRUE
2	0	FALSE
3	0	FALSE
4	1	FALSE
5	0	FALSE
6	4	TRUE

Table S9. Reproducibility and Correction History

REPRODUCIBILITY RECORD

Target-feature mappings, software versions, implementation amendments and corrections, and provenance relevant to the reported analyses.

GSE294329 target-feature mapping

locked_slot	resolved_feature	Role
COL1A1	COL1A1	PRIMARY
COL1A2	COL1A2	PRIMARY
COL5A1	COL5A1	PRIMARY
LUM	LUM	PRIMARY
THBS2	THBS2	PRIMARY
FBLN2	FBLN2	PRIMARY
CCN2_or_CTGF	CCN2	PRIMARY
FN1	FN1	SECONDARY
SPARC	SPARC	SECONDARY

SCP2582 target-feature mapping

locked_slot	resolved_feature	Role
COL1A1	COL1A1	PRIMARY_ASSAYABLE
COL1A2	COL1A2	PRIMARY_ASSAYABLE
COL5A1	COL5A1	PRIMARY_ASSAYABLE
LUM	LUM	PRIMARY_ASSAYABLE
THBS2	THBS2	PRIMARY_ASSAYABLE
FBLN2	FBLN2	PRIMARY_ASSAYABLE
CCN2_or_CTGF		PRIMARY_UNAVAILABLE
FN1	FN1	SECONDARY
SPARC	SPARC	SECONDARY

GSE165784 target-feature mapping

locked_slot	resolved_feature	Role
COL1A1	COL1A1	PRIMARY_ECM
COL1A2	COL1A2	PRIMARY_ECM

locked_slot	resolved_feature	Role
COL5A1	COL5A1	PRIMARY_ECM
LUM	LUM	PRIMARY_ECM
THBS2	THBS2	PRIMARY_ECM
FBLN2	FBLN2	PRIMARY_ECM
CCN2_or_CTGF	CTGF	PRIMARY_ECM
FN1	FN1	SECONDARY_ECM
SPARC	SPARC	SECONDARY_ECM
GPNMB	GPNMB	SECONDARY_SAM_CONTEXT
SPP1	SPP1	SECONDARY_SAM_CONTEXT
LIPA	LIPA	SECONDARY_SAM_CONTEXT
FABP5	FABP5	SECONDARY_SAM_CONTEXT

Recorded software environment

Component	Version	Evidence
R	4.6.1	All stage sessionInfo records
Operating system	Windows 10 x64 (build 19045)	All stage sessionInfo records
Seurat	5.5.1	Stage sessionInfo records
SeuratObject	5.4.0	Stage sessionInfo records
Matrix	1.7-6	Stage sessionInfo records
data.table	1.18.4	Stage sessionInfo records
ggplot2	4.0.3	Stage sessionInfo records
scDblFinder	1.26.7	Target-blind preprocessing sessionInfo records
celda	1.28.0	Target-blind preprocessing sessionInfo records
SingleCellExperiment	1.34.0	Target-blind preprocessing sessionInfo records
SummarizedExperiment	1.42.0	Target-blind preprocessing sessionInfo records
BiocParallel	1.46.0	Unblinding/adjudication sessionInfo records
UCell	2.16.0	GSE294329 Stage 2A/2B and SCP2582 Stage 3C sessionInfo records
GEOquery	2.80.0	GSE165784 Stage 5A/5B sessionInfo records

Implementation and correction history

Item	Classification	Handling	Effect on scientific specification
SCP2582 Stage 3A v1.0.1	Implementation patch	Run FindClusters once per resolution with explicit metadata names; reuse completed donor post-QC/DecontX objects	None
SCP2582 Stage 3B v1.0.1	Compatibility patch	Use k.weight=50 for small-anchor integration	None
GSE294329 Stage 2B v1.0.1	Compatibility patch	Handle UCell matrix return and Seurat v5 layers	None
GSE294329 expression correction v1.0	Post-unblinding correction/addendum	Compute CPM before adding common CPM-scale offset; quantify library complexity	Candidate, thresholds, and Stage 2A record unchanged
GSE165784 Stage 5A v1.0.1	Manifest compatibility patch	Windows-safe relative-path handling	None
GSE165784 direct-ECM gate	Plotting correction	Criterion C dashed line corrected from four to three coherent genes	No numerical decision or interpretation changed
Legacy GSE294329 pseudobulk outputs	Superseded	Exclude obsolete pseudobulk gene-direction, target-delta and module-coherence summaries generated before the CPM-order correction.	Replaced by CPM-first correction outputs
SCP2582 archived outcome-summary wording	Known internal text inconsistency	Do not quote 'Exact six-gene replication evaluable: FALSE'	Use feature mapping and decision output: exact seven-gene not evaluable; amended six-gene evaluable and negative

Additional Exploratory Sensitivity and Diagnostic Analyses

These analyses were conducted after the prespecified primary analyses. They used archived cell populations, frozen state labels and donor/sample-level summaries; where specified, alternative DecontX processing, feature selection or reclustering was applied to the same fixed cells. They are exploratory and do not replace or modify the original ECM7 discovery rule, the amended SCP2582 six-gene replication gate, or the GSE165784 direct-ECM and SAM-like decisions.

Table S10. Exploratory Structural Collagen CPM Contrasts

The three prespecified structural collagen targets were COL1A1, COL1A2 and COL5A1. For each frozen state and biological or public-sample unit, the raw CPM values for these genes were summed in the state and in its within-unit myeloid comparator. Rounded-DecontX CPM was summed separately. A positive contrast denotes greater within-unit collagen CPM in the state. The denominator is identical for each gene within a state and unit, so summation is valid for CPM; it does not recover the fraction of cells detecting any collagen.

Cohort	State	Units	Raw CPM higher	Rounded CPM higher	Median Δ CPM [range]
GSE165784	0	5	4/5	0/5	1.49 [-1.67, 58.79]
GSE165784	1	5	0/5	0/5	-8.42 [-38.33, -2.33]
GSE165784	2	5	3/5	0/5	5.95 [-3.98, 14.52]
GSE165784	3	5	1/5	0/5	-3.75 [-19.36, 1.84]
GSE165784	4	5	4/5	0/5	4.28 [-36.03, 14.91]
GSE165784	5	5	4/5	3/5	9.46 [-3.88, 94.16]
GSE165784	6	5	5/5	0/5	6.47 [1.14, 55.60]
GSE294329	1	5	4/5	4/5	35.70 [-11.39, 58.83]
SCP2582	0	5	5/5	2/5	6.40 [0.27, 11.76]
SCP2582	1	5	4/5	2/5	4.95 [-10.10, 15.67]
SCP2582	2	5	1/5	0/5	-6.71 [-26.12, 2.13]
SCP2582	3	4	1/4	1/4	-7.65 [-38.02, 7.61]
SCP2582	4	4	2/4	2/4	2.42 [-33.71, 10.01]

GSE294329 cluster 1: raw and rounded collagen CPM were higher in 4/5 donors, but none of the three genes had higher raw detection in any donor. SCP2582 cluster 0: raw collagen CPM was higher in 5/5 donors and rounded CPM in 2/5; COL1A1 alone had both higher raw CPM and detection in 4/5 donors. GSE165784 cluster 6: raw collagen CPM was higher in 5/5 public PDR accessions and rounded CPM in 0/5; no collagen gene had both higher raw CPM and detection in 4/5 accessions. These differences cannot be described as structural-collagen replication because no structural-only detection union or prospectively defined collagen gate exists. The five PDR accessions may include repeat samples from three patients.

These exploratory descriptive contrasts are summarised in Table S10 at the biological-donor or public-sample level. No significance tests were performed.

Table S11. Frozen Cluster Inventory

Cell counts and unit ranges are transcribed or aggregated from target-blind cluster composition records. Marker lists come from the archived target-blind Seurat FindAllMarkers outputs and are descriptive; cell-level marker P values are not used as donor-level evidence. A displayed unit can contain fewer cells than the locked presence threshold. Clusters with the same number in separate cohorts are not assumed homologous.

Cohort	State	Gate eligible	Cells	Units (cell range)	Leading non-target markers
GSE165784	0	Yes	1048	5 (66-406)	RGS1, CD74, C3, HLA-DRA, CX3CR1, HLA-DPA1
GSE165784	1	Yes	964	5 (147-306)	FTL, LGALS3, CTSD, PRDX1, MGLL, ACP5
GSE165784	2	Yes	681	5 (60-304)	SELENOP, MRC1, MAF, FOLR2, F13A1, SLC40A1
GSE165784	3	Yes	673	5 (24-203)	CLEC10A, VCAN, FCN1, PTMA, IFI6, AIF1

Cohort	State	Gate eligible	Cells	Units (cell range)	Leading non-target markers
GSE165784	4	Yes	565	5 (79-187)	TMIGD3, NUPR1, TREM2, VSIG4, ANXA2, APOC1
GSE165784	5	Yes	538	5 (25-181)	S100A12, APOBEC3A, CFP, FCN1, VCAN, RIPOR2
GSE165784	6	Yes	432	5 (38-207)	EGLN3, HMOX1, MT1G, CTSB, SLC2A1, GCLM
GSE165784	7	No	110	5 (6-48)	CXCL11, CXCL9, PLA2G2D, SLAMF7, CHIT1, CXCL10
GSE165784	8	No	94	4 (2-53)	GFRA2, SPIC, PDK4, CXCL12, VCAM1, CD163L1
GSE294329	0	Yes	1039	5 (109-302)	CYB5D1, BAG3, ARL5B, TNFAIP3, HSPH1, KDM6B
GSE294329	1	Yes	892	5 (77-262)	TMSB4X, B2M, CD74, C1QB, TPT1, TTR
GSE294329	2	Yes	679	5 (91-206)	EGR1, FOS, TTR, SPATA13, TF, ADAM28
GSE294329	3	Yes	630	5 (74-222)	COLEC12, SLC16A10, FAM20C, PELATON, FLNA, TNS1
GSE294329	4	Yes	590	5 (69-208)	CPVL, FCGBP, TM9SF2, LRP1, MALAT1, RSRP1
GSE294329	5	Yes	564	5 (83-168)	TMSB4X, FTL, TPT1, EEF1A1, RPL23, RPL34
GSE294329	6	No	99	5 (8-55)	FCER1A, CD1E, CD1C, CLEC10A, FLT3, PLAC8
GSE294329	7	No	71	5 (3-31)	CXCL10, RRM2, CXCL9, CXCL11, TK1, GINS2
SCP2582	0	Yes	496	5 (40-188)	CCL3, CD83, IL1B, PPP1R15A, CCL4, RPL3
SCP2582	1	Yes	234	5 (19-65)	RNASE1, PSAP, KCTD12, DAB2, MT-ND2, MRC1
SCP2582	2	Yes	179	5 (10-80)	SH3BGRL3, FBP1, CYP19A1, S100A6, ANXA2, VIM
SCP2582	3	Yes	131	5 (6-63)	OTOA, FTL, CTSD, CSTB, LGALS3, CHIT1
SCP2582	4	Yes	105	5 (6-37)	FOS, JUN, KLF2, SGK1, CEBPD, HLA-DRB1
SCP2582	5	No	105	5 (6-50)	CD3E, TRBC2, CD3D, SPOCK2, GZMA, CD3G
SCP2582	6	No	31	5 (1-9)	CD1C, FCER1A, CD1E, CLEC4F, AFF3, PLAC8

The marker lists permit tentative interpretation only. For instance, GSE294329 cluster 1 has C1QB/CD74 expression and low library complexity; SCP2582 cluster 0 includes CCL3/IL1B; GSE165784 PDR cluster 6 includes EGLN3/HMOX1 and met the separately defined SAM-like rule. These observations do not demonstrate cross-cohort homology or stable macrophage subtypes. Formal transfer/trajectory and orthogonal spatial or protein data are unavailable.

Table S12. Exploratory SCP2582 DecontX Sensitivity and Raw Gate Checks

This exploratory sensitivity analysis held the 1,281 frozen myeloid cell identities fixed, reproduced all original clustering labels and raw A/B/C gate counts in a positive control, and compared DecontX refits with eight protected genes excluded. State labels are matched by maximum cell overlap because cluster numbers have no inherent equivalence. The original and alternative integration feature lists overlap at 1,967/2,000; neither list directly includes a protected target. The adjusted Rand index is 0.440 (cell agreement after label matching: 844/1,281). Cells are not treated as independent donors.

S12A Cluster movement by PVR donor

Donor	Frozen cells	Moved after matching
PVR-22	271	94
PVR-24	349	96
PVR-25	395	144
PVR-29	178	76
PVR-31	88	27

S12B Alternative raw RNA gates

Cluster	Cells	Donors present	Eligible	A donors	B donors	C genes
0	490	5	Yes	3	2	1
1	222	5	Yes	4	2	0
2	198	5	Yes	1	1	0
3	194	5	Yes	2	2	0
4	82	4	Yes	3	0	0
5	71	2	No	0	0	0
6	24	1	No	1	0	0

A and B require a positive within-donor contrast in at least four of five PVR donors; C requires at least three genes each with both higher raw CPM and detection in at least four donors. Only COL1A2 in alternative cluster 0 met that gene-level criterion; all other cluster-by-gene entries failed it. The original cluster-eligibility rule was applied to the alternative clusters for description only. Because the alternative corrected count matrices exclude the target genes, D, the rounded-DecontX target CPM criterion, cannot be recomputed. Broad lineage assignment and other cohorts were not reanalysed. Hence S12 is neither an exact alternative four-gate replication nor a modification of the frozen decision.

Reproducibility note and outstanding source material

The compact repository contains executed scripts and most stage summaries, with missing large frozen objects still limiting a full independent rerun. The separately retrieved frozen SCP2582 myeloid object, five singlet matrices and locally inspected original processed source object supported the diagnostics shown here. None of these exploratory checks timestamps the initial freeze, assesses broad lineage selection or changes Tables S1–S9. The original source object did not contain CCN2/CTGF; why the features were absent before deposition remains unknown.

Table S13. Exploratory Conventional Fixed-Cell Clustering and Raw Gate Checks

Original-workflow control recovered all 1,281 frozen labels and archived raw A/B/C results exactly. In a separate conventional rerun permitting protected features, 1,999/2,000 integration features were shared and only FN1 (a secondary marker) was newly selected among protected features. Numerical labels changed for 148/1,281 cells; adjusted Rand index 0.721. Each row below represents an alternative cluster compared within donor with other fixed myeloid cells. A and B require at least four of five donors; C requires at least three genes each with concordant raw CPM and detection in at least four donors. D is not retested; these clusters are exploratory.

Cluster	Cells	Donors	Eligible	A donors	B donors	C genes
0	443	5	Yes	5	2	0
1	294	5	Yes	3	2	2
2	163	4	Yes	2	1	0
3	136	4	Yes	1	1	0
4	120	4	Yes	2	2	0
5	94	3	No	2	0	0
6	31	0	No	0	0	0

This fixed-compartment sensitivity neither tests whether target genes influence broad lineage selection nor supplies a complete amended six-gene four-gate result. The table summarises the diagnostic results; complete cell-level labels and gene-by-donor outputs are not reproduced here.

Table S14. Exploratory Discovery UCell Rank Diagnostic

Locked unrounded-DecontX discovery contrasts were reproduced in each of five PVR donors (maximum numerical difference <10^−12). Effects below equal mean cluster-1 UCell score minus the within-donor mean in other frozen myeloid cells; they are exploratory diagnostic checks and do not replace the frozen discovery rule. Complexity is reported as median raw UMI counts for candidate / comparator cells. Scores came from raw, unrounded-DecontX and rounded-DecontX matrices on the same frozen cells.

Donor	Median raw UMIs candidate / other	Mean raw target UMIs candidate / other	Raw UCell Δ	Unrounded Δ	Rounded Δ
JT06	777/2156.5	0.111/0.586	+0.00271	+0.00308	+0.00081
NPC05	882/10613	0.054/0.477	+0.00163	+0.00243	+0.00129
WHN03	840/8086	0.092/0.485	+0.00106	+0.00122	-0.00065
XZH02	936/12054	0.273/1.732	+0.00254	+0.00680	-0.00325
ZWB04	805/5366.5	0.052/0.509	-0.00314	-0.00113	-0.00064

Positive per-cell scaling and log1p CPM are monotone and preserve within-cell positive-expression gene ranks; DecontX and integer rounding need not. Gene-rank summaries use positive-gene midpoint proxies capped at 1,501 for the locked maxRank of 1,500. The diagnostic recorded cell-level detection, capped-rank, fractional-only and library-complexity quantities; selected summaries are presented here. A controlled toy calculation held seven target counts fixed:

Illustrative cell	Target counts	Stronger background genes	UCell score
few_background	7	100	0.933
many_background	7	1800	0.000

The toy calculation illustrates a rank-based possibility, not an estimated effect or proof that background complexity caused the observed discovery pattern. Cells are descriptive measurements; inference units remain five donors.

Table S15. Original SCP2582 Processed Feature Provenance

The original processed SCP2582 Seurat object contained 6,372 cells and 17,417 RNA feature names, with Seurat variable-feature metadata. The archived PVR-31 post-QC singlet object had the same 17,417 RNA feature names. Feature-name presence was checked without inspecting gene expression. Both CCN2 and CTGF were missing already in the original processed source object.

Gene symbol	Original processed source	PVR-31 post-QC
COL1A1	Present	Present
COL1A2	Present	Present
COL5A1	Present	Present
LUM	Present	Present
THBS2	Present	Present
FBLN2	Present	Present
CCN2	Absent	Absent
CTGF	Absent	Absent

Gene symbol	Original processed source	PVR-31 post-QC
FN1	Present	Present
SPARC	Present	Present

This audit locates absence before the supplied processed source object; it does not identify whether the original platform, annotation, or preprocessing before that object removed CCN2/CTGF. The audit also examined the complete 17,417-row source feature-name and feature-metadata records. Exact seven-gene replication remains unevaluable; the earlier six-gene amendment and endpoint hierarchy are unchanged.

Table S16. Exploratory NABA Collagen-Panel Sensitivity

The independently published NABA_COLLAGENS set (MSigDB M3005, Naba et al. 2012; https://www.gsea-msigdb.org/gsea/msigdb/human/geneset/NABA_COLLAGENS.html) contains 44 genes. We also examined 41 genes after excluding the original ECM7 collagens COL1A1, COL1A2 and COL5A1. All mapped in GSE294329; SCP2582 lacked COL6A5 (43 and 40 assayable genes, respectively). COL6A5 had zero raw CPM in the GSE294329 discovery comparisons. Frozen GSE294329 ECM7 detection and SCP2582 six-gene raw A/B directions were reproduced. No new gate or P value was defined.

S16A Donor direction summary for frozen PVR states

Positive counts compare a frozen state with other myeloid cells in each donor. Raw detection is the fraction of cells with any assayable collagen; corrected CPM uses rounded DecontX counts. SCP2582 clusters 3 and 4 met frozen eligibility in four donors. The inference units are donors, not cells.

PVR cohort	Cluster	Panel	Genes assayable	Donors	Raw CPM +	Any detect +	Rounded CPM +
GSE294329	1	44-gene	44/44	5	4	0	3
GSE294329	1	Exclude 3	41/41	5	2	0	2
SCP2582	0	44-gene	43/44	5	2	2	2
SCP2582	0	Exclude 3	40/41	5	2	2	2
SCP2582	1	44-gene	43/44	5	4	3	3
SCP2582	1	Exclude 3	40/41	5	5	4	3
SCP2582	2	44-gene	43/44	5	2	3	4
SCP2582	2	Exclude 3	40/41	5	3	4	4
SCP2582	3	44-gene	43/44	4	1	2	2
SCP2582	3	Exclude 3	40/41	4	2	3	3
SCP2582	4	44-gene	43/44	4	1	0	0
SCP2582	4	Exclude 3	40/41	4	0	0	0

S16B Direction and magnitude in the nominated or prominent states

Within-donor contrasts for GSE294329 candidate cluster 1 and exploratory SCP2582 cluster 1 use the 41-gene panel (40 available in SCP2582). The SCP2582 cluster was one of five eligible states examined in this exploratory sensitivity analysis; positive directions do not establish a replicated state.

Cohort	Donor	State cells	Raw Δ CPM	Raw Δ detection	Rounded Δ CPM
GSE294329	JT06	262	+43.67	-0.313	-17.73
GSE294329	NPC05	147	+19.58	-0.394	+17.64
GSE294329	WHN03	251	-6.10	-0.451	-21.75
GSE294329	XZH02	77	-11.55	-0.580	+34.55
GSE294329	ZWB04	155	-52.16	-0.397	-76.03
SCP2582	PVR-22	53	+4.92	+0.004	-3.18
SCP2582	PVR-24	50	+18.37	+0.019	+23.38
SCP2582	PVR-25	65	+4.12	+0.056	+4.56
SCP2582	PVR-29	47	+5.95	-0.024	-0.62
SCP2582	PVR-31	19	+1.58	+0.015	+2.51

COL8A2 and COL24A1 each had higher raw CPM and detection in four SCP2582 donors. COL24A1-positive cluster-1 cells numbered 2/53, 1/50, 5/65 and 1/47 (fifth donor 0/19). Transcripts do not establish collagen-protein secretion. Feature mapping and donor/gene outputs informed this analysis; only the summaries reported here form part of these supplementary tables. Original Tables S1–S9 and endpoint decisions remain unchanged.
